# Behavioural and Developmental Interventions for Autism Spectrum Disorder: A Clinical Systematic Review

**DOI:** 10.1371/journal.pone.0003755

**Published:** 2008-11-18

**Authors:** Maria B. Ospina, Jennifer Krebs Seida, Brenda Clark, Mohammad Karkhaneh, Lisa Hartling, Lisa Tjosvold, Ben Vandermeer, Veronica Smith

**Affiliations:** 1 Alberta Research Centre for Health Evidence, University of Alberta, Edmonton, Alberta, Canada; 2 Department of Pediatrics, Faculty of Medicine and Dentistry, University of Alberta, Edmonton, Alberta, Canada; 3 Department of Educational Psychology, Faculty of Education, University of Alberta, Edmonton, Alberta, Canada; CHEO Research Institute, Canada

## Abstract

**Background:**

Much controversy exists regarding the clinical efficacy of behavioural and developmental interventions for improving the core symptoms of autism spectrum disorders (ASD). We conducted a systematic review to summarize the evidence on the effectiveness of behavioural and developmental interventions for ASD.

**Methods and Findings:**

Comprehensive searches were conducted in 22 electronic databases through May 2007. Further information was obtained through hand searching journals, searching reference lists, databases of theses and dissertations, and contacting experts in the field. Experimental and observational analytic studies were included if they were written in English and reported the efficacy of any behavioural or developmental intervention for individuals with ASD. Two independent reviewers made the final study selection, extracted data, and reached consensus on study quality. Results were summarized descriptively and, where possible, meta-analyses of the study results were conducted. One-hundred-and-one studies at predominantly high risk of bias that reported inconsistent results across various interventions were included in the review. Meta-analyses of three controlled clinical trials showed that Lovaas treatment was superior to special education on measures of adaptive behaviour, communication and interaction, comprehensive language, daily living skills, expressive language, overall intellectual functioning and socialization. High-intensity Lovaas was superior to low-intensity Lovaas on measures of intellectual functioning in two retrospective cohort studies. Pooling the results of two randomized controlled trials favoured developmental approaches based on initiative interaction compared to contingency interaction in the amount of time spent in stereotyped behaviours and distal social behaviour, but the effect sizes were not clinically significant. No statistically significant differences were found for: Lovaas versus special education for non-verbal intellectual functioning; Lovaas versus Developmental Individual-difference relationship-based intervention for communication skills; computer assisted instruction versus no treatment for facial expression recognition; and TEACCH versus standard care for imitation skills and eye-hand integration.

**Conclusions:**

While this review suggests that Lovaas may improve some core symptoms of ASD compared to special education, these findings are based on pooling of a few, methodologically weak studies with few participants and relatively short-term follow-up. As no definitive behavioural or developmental intervention improves all symptoms for all individuals with ASD, it is recommended that clinical management be guided by individual needs and availability of resources.

## Introduction

Autism spectrum disorders (ASD) are neurodevelopmental disorders characterized by a triad of deficits involving communication, reciprocal social interaction, and restricted and repetitive patterns of behaviour, interests and activities [1]. In addition to these core features, a range of other behaviour problems are common, such as anxiety, depression, sleeping and eating disturbances, attention issues, temper tantrums, and aggression or self-injury [2]. Autism is classified within a clinical spectrum of disorders known as pervasive developmental disorders, as defined in the Diagnostic and Statistical Manual of Mental Disorders and the International Statistical Classification of Diseases and Related Health Problems. The spectrum includes conditions such as Autistic Disorder, Asperger’s syndrome, Atypical Autism, and Pervasive Developmental Disorder Not Otherwise Specified [3]. In clinical practice, professionals may use different terms interchangeably to refer to children with similar presentations. While there are no definitive medical tests to indicate the presence of any form of ASD, diagnosis can be made by three years of age based on the presence or absence of specific behaviours that are used as diagnostic criteria. Prevalence estimates indicate that between 10 and 15 of every 10,000 children are autistic [1], [4] but possibly greater than 20 of every 10,000 children have dysfunction which warrants diagnosis at any point along the spectrum [5], [6]. Common comorbidities include mental retardation (Intelligence quotient (IQ) <70) and epilepsy, which are associated with 70% and 25% of autism cases, respectively [7], [8]. While no known cure for ASD exists, the general agreement is that early diagnosis followed by appropriate treatment can improve outcomes in later years for most individuals [9]. Consequently, the question of how various interventions may help to increase the individual’s ability to function is highly relevant to families, health professionals, and policy makers.

Over the past 20 years, a variety of therapies have been proposed to improve the symptoms associated with ASD. Current treatments include pharmacological therapies and various complementary therapies including diet modifications, vitamin therapy, occupational therapy, speech and language therapy and behavioural and developmental approaches [10]. Interventions that fall within the continuum of behavioural and developmental interventions have become the predominant treatment approach for promoting social, adaptive and behavioural function in children with ASD based on efficacy demonstrated in empirical studies. These interventions may be viewed in terms of their position on a continuum from highly structured discrete trial training behavioural approaches guided by a therapist, to social pragmatic approaches where teaching follows the child’s interests and is embedded in daily activities in a natural environment. While therapy may be provided for up to 40 hours per week, controversy exists regarding the intensity required to achieve positive outcomes and the efficacy of one approach compared to another. An umbrella review of systematic reviews of behavioural and developmental interventions for ASD [11] has found that most systematic reviews have methodological weaknesses which make them vulnerable to bias and compromise their validity. There is evidence of positive outcomes for many of the interventions examined in systematic reviews of ASD and therefore, there is a need for further systematic reviews on the effectiveness of behavioural and developmental interventions for ASD which adhere to strict scientific methods.

Clinicians, educators and families of individuals with ASD need to make informed decisions regarding treatment options and therefore, a host of clinical and research questions regarding the benefits of these interventions still need to be clarified and addressed. Considering the importance of, and demand for, behavioural interventions for ASD, as well as the current rising trend in new programs, a rigorous synthesis of high quality evidence regarding the effect of a continuum of behavioural and developmental interventions for ASD will provide much needed information for health care professionals, policy makers, researchers, and families. This systematic review was conducted in order to identify, appraise, and synthesize the evidence on the effects of a continuum of behavioural and developmental interventions for improving core symptoms associated with ASD.

## Methods

### Search Strategy

The systematic review followed a prospective protocol that was developed a priori. Peer-reviewed comprehensive searches were conducted up to May 2007 in 22 psychological, educational and biomedical electronic databases for commercially published literature, as well as dissertations, and conference abstracts (e.g., MEDLINE®, EMBASE, ERIC, CINAHL®, Cochrane Central Register of Controlled Trials, ProQuest Dissertations and Theses, PsycINFO®, BIOSIS Previews®, and Web of Science®). We identified additional studies by contacting experts in the field and by searching reference lists of primary studies, review articles, and textbook chapters. Details of the complete search strategies are available in Supplement S1.

### Study Selection

Studies were included if they were: randomized controlled trials (RCTs), controlled clinical trials (CCTs) or observational analytical studies (i.e., prospective or retrospective cohort studies with comparison groups); published in English; and reported data on the effects of a behavioural or developmental intervention in individuals with ASD. Individuals with Rett's disorder or Childhood Disintegrative Disorder were not considered for this review as they do not conventionally fall within ASD due to their significantly different clinical course. Studies involving participants with dual diagnoses (i.e., any ASD plus attention deficit/hyperactivity disorder, obsessive compulsive disorder, or learning problems) were also considered for inclusion. The primary outcome of interest was the change in core features of ASD (i.e., communication, reciprocal social interaction, and restricted and repetitive patterns of behaviour, interests and activities) as indicated in the Diagnostic and Statistical Manual of Mental Disorders criteria [1]. Other outcomes that were examined included changes in non-core behaviours, developmental changes, cognitive changes, adaptive behaviours, challenging behaviours, play skills, educational performance, and family-related outcomes. One reviewer screened titles and abstracts of potentially relevant studies. Inclusion criteria were applied independently by at least two reviewers. The primary reason for exclusion of articles was documented. A complete list of excluded studies and reasons for exclusion are available in Supplement S2.

### Quality Assessment and Data Abstraction

Two reviewers independently assessed the methodological quality of the studies. Disagreements were resolved by consensus. We assessed the methodological quality of the studies with two pre-tested checklists (one for clinical trials and the other for observational studies) that included items from other published scales and checklists [12]–[18]; these items address specific aspects of design, execution, and analysis of the studies. The trials checklist included questions related to bias reduction such as allocation concealment [19], [20], randomization, blinding (subject, provider, and outcome assessor blinding), and description of dropouts and withdrawals [21], [22]. Other variables that were evaluated included description of selection criteria, therapeutic regimens, intervention providers, and treatment fidelity. The checklist for the observational studies included items that evaluated the methods of selection of exposed and non-exposed cohorts, ascertainment of outcome and exposure, and how the study handled confounders in the design or analysis. Finally, information regarding the source of funding was collected [23]. Information regarding the study design and methods, the characteristics of participants, interventions, comparison groups, and outcomes of interest were extracted using a pre-tested data extraction form. One reviewer extracted the data using a pre-tested form, and a second reviewer verified the accuracy and completeness of the data. Discrepancies in data extraction were resolved by consensus between the data extractor and the data verifier. Interventions were categorized based on a classification scheme previously described by other researchers in this field [24].

### Analysis and Presentation of Results

There is considerable overlap between and across various models to classify and describe interventions that fall within the continuum of behavioural and developmental interventions for ASD [25], [26]. Due to the absence of a unique classification system, an intervention taxonomy system was developed for the purposes of the review in order to categorize the interventions for the analysis. Each study that met the selection criteria was reviewed and classified according to the continuum of behavioural and developmental interventions described in the scientific literature. The coding categories were based, in part, on a classification scheme previously described by other researchers in this field [24]. Additional categories were added after consultation with a panel of experts. Two independent researchers coded each study. Coding was discussed between researchers on a study-by-study basis and discrepancies were resolved by consensus.

Results were summarized descriptively. Evidence tables were used to report information on study design, study population, treatment groups, outcomes, and results. Due to the limited number of interventions and outcomes available for meta-analysis, we attempted to identify patterns across individual study results. Where studies within an intervention category produced inconsistent results and conclusions, we examined the following variables to shed light on reasons for the discrepant findings: study design, length of follow-up, sample size, population characteristics (age, diagnosis), comparison, and outcomes.

We conducted a meta-analysis when two or more trials assessed the same intervention, used similar comparison groups, and had data for common outcomes of interest. If the same measure was reported, we used weighted mean differences (WMD) and 95% confidence intervals (95% CI); otherwise, we used standardised mean differences (SMD) and 95% CI. Hedges adjusted g was used as the standard deviation estimate for the SMD [27]. A SMD of 0.2 indicated a small effect, 0.5 a medium effect and 0.8 a large effect size [28]. Random effect models were used throughout to combine study results. If means or standard deviations were not reported, they were imputed from other information reported in the study. Heterogeneity was investigated using the chi-square test [29] and quantified with the I^2^ statistic [30]. Heterogeneity was characterized as small (I^2^ less than 25 percent), moderate (I^2^ between 26 and 74 percent) and high (75 percent and above) [30]. Sources of heterogeneity were explored qualitatively.

All the meta-analyses used endpoint data or change from baseline to endpoint data instead of using the average of separate mean changes calculated at different intervals of time. All analyses were performed using SAS/STAT® software version 9.1 (SAS Institute Inc., Cary, NC), Statistical Package for the Social Sciences® for Windows® (SPSS® version 14.1, SPSS Inc., Chicago, IL), and RevMan version 4.1 (Cochrane Collaboration, Oxford, UK). A P-value of less than 0.05 was considered statistically significant. *A* 5-point change from baseline to endpoint was considered a clinically meaningful change [31].

## Results

**Figure 1:**
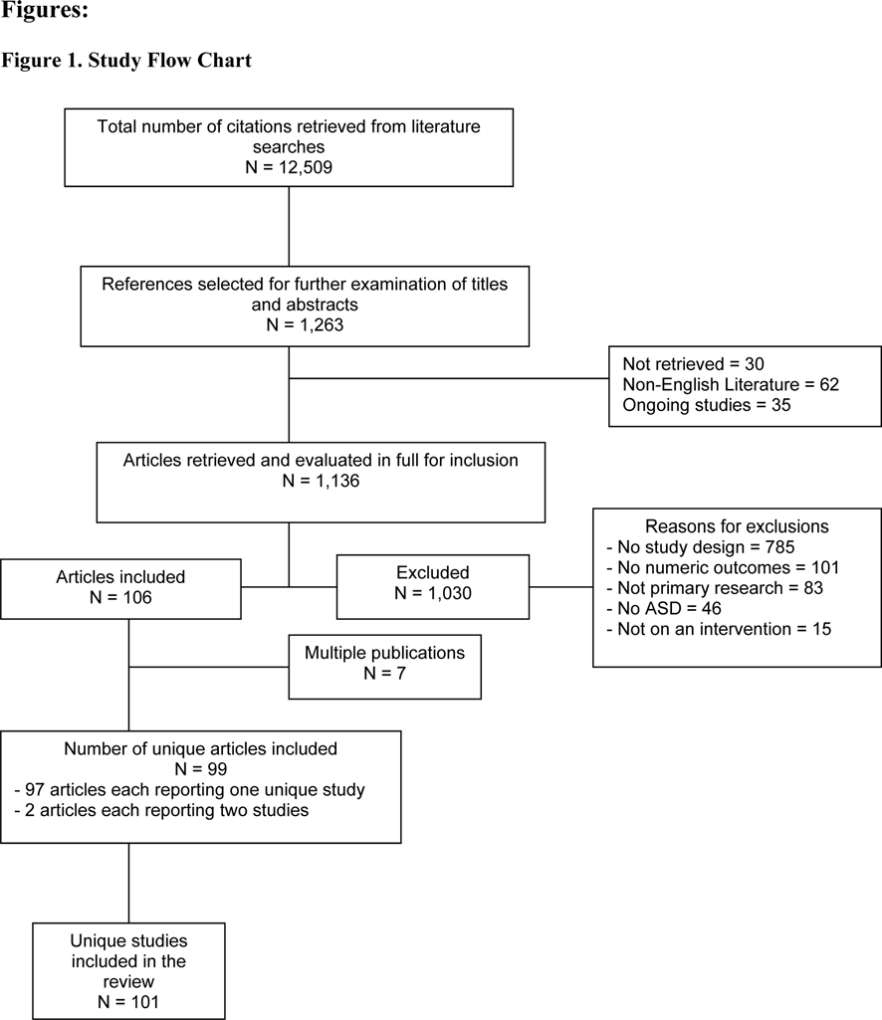
Study Flow Chart.

One hundred and one unique studies were included in the review. There were 55 RCTs, [32]–[83], 32 controlled clinical trials [84]–[115], four prospective cohort studies [116]–[119] and 10 retrospective cohort studies [120]–[129]. Figure 1 outlines the study flow for the review.

### Description of Studies

**Figure 2:**
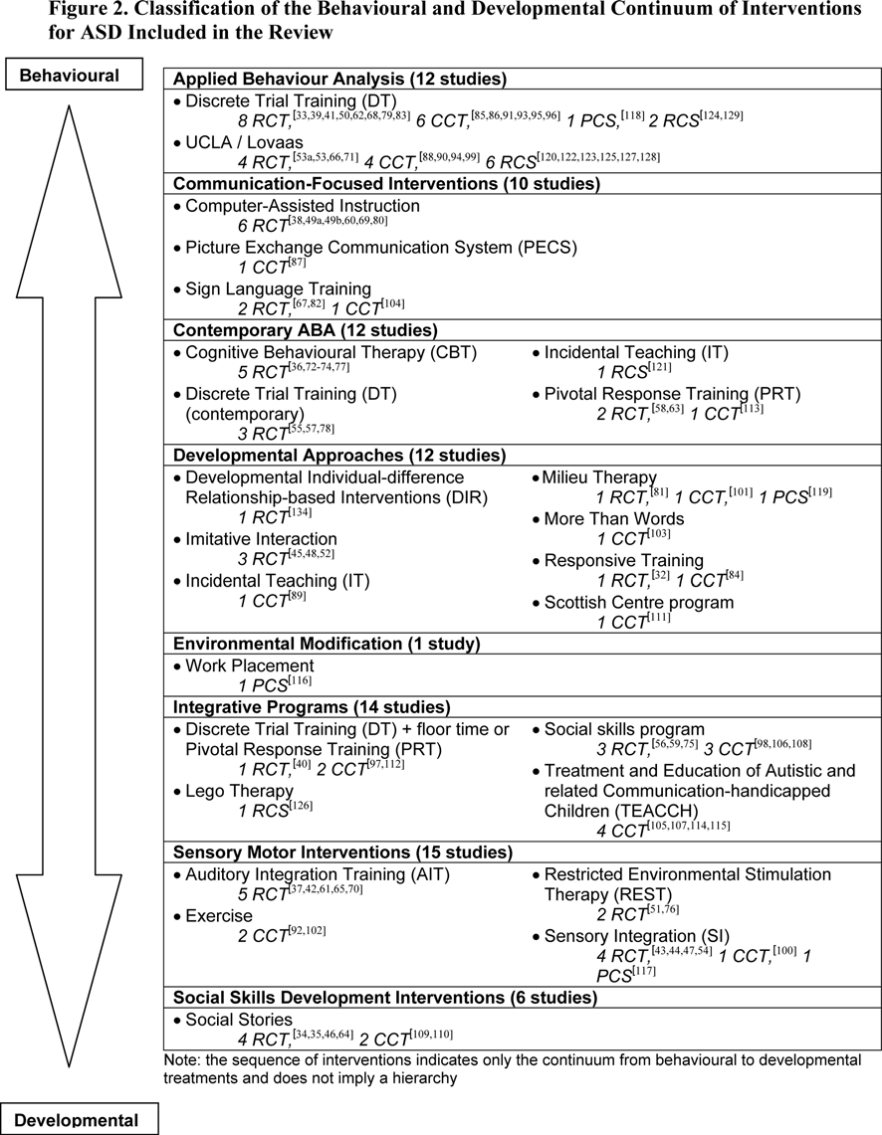
Classification of the Behavioural and Developmental Continuum of Interventions for ASD Included in the Review.

The studies evaluated the effect of eight broad types of interventions for ASD: Applied Behaviour Analysis (ABA) interventions, communication-focused interventions, contemporary ABA, developmental approaches, environmental modification programs, integrative programs, sensory motor interventions, and social skills development interventions (Figure 2). The studies were published between 1977 and 2007, with 2002 as the median year of publication. Data from a total of 2566 participants (median sample size = 22 per study; interquartile range: 15 to 36; n = 99) were reported in the studies. The median chronological age of participants in the studies was 62 months (interquartile range: 42 to 105 months; n = 84). Seventy-six percent of the studies included populations of infants or toddlers (less than 6 years of age), 44 per cent included school age children (6 to 12 years of age), 25 percent included adolescents (13 to 18 years of age), and only 11 percent included adults (older than 18 years of age). Studies included participants with conditions described as autistic disorder (93 percent), progressive developmental disorder (23 percent), Asperger’s syndrome (14 percent), high-functioning autism (5 percent), atypical autism (2 percent), not yet diagnosed autism (1 percent), and other (3 percent) such as autistic savant, or autistic-like conditions. The majority of the studies (67 percent) did not report on the level of severity of autistic symptoms in the study population. Participants with severe symptoms of ASD were included in 20 percent of the studies, whereas 19 percent included participants with moderate symptoms. Those with mild symptoms were not frequently included in the studies (15 percent). Summaries of the study characteristics and details of individual findings are presented in Table 1.

**Table 1:** Characteristics of the studies included in the review by type of intervention and study design.

Study	Study design, duration	Age, diagnosis	Intervention	Comparison group	Author’s conclusions
**APPLIED BEHAVIOUR ANALYSIS**					
**Discrete Trial Training**					
Andrews E, 1998[33] USA	RCT Parallel 25 days	Age NR Autistic disorder	DT N = 3	DT N = 3	1) An instructional model based on extra-stimulus prompts produced a significant improvement in measures of motor skills and learning ability.
Collier D, 1987[39] Canada	RCT Parallel NR	Age NR Autistic disorder	DT N = 3	DT N = 3	1) The extra-stimulus prompt group performed significantly better in bowling than the within-stimulus prompt group in terms of task analytic level achieved. 2) Groups did not differ in reinforcements or punishments.
Dugan KT, 2006[41] USA	RCT Parallel 5 days	58.9 mo Autistic disorder, PDD	DT N = 3	DT N = 2	1) There were no significant differences between the use of picture activity schedules and prompting at home or at school in the rate of on-task behaviours. 2) There were no significant differences in the number of on-schedule behaviours between the groups.
				SE N = 2	
Harris SL, 1982[50] USA	RCT Cross-over 7 wk	42.7 mo Autistic disorder	DT N = 4	WL N = 5	1) Behaviour modification training did not produce changes in parental speech oriented language. 2) There was no significant change in child speech after behaviour modification.
Nelson DL, 1980[62] USA	RCT Parallel NR	Age NR Autistic disorder	DT N = NR	DT N = NR	1) The use of color-coded extra prompts does not accelerate the development of generalizable skills in daily living activities of autistic children. 2) This technique is probably inefficient in teaching shoe-lacing to autistic children.
Sherman J, 1988[68] Canada	RCT Parallel 8 mo	62 mo Autistic disorder	DT N = 5	DT N = 5	1) Behavioural effects seem to favour the non-residential groups. 2) Non-residential groups demonstrated consistent improvements in functional behaviour.
				DT N = 5	
White SJ, 2000[79] USA	RCT Parallel 2 wk	Age NR Autistic disorder, Asperger's syndrome, PDD	DT N = 15	DT N = 15	1) Discrete trial therapy with negative feedback did not produce significant changes in the number of labels learned or the number of trials to reach criterion. 2) Discrete trial therapy with negative feedback produced a significant reduction of maladative behaviours.
Zifferblatt SM, 1977[83] USA	RCT Parallel 1 mo	Age NR Autistic disorder	DT N = NR	DT N = NR	1) There were no significant differences between home generalization and school generalization in establishing generalization.
Bernard-Opitz V, 2004[85] Singapore	CCT Cross-over 10 wk	38.8 mo Autistic disorder	DT N = 4	DIT N = 4	1) Behavioural and natural play interventions produced positive gains in play, attention, compliance, and communication. 2) Attendance and compliance was higher following the behavioural condition compared to play condition.
Birnbrauer JS, 1993[86] Australia	CCT Parallel 24 mo	36.9 mo Autistic disorder, PDD	DT N = 9	Control (ND) N = 5	1) Implementation of the Murdoch early intervention program can produce substantial gains in child functioning levels and parental stress in less than the ideal circumstances Lovaas described.
Elliott RO Jr, 1991[91] USA	CCT Parallel 8 wk	312 mo Autistic disorder	DT N = 11	DIT N = 12	1) Both analog language teaching and natural language teaching increased initial and long-term generalization and retention. 2) Natural language teaching is strongly suported as preferable for people with autism and MR.
Harris SL, 1990[93] USA	CCT Parallel NR	53.6 mo Autistic disorder	DT N = 5	LEAP N = 5	1) There were no significant differences in changes in language ability between the autistic children in the segregated and integrated class.
Howlin P, 1981[95] UK	CCT Parallel 6 mo	70.9 mo Autistic disorder	DT N = 16	NT N = 16	1) Participants in the home-based language training program made significantly greater improvements in functional use of speech. 2) There were no significant difference between groups on language level at followup.
				SC	
Hung DW, 1983[96] Canada	CCT Parallel 18 mo	110.6 mo Autistic disorder	DT N = 11	SE N = 6	1) A systems-based educational program significantly improved functional skills in autistic children. 2) A systems-based educational program is significantly less expensive than a residential program.
				SE N = 4	
				Residential N = 6	
Pechous EA, 2001[118] USA	PCS 6 mo	48.9 mo Autistic disorder	DT N = 7	NT N = 7	1) IBP significantly increased secured attachment behaviours and mothers’ sensitivity compared to no treatment.
Fenske EC, 1985[124] USA	RCS NR	75.1 mo Autistic disorder	DT N = 9	DT N = 9	1) There is a significant relationship between age at entry into a behavioural intervention program and positive treatment outcomes.
Tung R, 2005[129] USA	RCS NR	70.6 mo Autistic disorder, PDD, High-functioning autism	DT N = 3	NT N = 2	1) DT instruction produced less initiation or expansion of social contact with peers, but they responded more to interactions (non-verbal and verbal) with peers than children without DT instruction.
**UCLA/Lovaas**					
Hilton JC, 2005[53a] USA	RCT Parallel 6 wk	59 mo Autistic disorder	Lovaas N = 5	DIR N = 5	1) There were no significant differences between ABA and DIR intervention programs on measures of communication and symbolic behaviour.
Hilton JC, 2005[53b] USA	RCT Parallel 6 wk	65.5 mo Autistic disorder, PDD	Lovaas N = 5	DIR N = 5	1) Children receiving ABA demonstrated significant improvement for language comprehension than DIR. 2) There were no significant differences between ABA and DIR intervention programs on measures of communication and symbolic behaviour.
Sallows GO, 2005[66] USA	RCT Parallel 4 yr	33.6 mo Autistic disorder	Lovaas N = 13	Lovaas N = 10	1) The UCLA early intensive behavioural treatment can be implemented in a clinical setting. 2) Outcomes after 4 years of treatment (cognitive, language, adaptive, social, and academic measures), were similar for both groups.
Smith T, 2000[71] USA	RCT Parallel 7 yr	35.9 mo Autistic disorder, PDD	Lovaas N = 15	Lovaas N = 13	1) The intensive treatment group was significantly superior to the parents training group at producing improvements in IQ, visual-spacial skills and language development.
Cohen H, 2006[88] USA	CCT Parallel 3 yr	31.7 mo Autistic disorder, PDD	Lovaas N = 21	SE N = 21	1) There were no significant differences between the UCLA model implemented in a community setting and special education classes at local public schools in language comprehension and non-verbal skills. 2) EIBT can be successfully implemented in a community setting.
Eikeseth S, 2002[90], [166] Norway	CCT Parallel 12 mo	65.7 mo Autistic disorder	Lovaas N = 13	SE N = 12	1) Children in the behavioural group displayed significantly fewer disruptive behaviours than the eclectic group. 2) The behavioural group showed more gains than the eclectic group on IQ, language and adaptive behaviour.
Howard JS, 2005[94] USA	CCT Parallel ∼14 mo	33.6 mo Autistic disorder, PDD	Lovaas N = 29	SE N = 16	1) Learning rates at followup were substantially higher for children in the IBT group. 2) The IBT group had statistically higher mean standard scores in all skill domains than the control groups, except for motor skills.
Lovaas OI, 1987[99], [167] USA	CCT Parallel 30 mo	Age NR Autistic disorder	Lovaas N = 19	Lovaas N = 19	1) Participants in the intensive-long-term behaviour modification group obtained normal-range IQ scores and successful first grade performance in public schools.
Arnold CL, 2003[120] USA	RCS 9 yr	40.8 mo Autistic disorder, PDD, Dual diagnosis of MR	Lovaas N = 17	SE N = 16	1) Children in the ABA group did not make significant gain in cognitive ability but there was a trend of ABA improving and SE declining cognitive ability. 2) There were no significant differences between the treatment groups in intelligence measures, symptom severity and cognitive skills.
Eldevik S, 2006[122] Norway	RCS 2 yr	Age NR Autistic disorder	Lovaas N = 13	SC N = 15	1) Low-intensity behavioural treatment produced significant improvements in intellectual functioning, language comprehension, expressive language and communication skills when compared to an eclectic treatment group.
Farrell P, 2005[123] UK	RCS 2 yr	44.5 mo Autistic disorder	Lovaas N = 7	DT+TEACCH N = 7	1) Both ABA/Lovaas and LUFAP parents and staff were satisfied with the programs. 2) Both groups of children made considerable progress on socialization, daily living skills, communication and intelligence measures.
Hutchison-Harris J, 2004[125] USA	RCS 3 yr	39.1 mo Autistic disorder	Lovaas N = 44	Lovaas N = 35	1) Three years of intensive ABA intervention produced statistically significant improvements in language, cognitive ability, adaptive behaviour and overall pathology.
Sheinkopf SJ, 1998[127] USA	RCS 20 mo	34.6 mo Autistic disorder, PDD	Lovaas N = 9	RI N = 9	1) Participants in the home-based behavioural treatment obtained statistically significant higher IQ score at followup than the control. 2) There was a statistically significant reduction of symptoms severity after participating in the home-based behavioural treatment.
Smith T, 1997[128] USA	RCS ∼2–3 yr	37.0 mo PDD	Lovaas N = 11	Lovaas N = 10	1) Participants in the intensive behavioural treatment achieved higher IQ and had more expressive speech than the control. 2) Behavioural problems were reduced in both groups. 3) Intensively treated children achieved clinically meaningful gains relative to the comparison group but remained quite delayed.
**COMMUNICATION-FOCUSED INTERVENTIONS**					
**Computer-Assisted Instruction**					
Bolte S, 2006[38] Germany	RCT Parallel 5 wk	27.3 mo Autistic disorder	Computer-assisted instruction N = 3	NT N = 4	1) Facial affect recognition training did not produce a significant activation in the fusiform gyrus. 2) Facial affect recognition training produced some behavioural improvements.
Golan O, 2006[49a] UK	RCT Parallel 10–15 wk	30.7 mo Asperger's syndrome, High-functioning autism	Computer-assisted instruction N = 19	NT N = 22	1) Interactive multimedia produced improvement in emotion recognition and close, but not distant, generalization tasks. 2) No difference was found between the treatment groups on either feature- based or holistic tasks of distant generalization.
Golan O, 2006[49b] UK	RCT Parallel 10 wk	24.95 mo Asperger's syndrome, High-functioning autism	Computer-assisted instruction N = 13	Social skills program N = 13	1) Interactive multimedia significantly improved close generalization tasks. 2) The interactive multimedia intervention produced significant effects on verbal IQ.
Moore M, 2000[60] USA	RCT Parallel NR	Age NR Autistic disorder	Computer-assisted instruction N = NR	Computer-assisted instruction N = NR	1) Children in the computer group were significantly more attentive, more motivated and learned more vocabulary than those in the behavioural program.
Silver M, 2001[69] UK	RCT Parallel NR	172.2 mo Autistic disorder, Asperger's syndrome	Computer-assisted instruction N = 10	RI N = 11	1) The computer program group was significantly superior to the control group on improving childrens' ability to recognize and predict emotions in others.
Williams C, 2002[80] UK	RCT Cross-over 10 wk	56 mo Autistic disorder	Computer-assisted instruction N = 4	RI N = 4	1) The study was not long enough to show substantial improvement in reading skills. 2) Children spoke >2 times the number of words during the computer than book condition.
**Picture Exchange Communication System**					
Carr D, 2007[87], [133] UK	CCT Parallel 5 wk	68.0 mo Autistic disorder	PECS N = 24	RI N = 17	1) PECS produced a significant increase in communication initiations and dyadic interactions compared to the control.
**Sign Language Training**					
Saraydarian KA, 1994[67] USA	RCT Parallel 1 wk	Age NR Autistic disorder	Sign language training N = 10	NT N = 10	1) A controlled language training program produced an improvement in oral language and non-verbal communication in children with autism.
Yoder PJ, 1988[82], [168] USA	RCT Parallel NR	64.2 mo Autistic disorder	Sign language training N = 15	SLT N = 15	1) Speech alone, simultaneous presentation and alternating presentation conditions facilitated more child-initiated speech during treatment than the sign alone condition.
				Sign language training+LT-simultaneous N = 15	
				Sign language training+LT-alternation N = 15	
Oxman J, 1979[104] Canada	CCT Parallel 7 mo	111 mo Autistic disorder, Autistic-like	Sign language training N = 5	SLT N = 5	1) Simultaneous communication training produced some increase in the frequency of immediate vocal responding to speech models.
**CONTEMPORARY ABA**					
**Cognitive Behavioural Therapy**					
Berg HP, 2002[36] USA	RCT Parallel NR	98.5 mo Autistic disorder, Asperger's syndrome, PDD	CBT N = 10	Control (ND) N = 9	1) Training in cognitive perspecive-taking may remediate deficits in language and visual perspective-taking ability.
Sofronoff K, 2007[72] Australia	RCT Parallel 6 wk	129.4 mo Asperger’s syndrome	CBT N = 24	WL N = 21	1) The cognitive behavioural intervention produced a significant decrease in episodes of anger and an increase in parents’ confidence in managing their child’s anger.
Sofronoff K, 2005[73] Australia	RCT Parallel 6 wk	127.4 mo Asperger's syndrome	CBT N = 23	CBT N = 25	1) The two intervention groups demonstrated significant decreases in parent-reported anxiety symptoms at followup and a significant increase in the child’s ability to generate positive strategies in an anxiety-provoking situation.
				WL N = 23	
Sofronoff K, 2002[74], [169] Australia	RCT Parallel 4 wk	99 mo Asperger’s syndrome	CBT N = 32	CBT N = 36	1) Both parent management training interventions produced significant improvement in the number and intensity of problem behaviours and ratings of social skills. 2) There was significant effect in self-efficacy between the groups.
				WL N = 20	
Tonge B, 2006[77] Australia	RCT Parallel 20 wk	46.7 mo Autistic disorder	CBT N = 35	CBT N = 33	1) Both parent education & behaviour management intervention and the parent education & counseling interventions resulted in significant improvement in overall mental health. 2) Parent education & behaviour management alleviated a greater percentage of anxiety, insomnia and somatic symptoms than parent education & counselling.
				SC N = 35	
**Discrete Trial Training (contemporary)**					
Jocelyn LJ, 1998[55] Canada	RCT Parallel 12 wk	43.3 mo Autistic disorder, PDD	DT+IT N = 16	SC N = 19	1) The caregiver-based intervention program produced greater gains in language abilities, significant increases in caregivers' kwledge of autism, greater perception of control on the mothers' part and greater parent satisfaction. 2) Significant difference in autism symptoms was found between the groups.
Kasari C, 2006[57] USA	RCT Parallel 6 wk	42.6 mo Autistic disorder	DT+IT+PRT+Millieu Teaching N = 20	DT+IT+PRT+Millieu Teaching N = 21	1) The joint attention group showed a significant increase in initiation and responsiveness to joint attention and improvements in mother-child interactions. 2) Children in the play group showed more diverse types of symbolic play and higher play levels in interaction with their mothers. 3) There were differences between joint attention and play groups on initiating shows and coordinating joint looks.
				Control (ND) N = 17	
Wang P, 2005[78] China	RCT Parallel 5 wk	68.2 mo Autistic disorder	DT+IT N = 15	WL N = 12	1) Parents in the training group performed significantly better on a measure of kwledge of autism. 2) Parents in the training group scored significantly higher on responsiveness during free play interactions. 3) There were differences between the groups in parental stress levels.
**Incidental Teaching**					
Bloch J, 1980[121] USA	RCS 24 mo	47.7 mo Autistic disorder	IT N = 12	RI N = 14	1) An individualized language development program produced significant gains in language development after 1 year of treatment. 2) The program facilitated the gain of prelinguistic and linguistic skills.
**Pivotal Response Training**					
Koegel RL, 1996[58] USA	RCT Parallel NR	62.4 mo Autistic disorder	PRT N = 10	PRT N = 7	1) Families in the PRT condition showed more positive interactions and better communication style when compared with the ITB intervention. 2) The ITB training condition did not appear to have any significant impact on the parents' interactional style.
Openden DA, 2005[63] USA	RCT Parallel 4 days	61.0 mo Autistic disorder	PRT+DT N = 16	WL N = 16	1) PRT significantly increased the expression of positive affect, responsivity to opportunities for language and functional verbal utterances.
Stahmer AC, 2001[113] USA	CCT Parallel 12 wk	35.3 mo Autistic disorder	PRT N = 11	PRT N = 11	1) The addition of a parent education support group to a parent education program may increase parent mastery of teaching techniques and children's language skills.
**DEVELOPMENTAL APPROACHES**					
**Developmental Individual-difference Relationship-based Interventions**					
Gonzalez JS, 2006[134], [170] USA	RCT Parallel 8 wk	Age NR Autistic disorder	DIR N = 4	NT N = 4	1) Implementation of the DIR program did not produce significant changes in behavioural repetitive stereotypies when compared to a non-DIR program group. 2) There were significant changes in positive social interactions or negative social interaction skills between the groups.
**Imitative Interaction**					
Escalona A, 2002[45] USA	RCT Parallel NR	62.6 mo Autistic disorder	Imitative interaction N = 10	DCI N = 10	1) The contingency condition seemed to be more effective in facilitating a distal social behaviour (attention), while the imitative condition was more effective in facilitating a proximal social behaviour (touching).
Field T, 2001[48] USA	RCT Parallel NR	64.8 mo Autistic disorder	Imitative interaction N = NR	DCI N = NR	1) Repeated sessions of adult imitation increased both distal and proximal social behaviours. 2) Compared to contingently responsive group, imitation group showed significantly less time being inactive/playing alone and more time showing object behaviours.
Heimann M, 2006[52] Norway	RCT Parallel NR	77.2 mo Autistic disorder	Imitative interaction N = 10	DCI N = 10	1) An imitation interaction strategy produced a significant increase of both proximal and distal social behaviours compared to the contingency group. 2) The imitation intervention significantly increased children’s imitation skills at a more generalized level.
**Incidental Teaching**					
Eagle R, 2006[89] USA	CCT Cross-over NR	88.7 mo Autistic disorder, Atypical autism, PDD	IT N = 12	DIT N = 10	1) There were significant differences between the passive and social behaviour conditions on interpersonal distance and social initiation.
**Milieu Therapy**					
Yoder P, 2006[81], [171] USA	RCT Parallel 6 mo	33.6 mo Autistic disorder, PDD	Milieu therapy N = 17	PECS N = 19	1) PECS was more successful than RPMT in increasing the frequency of different non-imitative spoken communication acts and the number of different non-imitative words.
Macalpine ML, 1999[101] USA	CCT Parallel 8 mo	Age NR Autistic disorder	Milieu therapy N = 12	NT N = 6	1) The microdevelopmental method facilitated the development of higher cognitive abilities and successfully reversed the course of autism.
Wetherby AM, 2006[119] USA	PCS NR	25.1 mo Autistic disorder, PDD	Milieu therapy N = 17	Milieu therapy N = 18	1) This program produced significant improvements in communication measures compared to the control. 2) There were differences between the groups on communicative means and play behaviour.
**More Than Words**					
McConachie H, 2005[103] UK	CCT Parallel 7 mo	36.6 mo Autistic disorder, PDD, Not yet diagnosed autism	More than Words N = 26	WL N = 25	1) The More than Words program produced a significant advantage in parents' observed use of facilitative strategies and in childrens' vocabulary size. 2) There were significant differences between the groups on children's social score or behaviour, parental stress or adaptation.
**Responsive Training**					
Aldred C, 2004[32] UK	RCT Parallel 12 mo	49.5 mo Autistic disorder	Responsive Training N = 14	SC N = 14	1) A dyadic social communication treatment can improve autistic symptoms across severity and age groups in terms of quality of reciprocal social communication and expressive language.
Beckloff DR, 1997[84] USA	CCT Parallel 10 wk	75.6 mo Autistic disorder, Atypical autism, PDD, High-functioning autism	Responsive Training N = 12	NT N = 11	1) Filial therapy produced a non-significant but positive trend in parents' attitude toward autism, children's aggressive problems, externalizing problems and depressive or anxiety symptoms.
**Scottish Centre program**					
Salt J, 2002[111] Scotland	CCT Parallel 10 mo	41.0 mo Autistic disorder	Scottish Centre N = 12	WL N = 5	1) A developmentally-based early intervention program produced improvements on measures of joiunt attention, social interaction, imitation, daily living skills, motor skills and adaptive behaviour.
**ENVIRONMENTAL MODIFICATION**					
**Work Placement**					
Garcia-Villamisar D, 2007[116] Spain	PCS 30 mo	Age NR Autistic disorder	Work placement N = NR	WL N = NR	1) Supported employment produced a significantly greater improvement in non-vocational outcomes. 2) The intervention elicited positive changes in cognitive performance.
**INTEGRATIVE PROGRAMS**					
**Discrete Trial Training (DT) combinations**					
Drew A, 2002[40] UK	RCT Parallel 12 mo	22.5 mo Autistic disorder	DT+PRT+Millieu Teaching N = 12	SC N = 12	1) There was some evidence that the parent training group made more progress in language development. 2) The language ability of both groups remained severely compromised at followup. 3) Groups did t differ in non-verbal IQ, symptom severity or parental stress at followup.
Jelveh M, 2003[97] USA	CCT Parallel 5 mo	58.5 mo Autistic disorder	DT+Floor time N = 9	WL N = 5	1) Treatment groups showed improvements in social play and increased social behaviours.
Shade-Monuteaux DM, 2003[112] USA	CCT Cross-over 3 mo	25.2 mo Autistic disorder	DT+Floor time N = 23	NT N = 22	1) An integrated treatment approach is effective in improving social communication and joint attention skills in young children with ASD.
**Lego Therapy**					
Legoff DB, 2006[126] USA	RCS 3 yr	112.8 mo Autistic disorder, Asperger's syndrome, PDD	Lego Therapy N = 60	SC N = 57	1) Lego therapy produced significant gains on measures of social skills and autistic symptoms compared to the control group.
**Social skills program**					
Kalyva E, 2005[56] UK	RCT Parallel 2 mo	49.6 mo Autistic disorder	Social skills program N = 3	WL N = 2	1) Circle of friends can improve the communication and social skills of children with autism.
Lanquetot R, 1989[59] USA	RCT Parallel 4 wk	61.2 mo Autistic disorder	Social skills program N = 10	RI N = 10	1) Peer modeling produced a significant decrease in autistic, angry and aggressive behaviours in the treatment group.
Solomon M, 2004[75] USA	RCT Parallel 20 wk	112.4 mo Asperger’s syndrome, PDD, High-functioning autism	Social skills program N = 9	WL N = 9	1) The social adjustment group showed statistically significant improvements in facial expression recognition and problem solving.
Lopata C, 2006[98] USA	CCT Parallel 6 wk	120 mo Aperger's syndrome	Social skills program N = 9	Social skills program N = 12	1) There were significant differences between treatment and control groups in parent-rated and staff-rated social skills, adaptability and atypical behaviour.
Ozonoff S, 1995[106] USA	CCT Parallel 4.5 mo	164.5 mo Autistic disorder, PDD	Social skills program N = 5	NT N = 4	1) A social skills training program produced non-significant but considerable improvements on false belief tasks. 2) The intervention did not produce changes in parent and teacher ratings of social competence.
Provencal SL, 2003[108] USA	CCT Parallel 8 mo	171.6 mo Autistic disorder, Asperger's syndrome	Social skills program N = 10	SC N = 9	1) Participants receiving the social skills intervention did not demonstrate statistically significant improvements in social and behavioural functioning at home or school. 2) Marginal effectiveness reported for some symptoms.
**Treatment and Education of Autistic and related Communication-handicapped Children (TEACCH)**					
Ozonoff S, 1998[105] USA	CCT Parallel 4 mo	53.4 mo Autistic disorder	TEACCH N = 11	SC N = 11	1) A home program intervention improved significantly more in imitation, fine motor, gross motor, non-verbal conceptual skills and overall PEP-R scores. 2) The home program intervention was effective in enhancing development in young children with autism.
Panerai S, 2002[107] Italy	CCT Parallel 12 mo	111.1 mo Autistic disorder	TEACCH N = 8	RI N = 8	1) TEACCH program was more effective than the control 2) TEACCH produced significant changes in cognitive performance, developmental age, motor skills, daily living skills, play and leisure.
Tsang SKM, 2007[114] China	CCT Parallel 6 mo	48.7 mo Autistic disorder, PDD	TEACCH N = 18	SC N = 16	1) The TEACCH program produced a significantly greater improvement in measures of perception, fine motor, gross motor skills, social adaptive functioning and developmental abilities than the control.
Van Bourgondien ME, 2003[115] USA	CCT Parallel 6 mo	25.6 mo Autistic disorder	TEACCH N = 6	Family homes N = 8	1) Participants in the TEACCH program experienced a higher quality of treatment as compared to participant in control settings. 2) TEACCH participants had a significant increase in communication, socialization, developmental planning and positive behaviour management.
				Group homes N = 11	
				Institutions N = 5	
**SENSORY MOTOR INTERVENTIONS**					
**Auditory Integration Training**					
Bettison S, 1996[37] Australia	RCT Parallel 1 mo	Range = 3–17 yr Autistic disorder, Asperger's syndrome	AIT N = 40	Unmodified music N = 40	1) The AIT and control groups showed similar improvement in behaviour, severity of autism and verbal and performance IQ. 2) Both AIT and listening to unmodified music may have a beneficial effect on children with autism.
Edelson SM, 1999[42] USA	RCT Parallel 3 mo	139.0 mo Autistic disorder	AIT N = 9	Unmodified music N = 9	1) AIT showed significant improvements in auditory P300 ERP and behavioural problems.
Mudford OC, 2000[61] UK	RCT Cross-over 3 mo	113 mo Autistic disorder	AIT N = 7	Unmodified music N = 9	1) The control condition was superior than AIT on parent-rated hyperactivity and direct observation measures of ear-occlusion. 2) There were differences in intelligence measures and language comprehension between groups at followup.
Rimland B, 1995[65] USA	RCT Parallel 3 mo	123 mo Autistic disorder	AIT N = 9	Unmodified music N = 9	1) AIT produced statistically significant improvements in adaptive behaviour. 2) AIT did not decrease sound sensitivity in children with autism.
Smith DE, 1985[70] USA	RCT Parallel 20 wk	106 mo Autistic disorder	AIT N = 7	NT N = 7	1) AIT produced an increase in attentiveness, appropriate behaviour, communication, signing and less stereotypies.
**Exercise**					
Greer-Paglia K, 2006[92] USA	CCT Cross-over 30 wk	89.2 mo Autistic disorder	Exercise N = 29	Social stories N = 25	1) Creative dance produced significantly greater social gains than in circle time condition for both verbal and non-verbal children. 2) The performance gap between verbal and non-verbal autistic children was smaller in the creative dance group than circle time group.
Mason MA, 2005[102] USA	CCT Parallel 10 wk	Age NR Autistic disorder, Asperger's syndrome, PDD, Autistic savant	Exercise N = 56	SC N = 33	1) Therapeutic horse riding produced a significant increase in communication and social skills.
**Restricted Environmental Stimulation Therapy**					
Harrison JR, 1991[51] USA	RCT Parallel 8 wk	149.2 mo Autistic disorder	REST N = 6	Ward placement N = 6	1) There were significant differences between REST and the control group in measures of stress, intelligence, vocal behaviour and autistic symptoms.
Suedfeld P, 1983[76] Canada	RCT Cross-over 6 wk	Age NR Autistic disorder	REST N = 5	Ward placement N = 3	1) REST produced statistical significant positive changes in learning, social and play behaviour and cognitive functioning.
**Sensory Integration**					
Edelson SM, 1999[43] USA	RCT Parallel 6 wk	91.0 mo Autistic disorder	Sensory integration N = 5	Placebo N = 7	1) There was a significant reduction in tension and a marginally significant reduction in anxiety for children who received deep pressure compared to children who did not. 2) Deep pressure may have a calming effect for persons with autism, particularly those with high levels of arousal or anxiety.
Escalona A, 2001[44] USA	RCT Parallel 1 mo	62.5 mo Autistic disorder	Sensory integration N = 10	Reading N = 10	1) Massage therapy provided by parents appears to be an effective way of diminishing sleep problems, stereotypies and off-task behaviour.
Field T, 1997[47] USA	RCT Parallel 4 wk	54 mo Autistic disorder	Sensory integration N = 11	Unstructured play N = 11	1) Both the touch therapy and the touch control groups improved in measures of touch aversion and off-task behaviour. 2) The touch therapy group significantly decreased stereotypic behaviours compared to the touch control group.
Jarusiewicz B, 2002[54] USA	RCT Parallel 6–8 mo	84 mo Autistic disorder	Sensory integration N = 12	WL N = 12	1) Neuro-feeback training produced significant improvements in autism symptoms and behaviour.
Luce JB, 2003[100] USA	CCT Parallel 6 mo	68 mo Autistic disorder	Sensory integration N = 12	NT N = 12	1) There was difference between groups in the proportion of time children were enganged in stereotypic behaviours.
Hartshorn K, 2001[117] USA	PCS 2 mo	Age NR Autistic disorder	Sensory integration N = 38	Control (ND) N = 38	1) Movement therapy led to a significant increase in attentive behaviours and a decrease in stress behaviours and touch aversion.
**SOCIAL SKILLS DEVELOPMENT INTERVENTIONS**					
**Social Stories**					
Andrews SM, 2005[34] USA	RCT Parallel 1 day	120.6 mo Autistic disorder	Social Stories N = 10	Regular story N = 10	1) The social story was effective in increasing game playing skills, story comprehension and generalized social comprehension.
Bader R, 2006[35] USA	RCT Cross-over 3 days	105.2 mo Autistic disorder	Social Stories N = 10	Regular story N = 10	1) Social stories succesfully increased facial emotion learning and labeling for children with autism.
Feinberg MJ, 2002[46] USA	RCT Parallel 1 wk	122.5 mo Autistic disorder	Social Stories N = 17	Regular story N = 17	1) Social stories is an effective intervention to teach social skills to children with autism.
Quirmbach LM, 2006[64] USA	RCT Parallel 1 wk	114.7 mo Autistic disorder	Social Stories N = 15	Social Stories N = 15	1) Social stories using either a standard or a directive approach produced significantly higher game play skills than the control group. 2) Standard and directive groups did t significantly differ on rate of play skill improvement.
				Control (ND)	
Ricciardelli D, 2006[109] USA	CCT Parallel 10 mo	134.0 mo Autistic disorder	Social Stories N = 3	RI N = 3	1) Social stories combined with social skills curriculum did not produce significantly different changes in measures of social skills. 2) There was a significant difference in maintaining attention, favouring the treatment group.
Romano J, 2002[110,110] USA	CCT Parallel 6 wk	Age NR Autistic disorder	Social Stories N = 5	NT N = 5	1) Social stories significantly decreased aggressive behaviour and improved communication and socialization skills.

ABA = applied behaviour analysis; AIT = auditory integration training; ASD = autism spectrum disorders; CBT = cognitive behaviour therapy; CCT = controlled clinical trial; CFI = communication-focused intervention; DCI = developmental contingency interaction; DIT = developmental incidental teaching; DIR = developmental individual-difference relationship-based intervention; DT = discrete trial training; EM = environmental modification; ERP = event-related potential; IBT = intensive behaviour analytic treatment; IQ = Intellectual quotient; IT = incidental teaching; LEAP = learning experiences, an alternative program for preschoolers and their parents; LT = language therapy; LUFAP = Lancashire under fives autism programme; M = number of males; mo =  month(s); MR = mental retardation; ND = not described; NR = not reported; NT = no treatment; OCD = obsessive compulsive disorder; PCS = prospective cohort study; PDD = pervasive developmental disorder; PECS = picture exchange communication system; PRT = pivotal response training; RCS = retrospective cohort study; RCT = randomized controlled trial; REST = restricted environmental stimulation therapy; RI = regular instruction; RPMT = responsive education and prelinguistic milieu teaching; SC = standard care; SE = special education ; SLT = speech language therapy; TEACCH = treatment and education of autistic and related communication-handicapped children; UCLA = University of California, Los Angeles wk = week(s); WL = wait-list; yr = year(s).

### Quality of Studies

Details on the methodological quality of the studies are presented in Table 2 and Table 3. Briefly, the majority of trials (83 percent) failed to mention how representative the sample was in terms of the study setting, the selection criteria for enrolling participants, and the operational definition of ASD. A minority of studies (32 percent) reported on monitoring the fidelity of intervention implementation. Although more than half of the trials (64 percent) reported the use of randomization, few trials (seven trials) reported the procedure for separating the process of randomization from the recruitment of participants. The majority of trials (89 percent) failed to clearly report how they concealed the sequence of allocation to the interventions under study. Less than half of the studies (43 percent) reported that blind or independent outcome assessment was conducted. In terms of attrition bias, 33 percent of the trials provided a description of withdrawals and dropouts from the study. Finally, just over half of the trials (54 percent) reported their sources of funding. Thirty-two percent were funded by government agencies, 22 percent received funding from foundations or societies, 19 percent used internal funds, and five percent were funded by private industry.

**Table 2:** Methodological quality of RCTs and CCTs.

Study	Intervention	QUALITY DOMAINS						
		Method for sequence generation	Description of selection criteria	Description of therapeutic regimen	Blinding of outcome assessment	ITT analysis	Prior estimate of sample size	Funding reported
		Allocation concealment	Withdrawals per group reported	Description of treatment provider	Assessment of treatment fidelity	Testing randomization	Report of measures of precision	
Andrews E, 1998[33] RCT	ABA/DT	Unclear	Partial	Partial	Unclear	No	Inadequate	No
		Unclear	No	Inadequate	Yes	Inadequate	Inadequate	
Collier D, 1987[39] RCT	ABA/DT	Unclear	Partial	Partial	Unclear	No	Inadequate	No
		Unclear	No	Adequate	Yes	Inadequate	Inadequate	
Dugan KT, 2006[41] RCT	ABA/DT	Unclear	Adequate	Adequate	Unclear	No	Inadequate	No
		Unclear	No	Partial	Unclear	Adequate	Inadequate	
Harris SL, 1982[50] RCT	ABA/DT	Inappropriate	Partial	Adequate	Yes	No	Inadequate	Yes
		Unclear	Yes	Adequate	Unclear	Adequate	Inadequate	
Nelson DL, 1980[62] RCT	ABA/DT	Unclear	Partial	Adequate	No	No	Inadequate	Yes
		Unclear	No	Partial	Unclear	Partial	Partial	
Sherman J, 1988[68] RCT	ABA/DT	Unclear	Partial	Partial	Unclear	No	Inadequate	Yes
		Unclear	No	Inadequate	Unclear	Inadequate	Inadequate	
White SJ, 2000[79] RCT	ABA/DT	Unclear	Inadequate	Adequate	Unclear	No	Inadequate	No
		Unclear	No	Adequate	Yes	Inadequate	Partial	
Zifferblatt SM, 1977[83] RCT	ABA/DT	Unclear	Inadequate	Adequate	Unclear	No	Inadequate	No
		Unclear	No	Partial	Unclear	Inadequate	Inadequate	
Bernard-Opitz V, 2004[85] CCT	ABA/DT	Unclear	Partial	Adequate	Yes	No	Inadequate	Yes
		Unclear	No	Adequate	Yes	Partial	Inadequate	
Birnbrauer JS, 1993[86] CCT	ABA/DT	Unclear	Adequate	Adequate	Yes	No	Inadequate	Yes
		Unclear	Yes	Partial	Yes	Adequate	Inadequate	
Elliott RO Jr, 1991[91] CCT	ABA/DT	Unclear	Inadequate	Partial	Yes	No	Inadequate	No
		Unclear	No	Partial	Yes	Partial	Inadequate	
Harris SL, 1990[93] CCT	ABA/DT	Unclear	Inadequate	Inadequate	Unclear	No	Inadequate	No
		Unclear	No	Partial	Unclear	Partial	Inadequate	
Howlin P, 1981[95] CCT	ABA/DT	Unclear	Inadequate	Inadequate	Unclear	No	Inadequate	No
		Unclear	No	NA	Unclear	Adequate	Partial	
Hung DW, 1983[96] CCT	ABA/DT	Unclear	Partial	Adequate	Unclear	No	Inadequate	Yes
		Unclear	No	Adequate	Yes	Inadequate	Partial	
Hilton JC, 2005[53a] RCT	ABA/Lovaas	Unclear	Inadequate	Adequate	Unclear	No	Inadequate	Yes
		Unclear	Yes	Adequate	Yes	Partial	Partial	
Hilton JC, 2005[53b] RCT	ABA/Lovaas	Unclear	Inadequate	Adequate	Unclear	No	Inadequate	Yes
		Unclear	Yes	Adequate	Yes	Partial	Partial	
Sallows GO, 2005[66] RCT	ABA/Lovaas	Unclear	Adequate	Adequate	Yes	No	Inadequate	Yes
		Appropriate	No	Adequate	Yes	Adequate	Partial	
Smith T, 2000[71] RCT	ABA/Lovaas	Appropriate	Adequate	Adequate	Yes	No	Inadequate	Yes
		Appropriate	No	Adequate	Yes	Adequate	Partial	
Cohen H, 2006[88] CCT	ABA/Lovaas	Unclear	Partial	Adequate	Yes	No	Inadequate	Yes
		Unclear	Yes	Adequate	Yes	Adequate	Adequate	
Eikeseth S, 2002[90] CCT	ABA/Lovaas	Unclear	Adequate	Adequate	Yes	No	Inadequate	Yes
		Unclear	Yes	Adequate	Unclear	Partial	Adequate	
Howard JS, 2005[94] CCT	ABA/Lovaas	Unclear	Partial	Adequate	Yes	No	Inadequate	Yes
		Unclear	Yes	Adequate	No	Adequate	Partial	
Lovaas OI, 1987[99] CCT	ABA/Lovaas	Unclear	Partial	Adequate	Yes	No	Inadequate	Yes
		Unclear	Yes	Partial	Unclear	Adequate	Partial	
Bolte S, 2006[38] RCT	Communication focused/Computer-assisted instruction	Unclear	Partial	Adequate	Unclear	No	Inadequate	No
		Unclear	Yes	Partial	Unclear	Inadequate	Partial	
Golan O, 2006[49a] RCT	Communication focused/Computer-assisted instruction	Unclear	Partial	Adequate	Yes	No	Inadequate	Yes
		Unclear	No	Inadequate	Unclear	Adequate	Partial	
Golan O, 2006 [49b] RCT	Communication focused/Computer-assisted instruction	Unclear	Partial	Adequate	Yes	No	Inadequate	Yes
		Unclear	No	Inadequate	Unclear	Adequate	Partial	
Moore M, 2000[60] RCT	Communication focused/Computer-assisted instruction	Unclear	Inadequate	Partial	Unclear	No	Inadequate	No
		Unclear	No	Inadequate	Unclear	Inadequate	Inadequate	
Silver M, 2001[69] RCT	Communication focused/Computer-assisted instruction	Unclear	Inadequate	Adequate	Unclear	No	Inadequate	No
		Unclear	Yes	Inadequate	Unclear	Adequate	Partial	
Williams C, 2002[80] RCT	Communication focused/Computer-assisted instruction	Unclear	Partial	Adequate	No	No	Inadequate	Yes
		Unclear	No	Partial	Unclear	Partial	Inadequate	
Carr D, 2007[87] CCT	Communication focused/PECS	Unclear	Partial	Adequate	No	No	Inadequate	Yes
		Unclear	No	Partial	Unclear	Partial	Inadequate	
Saraydarian KA, 1994[67] RCT	Communication focused/Sign language training	Unclear	Partial	Adequate	No	No	Inadequate	No
		Unclear	Yes	Inadequate	Yes	Adequate	Partial	
Yoder PJ, 1988[82] RCT	Communication focused/Sign language training	Unclear	Partial	Adequate	Unclear	No	Inadequate	Yes
		Unclear	No	Partial	Yes	Adequate	Partial	
Oxman J, 1979[104] CCT	Communication focused/Sign language training	Unclear	Inadequate	Inadequate	Unclear	No	Inadequate	Yes
		Unclear	No	Inadequate	Unclear	Inadequate	Inadequate	
Berg HP, 2002[36] RCT	Contemporary ABA/CBT	Unclear	Partial	Adequate	Unclear	No	Inadequate	No
		Unclear	No	Adequate	Unclear	Inadequate	Partial	
Sofronoff K, 2007[72] RCT	Contemporary ABA/CBT	Unclear	Adequate	Adequate	Unclear	No	Inadequate	Yes
		Unclear	Yes	Partial	Yes	Adequate	Partial	
Sofronoff K, 200573 RCT	Contemporary ABA/CBT	Unclear	Adequate	Adequate	Yes	No	Inadequate	No
		Unclear	Yes	Partial	Yes	Adequate	Partial	
Sofronoff K, 2002[74] RCT	Contemporary ABA/CBT	Unclear	Partial	Adequate	No	Yes	Inadequate	Yes
		Unclear	No	Partial	Unclear	Inadequate	Partial	
Tonge B, 2006[77] RCT	Contemporary ABA/CBT	Appropriate	Partial	Adequate	Yes	No	Inadequate	No
		Unclear	Yes	Partial	Yes	Adequate	Partial	
Jocelyn LJ, 1998[55] RCT	Contemporary ABA/DT+IT	Appropriate	Adequate	Partial	Yes	No	Inadequate	Yes
		Appropriate	Yes	Partial	Unclear	Adequate	Partial	
Kasari C, 2006[57] RCT	Contemporary ABA/DT+IT+PRT+Millieu Teaching	Unclear	Adequate	Partial	Yes	No	Inadequate	Yes
		Unclear	Yes	Adequate	Yes	Adequate	Partial	
Wang P, 2005[78] RCT	Contemporary ABA/DT+IT	Unclear	Partial	Partial	No	No	Inadequate	No
		Unclear	Yes	Inadequate	Unclear	Adequate	Partial	
Koegel RL, 1996[58] RCT	Contemporary ABA/PRT	Unclear	Inadequate	Partial	Yes	No	Inadequate	Yes
		Unclear	No	NA	Yes	Adequate	Partial	
Openden DA, 2005[63] RCT	Contemporary ABA/PRT+DT	Unclear	Partial	Adequate	Yes	No	Inadequate	No
		Unclear	Yes	Adequate	Yes	Adequate	Partial	
Stahmer AC, 2001[113] CCT	Contemporary ABA/PRT	Unclear	Inadequate	Adequate	Yes	No	Inadequate	Yes
		Unclear	No	Inadequate	Yes	Partial	Partial	
Gonzalez JS, 2006[134] RCT	Developmental/DIR	Unclear	Inadequate	Adequate	Unclear	No	Inadequate	No
		Unclear	No	Inadequate	Yes	Inadequate	Partial	
Escalona A, 2002[45] RCT	Developmental/Imitative interaction	Unclear	Partial	Partial	Unclear	No	Inadequate	Yes
		Unclear	No	Inadequate	Unclear	Inadequate	Inadequate	
Field T, 2001[48] RCT	Developmental/Imitative interaction	Unclear	Inadequate	Partial	Unclear	No	Inadequate	Yes
		Unclear	No	Inadequate	Unclear	Inadequate	Partial	
Heimann M, 2006[52] RCT	Developmental/Imitative interaction	Unclear	Inadequate	Adequate	No	No	Inadequate	Yes
		Unclear	No	Inadequate	Unclear	Partial	Partial	
Eagle R, 2006[89] CCT	Developmental/Incidental Teaching	Unclear	Inadequate	Adequate	Unclear	No	Inadequate	No
		Unclear	No	Partial	Unclear	Adequate	Partial	
Yoder P, 2006[81] RCT	Developmental/Milieu therapy	Appropriate	Partial	Adequate	Yes	No	Inadequate	Yes
		Appropriate	Yes	Partial	Yes	Adequate	Partial	
Macalpine ML, 1999[101] CCT	Developmental/Milieu therapy	Unclear	Inadequate	Adequate	Unclear	No	Inadequate	No
		Unclear	No	Inadequate	Unclear	Inadequate	Inadequate	
McConachie H, 2005[103] CCT	Developmental/More than Words	Unclear	Adequate	Partial	No	No	Inadequate	Yes
		Unclear	No	Inadequate	Yes	Adequate	Adequate	
Aldred C, 2004[32] RCT	Developmental/Resposive Training	Unclear	Adequate	Partial	Yes	Yes	Inadequate	Yes
		Appropriate	Yes	NA	Unclear	Adequate	Partial	
Beckloff DR, 1997[84] CCT	Developmental/Resposive Training	Unclear	Partial	Adequate	Unclear	No	Inadequate	No
		Unclear	Yes	Adequate	Unclear	Inadequate	Partial	
Salt J, 2002[111] CCT	Developmental/Scottish Centre	Unclear	Inadequate	Inadequate	Yes	No	Inadequate	No
		Unclear	Yes	Inadequate	Unclear	Adequate	Partial	
Drew A, 2002[40] RCT	Integrative Programs/DT+PVT+Millieu Teaching	Appropriate	Inadequate	Adequate	Unclear	Yes	Inadequate	Yes
		Unclear	No	Partial	Unclear	Adequate	Partial	
Jelveh M, 2003[97] CCT	Integrative Programs/DT+Floor time	Unclear	Partial	Adequate	No	No	Inadequate	Yes
		Unclear	Yes	Partial	Unclear	Partial	Inadequate	
Shade-Monuteaux DM, 2003[112] CCT	Integrative Programs/DT+Floor time	Unclear	Inadequate	Inadequate	Unclear	No	Inadequate	No
		Inappropriate	No	Inadequate	Unclear	Adequate	Inadequate	
Kalyva E, 2005[56] RCT	Integrative Programs/Social skills program	Unclear	Inadequate	Adequate	Unclear	No	Inadequate	No
		Unclear	No	Partial	Unclear	Partial	Partial	
Lanquetot R, 1989[59] RCT	Integrative Programs/Social skills program	Appropriate	Partial	Inadequate	No	No	Inadequate	No
		Appropriate	No	Inadequate	Unclear	Partial	Inadequate	
Solomon M, 2004[75] RCT	Integrative Programs/Social skills program	Unclear	Adequate	Adequate	Unclear	No	Inadequate	Yes
		Unclear	No	Partial	Unclear	Adequate	Partial	
Lopata C, 2006[98] CCT	Integrative Programs/Social skills program	Inappropriate	Inadequate	Adequate	No	No	Inadequate	No
		Unclear	Yes	Partial	Yes	Inadequate	Partial	
Ozonoff S, 1995[106] CCT	Integrative Programs/Social skills program	Unclear	Partial	Partial	Yes	No	Inadequate	Yes
		Unclear	No	Inadequate	Unclear	Adequate	Partial	
Provencal SL, 2003[108] CCT	Integrative Programs/Social skills program	Unclear	Adequate	Adequate	Unclear	No	Inadequate	No
		Unclear	Yes	Partial	Yes	Adequate	Inadequate	
Ozonoff S, 1998[105] CCT	Integrative Programs/TEACCH	Unclear	Inadequate	Partial	Unclear	No	Inadequate	No
		Unclear	No	Partial	Unclear	Adequate	Partial	
Panerai S, 2002[107] CCT	Integrative Programs/TEACCH	Unclear	Partial	Partial	Yes	No	Inadequate	No
		Unclear	No	Partial	Unclear	Adequate	Inadequate	
Tsang SKM, 2007[114] CCT	Integrative Programs/TEACCH	Unclear	Partial	Partial	Unclear	No	Inadequate	No
		Unclear	Yes	Inadequate	Unclear	Adequate	Partial	
Van Bourgondien ME, 2003[115] CCT	Integrative Programs/TEACCH	Unclear	Inadequate	Inadequate	No	No	Inadequate	Yes
		Unclear	No	Inadequate	Unclear	Adequate	Partial	
Bettison S, 1996[37] RCT	Sensory Motor/AIT	Unclear	Inadequate	Adequate	Yes	No	Adequate	Yes
		Appropriate	No	Inadequate	Unclear	Inadequate	Partial	
Edelson SM, 1999[42] RCT	Sensory Motor/AIT	Unclear	Inadequate	Adequate	Yes	No	Inadequate	Yes
		Unclear	No	Inadequate	Unclear	Inadequate	Inadequate	
Mudford OC, 2000[61] RCT	Sensory Motor/AIT	Unclear	Inadequate	Adequate	Yes	No	Inadequate	Yes
		Unclear	No	Adequate	Unclear	Inadequate	Partial	
Rimland B, 1995[65] RCT	Sensory Motor/AIT	Unclear	Inadequate	Partial	Yes	No	Inadequate	Yes
		Unclear	Yes	Inadequate	Unclear	Adequate	Inadequate	
Smith DE, 1985[70] RCT	Sensory Motor/AIT	Unclear	Inadequate	Partial	Yes	No	Inadequate	Yes
		Unclear	No	Partial	Yes	Inadequate	Partial	
Greer-Paglia K, 2006[92] CCT	Sensory Motor/Exercise	Unclear	Adequate	Adequate	Yes	No	Inadequate	No
		Unclear	No	Adequate	Unclear	Adequate	Inadequate	
Mason MA, 2005[102] CCT	Sensory Motor/Exercise	Unclear	Inadequate	Adequate	Unclear	No	Inadequate	No
		Unclear	No	Partial	Unclear	Adequate	Partial	
Harrison JR, 1991[51] RCT	Sensory Motor/Rest	Unclear	Partial	Partial	Yes	No	Inadequate	Yes
		Unclear	Yes	Inadequate	Unclear	Inadequate	Partial	
Suedfeld P, 1983[76] RCT	Sensory Motor/Rest	Unclear	Inadequate	Adequate	Yes	No	Inadequate	Yes
		Unclear	No	NA	Unclear	Inadequate	Inadequate	
Edelson SM, 1999[43] RCT	Sensory Motor/SI	Unclear	Inadequate	Partial	No	No	Inadequate	Yes
		Unclear	No	Inadequate	Unclear	Inadequate	Inadequate	
Escalona A, 2001[44] RCT	Sensory Motor/SI	Unclear	Inadequate	Adequate	Yes	No	Inadequate	Yes
		Unclear	No	Partial	Unclear	Inadequate	Inadequate	
Field T, 1997[47] RCT	Sensory Motor/SI	Unclear	Inadequate	Adequate	Yes	No	Inadequate	Yes
		Unclear	No	Partial	Unclear	Inadequate	Inadequate	
Jarusiewicz B, 2002[54] RCT	Sensory Motor/SI	Unclear	Inadequate	Adequate	Unclear	No	Inadequate	No
		Unclear	Yes	Inadequate	Unclear	Adequate	Inadequate	
Luce JB, 2003[100] CCT	Sensory Motor/SI	Unclear	Inadequate	Partial	Yes	No	Inadequate	No
		Unclear	No	Partial	Unclear	Inadequate	Partial	
Andrews SM, 2005[34] RCT	Social skills development/Social Stories	Appropriate	Inadequate	Partial	Yes	No	Inadequate	No
		Unclear	No	Inadequate	Yes	Adequate	Partial	
Bader R, 2006[35] RCT	Social skills development/Social Stories	Unclear	Adequate	Partial	Unclear	No	Inadequate	No
		Unclear	No	Inadequate	Unclear	Partial	Partial	
Feinberg MJ, 2002[46] RCT	Social skills development/Social Stories	Unclear	Partial	Partial	No	No	Inadequate	No
		Unclear	No	Inadequate	Unclear	Adequate	Partial	
Quirmbach LM, 2006[64] RCT	Social skills development/Social Stories	Inappropriate	Partial	Adequate	Yes	No	Inadequate	No
		Inappropriate	No	Partial	Unclear	Adequate	Partial	
Ricciardelli D, 2006[109] CCT	Social skills development/Social Stories	Unclear	Inadequate	Adequate	Unclear	No	Inadequate	No
		Unclear	No	Partial	Unclear	Partial	Partial	
Romano J, 2002[110] CCT	Social skills development/Social Stories	Unclear	Partial	Adequate	No	No	Inadequate	No
		Unclear	No	Partial	Unclear	Inadequate	Partial	

ABA = applied behavioural analysis; AIT = auditory integration training; DT = discrete trial; IT = incidental teaching; NA = not applicable; ND = not described; PCS = prospective cohort study; PECS = picture exchange communication system; RCS = retrospective cohort study; SI = sensory integration.

**Table 3:** Methodological quality of observational studies.

Study	Intervention Type	QUALITY DOMAINS					Funding
		Report of selection criteria	Report of therapeutic regimen	Measure of exposure assessment reliable	Main potential confounders incorporated in design/analysis	Report of measures of precision	
		Representa-tiveness of exposed cohort	Report of treatment provider	Outcome assessment blind to exposure status	Important differences between groups other than exposure to intervention	Report of how potential confounders were distributed	
				Method of outcome assessment valid and reliable			
Pechous EA, 2001[118] PCS	ABA/DT	Partial	Inadequate	Partial	Partial	Partial	No
		Somewhat	Inadequate	ND	No	No	
				Yes			
Fenske EC, 1985[124] RCS	ABA/DT	Inadequate	Adequate	Yes	Partial	Inadequate	No
		Somewhat	Inadequate	ND	Yes	Yes	
				ND			
Tung R, 2005[129] RCS	ABA/DT	Partial	Partial	Partial	No	Inadequate	No
		Yes	Inadequate	ND	No	Yes	
				Yes			
Arnold CL, 2003[120] RCS	ABA/Lovaas	Partial	Partial	Partial	No	Partial	No
		Somewhat	Partial	ND	Yes	Yes	
				Yes			
Eldevik S, 2006[122] RCS	ABA/Lovaas	Partial	Adequate	Yes	No	Partial	Yes
		Somewhat	Adequate	Yes	No	Yes	
				Yes			
Farrell P, 2005[123] RCS	ABA/Lovaas	Inadequate	Partial	ND	No	Inadequate	No
		Somewhat	Partial	ND	Yes	No	
				Yes			
Hutchison-Harris J, 2004[125] RCS	ABA/Lovaas	Adequate	Adequate	Yes	Yes	Partial	No
		Somewhat	Adequate	Yes	No	Yes	
				Yes			
Sheinkopf SJ, 1998[127] RCS	ABA/Lovaas	Inadequate	Adequate	Yes	Yes	Partial	No
		ND	Partial	Yes	No	Yes	
				Yes			
Smith T, 1997[128] RCS	ABA/Lovaas	Adequate	Adequate	Yes	Partial	Partial	No
		ND	Adequate	Yes	Yes	No	
				Yes			
Bloch J, 1980[121] RCS	Contemporary ABA/IT	Inadequate	Inadequate	Yes	No	Inadequate	No
		Somewhat	Partial	No	Yes	No	
				No			
Wetherby AM, 2006[119] PCS	Developmental/Milieu therapy	Partial	Adequate	Yes	No	Partial	Yes
		Yes	Adequate	Yes	Yes	Yes	
				Yes			
Garcia-Villamisar D, 2007[116] PCS	Environmental Modification/Work placement	Partial	Partial	ND	No	Partial	Yes
		Yes	Inadequate	ND	No	Yes	
				ND			
Legoff DB, 2006[126] PCS	Integrative Programs/Lego Therapy	Inadequate	Inadequate	Yes	Yes	Partial	No
		Somewhat	Inadequate	Yes	No	Yes	
				Yes			
Hartshorn K, 2001[117] PCS	Sensory Motor/SI	Inadequate	Partial	ND	Yes	Partial	Yes
		ND	Partial	Yes	No	No	
				Yes			

ABA = applied behavioural analysis; DT = discrete trial; IT = incidental teaching; ND = not described; PCS = prospective cohort study; RCS = retrospective cohort study; SI = sensory integration.

Overall, the methodological quality of the 14 cohort studies was modest. In general, the cohort studies failed to protect against selection bias: only three studies clearly mentioned how representative the overall sample was in terms of the study setting, the description of the selection criteria, and the operational definition of ASD used for the study. The control for detection bias affecting the ascertainment of both exposure and outcome was moderate in the cohort studies. None of the studies used secure methods for ascertainment of exposure. The majority of the studies provided evidence on the reliability of methods for outcome assessment; however, only half of the studies explicitly stated that outcome assessment was blind to exposure status. Finally, only four observational studies disclosed their source of funding. The methodological strengths and weaknesses of individual studies, presented in Table 2 and Table 3, should be taken into consideration when interpreting the study results and conclusions.

### Summary of Findings

#### Applied Behaviour Analysis

Evidence from 31 studies (12 trials and 9 cohort studies) involving a total of 770 participants was analyzed on the use of discrete trial training and Lovaas therapy for ASD. The effects of discrete trial learning are inconsistent across studies. All the studies that compared discrete trial training to no treatment reported statistically significant findings [95], [118], [129]. Motor and functional outcomes more often demonstrated positive results compared to speech-related outcomes which were generally negative. All cohort studies demonstrated significant results [118], [124], [129]. Lovaas therapy was consistently found superior to standard care [99], [122] or regular instruction [94], [127] in terms of intellectual functioning, language comprehension, and communication skills. Generally, high-intensity Lovaas was found to be superior to low-intensity Lovaas in terms of intellectual functioning, communication skills, adaptive behaviour and overall pathology [71], [99], [125], [128]. The results for Lovaas therapy compared to special education showed variable results at the individual study level and seemed to indicate more effect for the medium-term (12 and 14 months, respectively) [90], [94] which was not apparent within the longer-term studies (3 and 9 years, respectively) [88], [120]. No significant differences were found within studies comparing Lovaas to Developmental Individual-difference relationship-based intervention (DIR) [53] or Integrative/Discrete trial combined with Treatment and Education of Autistic and related Communication Handicapped Children (TEACCH) [123]. Seven of the eight studies that reported significant findings for Lovaas therapy were non-RCTs [90], [94], [99], [122], [125], [127], [128]. Three of the four RCTs in this category reported no significant findings [53a,53b,66]. This observation has serious implications for the interpretation of evidence from non-RCTs. There is some evidence that results of RCTs and non-RCTs sometimes, but not always, differ,[130] and that non-RCT can be more prone to bias and overestimate treatment effects [131], [132].

#### Communication-focused Interventions

Ten trials involving 269 participants were identified that evaluated the effects of communication-focused interventions. Positive effects and statistically significant results were produced at the study level for emotional recognition [49], [69], close generalization tasks [49], verbal IQ [49], attention [60] and motivation [60]; these studies were all RCTs and had varied control groups including no treatment, as well as active interventions. There is evidence from three trials (2 RCT, 1 CCT) that sign language training provides benefits in terms of communication-related outcomes, such as articulation competence, oral language, nonverbal communication, and child-initiated speech [67], [82], [104]. There is also some suggestion that sign language training may be most effective when combined with other modalities [82]. One CCT of Picture Exchange Communication System versus regular instruction showed a significant increase in communication initiations and dyadic interactions [133].

#### Contemporary ABA

Evidence on the effects of contemporary ABA was identified from 12 studies (11 trials and one cohort study) with a total of 573 participants. At the individual study level, significant improvements in the child’s behaviour management, social skills, and parent’s mental health have been reported following cognitive behaviour therapy [72], [73], [77]. There is limited and inconclusive evidence for various combinations of discrete trial training, incidental teaching, pivotal response training, and milieu teaching [55], [57], [78], [121], and some evidence that pivotal response training may be beneficial for communication and social interaction [58], [63], [113].

#### Developmental Interventions

Twelve studies (11 trials and one cohort study) with a total of 256 participants were identified that evaluated the effects of interventions involving the application of developmental principles. Distinct modalities were evaluated within this category (e.g., imitative interaction, milieu therapy, responsive training, DIR, More than Words, and the Scottish Centre for Autism Preschool Treatment program). The available evidence appears to indicate no short-term differences between DIR and a no treatment group in aggression, self-stimulating behaviour, and social skills [134]. No evidence of effect was reported for the comparisons between two incidental teaching-based approaches in social interaction [89], two milieu-based approaches in communication and play behaviour [119], and responsive training versus no treatment in parents’ attitude toward ASD, children’s aggressive problems, externalizing problems, and depressive or anxiety symptoms [84]. Positive results were reported for the comparisons between milieu therapy and no treatment in cognitive abilities and course of ASD [101]; and for milieu therapy versus Picture Exchange Communication System in communication and play behaviour [81]. The comparison of More than Words to a wait-list control showed positive results in facilitative strategies and vocabulary size, however, no significant differences were observed for social skills, behaviour, or parental stress or adaptation [103]. Response training was superior to standard care in quality of reciprocal social communication and expressive language [32]. Finally, one small trial evaluated the Scottish Centre program versus a wait-list control and demonstrated positive results in joint attention, social interaction, imitation, daily living skills, motor skills, and adaptive behaviour [111].

#### Environmental Modification

One cohort study involving 44 participants examined work placement versus waitlist [116]. This prospective cohort study reported positive results in terms of significantly greater improvement in nonvocational outcomes and cognitive performance.

#### Integrative Programs

Evidence on the effects of a variety of integrative programs was obtained from 14 studies (13 trials and one cohort study) containing a total of 382 participants. Interventions in this category included Lego therapy, social skills program, and TEACCH. Lego therapy was evaluated in one large retrospective cohort study, and produced significant improvements in terms of social skills and autistic symptoms [126]. Six studies evaluating social skills programs produced inconsistent findings; there were no identifiable patterns in the outcomes examined [56], [59], [75], [98], [106], [108]. Individual studies that evaluated TEACCH consistently reported significant findings for a variety of outcomes, including but not limited to fine motor and gross motor skills, cognitive performance, social adaptive functioning, and communication [105], [107], [114], [115].

#### Sensory Motor Interventions

Fifteen studies (14 trials and one cohort study) were identified that evaluated the effects of interventions that involved the application of sensory motor principles, involving a total of 156 participants. Several modalities were evaluated within this category: auditory integration training, exercise, restricted environmental stimulation therapy, and sensory integration. Six studies of sensory integration versus no treatment groups reported statistically significant results for stereotypic behaviours, off-task behaviours, and touch aversion [43], [44], [47], [54], [100], [117], but it is not known how sensory integration compares to other active interventions. The results for communication-related outcomes are contradictory, and no effect was reported for intellectual functioning. Two relatively large studies on creative dance [92] and horse riding [102], respectively, demonstrated significant social gains. Studies on the effects of restricted environmental stimulation therapy provided inconclusive evidence [51], [76]. No studies evaluated effects over the long-term; therefore the sustainability of these changes is unknown.

#### Social Skills Development Intervention

Six trials, containing a total of 135 participants, provided evidence on the effects of social skills development interventions, all of which evaluated the effects of Social Stories^TM^ in ASD. Five of the six studies showed statistically significant results for a variety of outcomes related to social interaction at short-term (e.g., 1 day to 6 weeks) [34], [35], [46], [64], [110]. There were no studies comparing Social Stories^TM^ to other active treatments.

### Meta-analyses

A limited number of meta-analyses were feasible due to variations among the studies in the type of interventions assessed, the comparison groups, and the outcomes of interest. Of the 101 studies included in the review, 13 studies (six RCTs, five CCTs and two observational studies) contributed data to the meta-analysis. Table 4 summarizes the comparisons and outcomes that were suitable for meta-analysis. In a meta-analysis of three CCTs [38], [124], [127] involving 112 participants, statistically significant results were obtained for Lovaas treatment compared to special education on measures of adaptive behaviour (WMD = 11.8; 95% CI, 6.94 to 16.67), communication and interaction (WMD = 16.63; 95% CI, 11.25 to 22.01), comprehensive language (WMD = 12.84; 95% CI, 6.38 to 19.30), daily living skills (WMD = 5.61; 95% CI, 0.54 to 10.67), expressive language (WMD = 15.05; 95% CI, 6.19 to 23.90), overall intellectual functioning (SMD = 0.95; 95% CI, 0.44 to 1.46), and socialization (WMD = 9.17; 95% CI, 2.16 to 16.19). High-intensity Lovaas was shown to be superior to Low-intensity Lovaas on measures of intellectual functioning in two retrospective cohort studies with a total of 173 participants (SMD = 0.92; 95% CI, 0.61 to 1.24) [40], [111]. Pooling of two RCTs [18], [135] including 40 participants yielded statistically significant results for developmental approaches based on initiative interaction compared to contingency interaction in the amount of time spent in stereotyped behaviours (WMD = −0.40; 95% CI, −0.73 to −0.07), and the amount of time spent in distal social behaviour (WMD = 2.85; 95% CI, 0.99 to 4.71), but the effect sizes were not clinically significant. Statistically non-significant results were obtained for the comparisons between Lovaas and special education in measures of non-verbal intellectual functioning (three CCTs [38], [124], [127], N = 111 participants; SMD = 7.83; 95% CI, −2.86 to 18.52), Lovaas versus DIR on measures of communication skills (two RCTs [29a,29b], N = 18; SMD = 0.73; 95% CI, −0.26 to 1.72), computer assisted instruction versus no treatment on measures of facial expression recognition (two RCTs [136], [137], N = 48; SMD = 0.53; 95% CI, −0.05 to 1.12); and TEACCH versus standard care on measures of imitation skills (two CCTs [57], [121], N = 56; SMD of 0.46; 95% CI, −0.07 to 0.99), and eye-hand integration (two CCT [57], [121], N = 56s; SMD = −0.24; 95% CI, −0.77 to 0.28).

**Table 4:** Summary of the meta-analyses of the effects of behavioural and developmental interventions for ASD.

Comparison	Outcome	Studies and number of participants	Statistical method, effect size, heterogeneity	Statistical significance and direction of effect	Clinical significance
High-intensity versus low-intensity Lovaas	Intellectual functioning	2 RCS; [125], [128] N = 173	SMD = 0.92; 95% CI, 0.61 to 1.24 I^2^ = 0%	Yes. In favour of high-intensity Lovaas	Yes
Lovaas versus special education	Overall intellectual functioning	3 CCTs; [88], [90], [94] N = 112	SMD = 0.95; 95% CI, 0.44 to 1.46 I^2^ = 36.2%	Yes. In favour of Lovaas	Yes
	Adaptive behaviour	3 CCTs; [88], [90], [94] N = 112	WMD = 11.8; 95% CI, 6.94 to 16.67) I^2^ = 0%	Yes. In favour of Lovaas	Yes
	Communication and interaction	3 CCTs; [88], [90], [94] N = 111	WMD = 16.63; 95% CI, 11.25 to 22.01 I^2^ = 0%	Yes. In favour of Lovaas	Yes
	Comprehensive language	3 CCTs; [88], [90], [94] N = 112	WMD = 12.84 (95% CI, 6.38 to 19.30) I^2^ = 0%	Yes. In favour of Lovaas	Yes
	Expressive language	3 CCTs; [88], [90], [94] N = 111	WMD = 15.05 (95% CI, 6.19 to 23.90) I^2^ = 0%	Yes. In favour of Lovaas	Yes
	Daily living skills	3 CCTs; [88], [90], [94] N = 111	WMD = 5.61 (95% CI, 0.54 to 10.67) I^2^ = 0%	Yes. In favour of Lovaas	No
	Socialization	3 CCTs; [88], [90], [94] N = 112	WMD = 9.17 (95% CI, 2.16 to 16.19) I^2^ = 35.3%	Yes. In favour of Lovaas	Borderline
	Non-verbal intellectual functioning	3 CCTs; [88], [90], [94] N = 111	SMD = 7.83 (95% CI, −2.86 to 18.52) I^2^ = 38.1%	No	No
Lovaas versus DIR	Communication skills	2 RCTs;[53a,53b] N = 18	SMD = 0.73 (95% CI, −0.26 to 1.72) I^2^ = 0%	No	No
Computer assisted instruction versus NT	Facial expression recognition	2 RCTs; [38], [49b] N = 48	SMD = 0.53 (95% CI, −0.05 to 1.12) I^2^ = 0%	No	No
Imitative interaction versus contingency interaction approaches	Time spent in stereotyped behaviour	2 RCTs; [45], [48] N = 40	WMD = −0.40 (95% CI, −0.73 to −0.07) I^2^ = 0%	Yes. In favour of imitative interaction approach	No
	Time spent in distal social behaviour	2 RCTs; [45], [48] N = 40	WMD = 2.85 (95% CI, 0.99 to 4.71) I^2^ = 21.0%	Yes. In favour of imitative interaction approach	No
TEACCH versus standard care	Imitation skills	2 CCTs; [105], [114] N = 56	SMD of 0.46 (95% CI, −0.07 to 0.99) I^2^ = 0%	No	No
	Eye-hand integration	2 CCTs; [105], [114] N = 56	SMD = −0.24 (95% CI, −0.77 to 0.28) I^2^ = 0%	No	No

CCT = controlled clinical trials; DIR = Developmental Individual-difference relationship-based intervention; NT = no treatment; RCS = retrospective cohort studies; RCT = randomized clinical trail; SMD = standardized mean difference; WMD = weighted mean difference

Because of the very small number of trials available for each comparison, the effect of publication bias on the meta-analyses presented above was not analyzed.

## Discussion

Our systematic review of the indexed scientific literature on the effects of behavioural and developmental interventions for ASD has demonstrated a lack of agreement across the studies on the effect that these interventions may have on clinically relevant outcomes. Despite evidence, there is no clear answer regarding the most effective therapy to improve symptoms associated with ASD.

The interpretation and generalization of results summarized from individual studies is complicated by a number of factors. First, ASD is a complex diagnosis that represents a spectrum of symptoms. The varied interventions may target different symptoms or the same symptoms to different extents. As a result, practitioners and decision-makers may need to target their choice of treatment to the uniqueness of each presenting child and the symptoms that are most important for the well-being of each child and their family. In interpreting the literature, the reader needs to consider the findings in light of the study population and the outcomes that were evaluated. There is considerable potential for heterogeneity in the population, intervention, comparator and outcomes of interest, as ASD is a spectrum disorder, therapy is not always reported in detail, comparators are difficult to control for, and outcomes are somewhat subjective. It should be noted that controversy exists regarding the use of intellectual functioning as an outcome, since higher IQ scores may represent true increases or merely a better ability to take the test following the intervention [138]. Second, the interventions themselves are complex and multifaceted. Many of them have components that may be implemented in different ways, different settings, and by different people including both professionals and lay people. In some cases, this prohibits generalizations regarding a specific intervention. Third, consideration needs to be given to the comparison groups. As a general finding, it appears that any intervention is better than nothing. That is, most of individual studies showed benefits when an active intervention was compared to no treatment or wait-list controls. It has been reported that behavioural researchers sometimes include a comparison group that monitors symptoms for a period of time equivalent to the time required for the intervention in the active group before beginning the treatment [139]. Although this approach controls for certain non-specific treatment effects such as regression to the mean, the situation may create a negative expectation of improvement (i.e., no one expects to improve while they wait for treatment) and therefore, artificially inflate the difference between the active and the control (wait-list or no treatment) groups. Therefore, results of studies that compared active versus no treatment or wait-list controls should be interpreted with caution. Fourth, variation in results may occur due to different length of follow-up across studies. The length of follow-up must be appropriate to the nature of the intervention and its mechanism of action (i.e., how long an intervention is required to begin to have an effect) and the outcomes being measured (i.e., length of time to elicit change in a specific outcome). Further, consideration needs to be made for whether any observed changes are maintained over the longer-term. Finally, the results need to be considered in light of the methodological quality of the studies and their potential for bias (i.e., under or overestimation of treatment effects). A particular concern is related to the potential of outcome reporting bias in these studies, in which statistically significant results have a higher chance of being fully reported compared to non-significant results [140]. Although the presence of selective outcome reporting was not formally evaluated in our review, future evaluations of the evidence on the effectiveness of behavioural and developmental interventions should compare trial publications to protocols to verify whether changes or omissions in selected outcomes were introduced from registration to publication of the trial.

A few studies of modest methodological quality were available for meta-analysis, mostly reporting non-significant results. Few statistically significant results favoured Lovaas therapy and developmental approaches based on initiative interaction. The positive results from these meta-analyses need to be interpreted with caution, since biases, such as expectancy bias, cannot be excluded. It is unknown whether the non-significant results obtained for computer assisted instruction, and TEACCH are truly “negative findings” (i.e., evidence of no effect) or if there is a lack of power to detect a statistically significant result due to the low number of studies included in the meta-analyses (i.e., no evidence of effect). Finally, we found that 54 percent of the studies disclosed the source of funding, with most of the research being sponsored by government agencies or scientific societies. Only five percent of the studies declared private industry funding. There is evidence that industry funding of biomedical research may bias conclusions toward positive results for their products (sponsorship bias) [141]; however, there is no evidence on whether sponsorship bias extends beyond industry to other sources of funding.

### Applied Behaviour Analysis

Evidence was analyzed on the use of discrete trial training and Lovaas therapy for ASD. The evidence seems to provide some support for discrete trial training in terms of motor and functional skills but not for communication skills. Lovaas’ therapy showed benefits when compared to “no treatment” and evidence from meta-analysis of retrospective cohort studies showed greater effects for High versus Low intensity Lovaas. Results from a meta-analysis of CCTs demonstrated that Lovaas is superior to special education for a variety of outcomes, however, there is no definitive evidence suggesting superiority of Lovaas over other active interventions.

A previous review [142] concluded that overall, studies of behaviour analytic early intervention programs report substantial improvements, but the nature of improvements vary considerably across studies. The authors also recognize the methodological flaws in the earlier studies that preclude drawing definite conclusions related to programming. Other reviews [143]–[148] have also reported on the effects of early and intensive behavioural interventions for ASD. They agree that the majority of recent primary studies of reasonable quality document some improvement associated with behavioural intervention, but it remains to be determined if any one early and/or intensive intervention program is more effective than another. Furthermore, there was insufficient evidence to establish a relationship between the amount (per day and total duration) of any form of treatment program to obtain desirable outcomes. Replication in RCTs is needed to substantiate the use of Lovaas intervention and to determine the effect of treatment intensity on the outcomes of children with ASD.

### Communication-focused Interventions

Individual studies reported positive effects in motivation, IQ changes, and emotional recognition associated with communication-focused interventions; however the meta-analysis results were not statistically significant for the comparison between computer assisted instruction and no treatment on measures of facial expression recognition. Two previous reviews [149], [150] have examined the evidence from a variety of study designs of interventions that enhance communicative competence of individuals with ASD, such as assistive technology, augmentative and alternative communication methods. In keeping with these reviews, we conclude that future research is needed to better delineate the extent to which these interventions actually enhance outcomes in individuals with ASD.

### Contemporary ABA

The evidence supporting the use of contemporary ABA approaches is variable and there is no evidence to suggest that one approach is more effective than another. A previous meta-analysis [151] that included a variety of study designs other than RCTs, CCTs and observational cohort studies indicated that the contemporary ABA approach produces greater gains in cognitive skills than Lovaas or developmental approaches, but both contemporary ABA and Lovaas methods were similarly effective in fostering language and adaptive skills in this population. This remains a question for future research to confirm as the methodological difficulties with the primary studies have made it difficult to be conclusive.

### Developmental Interventions

Overall conclusions for developmental interventions are elusive due to the varied nature of the modalities, discrepant results across modalities, and limited evidence for each. Another review [151] has reported that compared to Lovaas and Contemporary ABA approaches, developmental interventions were found to be ineffective in the cognitive development of the participants but were effective in language development.

### Environmental Modification

Only one study assessed the effectiveness of interventions labelled under this category. General conclusions for this type of intervention are prohibited due to limited evidence.

### Integrative Programs

The evidence to support the use of these interventions is limited or inconsistent across studies. Another review [142] found similar results and emphasized the need to evaluate which components of these multi-faceted interventions are responsible for changes in clinically relevant outcomes.

### Sensory Motor Interventions

The evidence is either limited or inconsistent for this group of interventions to support their use in clinical practice. Interpretation of evidence for individual studies within this category needs to be considered in light of the comparison group, the length of follow-up, and the outcomes examined. One systematic review [152] has evaluated the efficacy of sensory and motor interventions for children with ASD. This review included a variety of study designs such as descriptive case studies, single-subject designs, and RCTs. The review concluded that many of the sensory and motor intervention approaches have shown mixed effects at short term for children with ASD through uncontrolled, descriptive studies. Previous reviews that have evaluated the evidence for various types of sensory and motor interventions have similarly reported mixed clinical effects and have concluded that there is insufficient evidence to support use of these interventions at present [11].

### Social Skills Development Intervention

The limited evidence supports Social Stories^TM^ for short-term improvement of social symptoms associated with ASD among school-aged children. Past reviews have examined the effect of Social Stories^TM^ [153], [154] in children and young adults with ASD [155]. The reviews conclude that the effects of Social Stories^TM^ are highly variable, and empirical foundation regarding its effectiveness is limited. However, the reviews agreed that published research in Social Stories^TM^ has demonstrated positive effects, and therefore provides preliminary support to consider it a promising intervention.

### Review Strengths and Limitations

A range of therapeutic approaches currently exists to help alleviate the symptoms of ASD. Due to the lack of a unique classification system to describe the variety of treatments for ASD, an intervention taxonomy system based on previous studies and experts’ opinion was developed for the purposes of the review. The categories considered seem to be sensible, but it may not be possible to find a framework that would mandate exactly this set. A potential limitation of this approach is that the therapies examined were pragmatically classified and other therapies such as music therapy, drama therapy, and animal therapy could have been included if other classification approaches had been used. Synthesis of the evidence for other therapeutic approaches not evaluated in our review are available in the scientific literature [156].

Our search strategy is likely to have identified the majority of the available literature on the efficacy and effectiveness of behavioural and developmental interventions for ASD. We particularly targeted the indexed literature, yet we also searched for theses and dissertations, which altogether represent almost one-third of the studies included in this review. However, we acknowledge the possibility that the review may not be fully comprehensive, as we did not include additional grey literature sources in our search strategy. It has been reported previously that on average, published trials show a 9 percent greater treatment effect than grey trials [157], [158] and therefore, there is the potential that our meta-analyses report an overestimate of the treatment effect. Further, the search results were initially screened by only one reviewer due to resource limitation. To date, there is no empirical evidence that indicates what the impact of screening by two, as opposed to one, reviewer has on selection bias; however, use of two reviewers during the screening process may have provided additional reassurance of the selection process.

We adopted a comprehensive strategy to appraise the methodological quality of the included studies. Our approach to quality focused mainly on an assessment of the internal validity of the studies, as recommended by several researchers [22], [159]; however, some aspects related to the external validity and adherence to the interventions under study were also considered. One of the limitations of this review is the restriction of included studies to English-language publications. We did not include foreign language literature because of the difficulties in translation. Particularly, there is a wealth of Japanese literature available that could prove very interesting. We do not know the magnitude of bias that the exclusion of foreign literature may have produced in the results of our meta-analysis [160], [161]. An additional limitation of our review is that studies were included regardless of whether there is evidence to support the psychometric properties of their outcome measures. Therefore, our analysis includes both instruments which are well validated for measuring clinical change in ASD (e.g., Wechsler Intelligence Scale for Children–Revised), as well as those that are commonly used, yet whose psychometric properties have not been studied in the ASD populations (e.g., Reynell Developmental Language Scales) [90].

### Clinical Relevance

The research reviewed in this examination of behavioural and developmental treatments for ASD reveals that there are a number of treatment programs, some comprehensive and others with a specific behavioural focus, that have been developed to treat the core symptoms of ASD. Across any one specific intervention approach, the research is lacking scientific rigour, replications are sparse, and outcomes are variable; however, there are some implications for practice. First, it appears that most children with ASD make at least some progress on desired outcomes during their participation in intervention programs. Yet, the progress that individuals make across these programs or treatment approaches varies; some show remarkable progress while others show slow or minimal gains. Further the sustainability of changes over time is unknown. The source of this variation is uncertain given the quality of the research to date. It is unclear how participant characteristics interact with specific treatment programs or specific components of programs. Thus, practitioners need to be mindful to communicate these uncertainties to families seeking intervention services. Second, when selecting a program, practitioners need to select programs that have at least some evidence of support, select programs that are manualized and ensure that interventionists are able to maintain the level and quality of implementation of the program. Manualized programs serve to provide standardization of an intervention, yet uniformity must be balanced with the need to individualize the intervention [14]. The interventions themselves are complex and multifaceted. The variation in the expression of the symptoms of ASD make individualization necessary yet this presents problems for clearly specifying and evaluating the essential components of any given intervention. McMahon has recommended manuals with “constrained flexibility”, where limited variation in the implementation of an intervention is permitted [162]. It is important to highlight that, although the evidence regarding the efficacy and effectiveness of behavioural and developmental interventions for ASD is currently limited, it does not mean that there is evidence of no effect from the interventions. The findings of this review are consistent with current clinical practice guidelines, which list various treatment options and approaches, yet offer limited guidance regarding choice of intervention [25], [163], [164]. However, guidelines do provide some parameters regarding which components constitute effective treatment programs, including: daily opportunities to use and increase spontaneous communication, engaging in meaningful learning activities that are functional in multiple settings, ongoing monitoring progress and adjustment of teaching practices to maximize progress, frequent interaction with typical peers and involvement of family members [164]. Until more reliable evidence is available, practitioners and decision-makers may need to target their choice of treatment to the uniqueness of each presenting child and the symptoms that are most important for the well-being of each child and their family.

### Future Research

Based on our review of the literature, there are several recommendations for future research on interventions for individuals with ASD. It is important that investigators make an effort to clearly define and report the procedures for the intervention under scrutiny. Researchers should consider the use of standard care as a comparison group (i.e., the treatment that is normally or optimally provided to people with a given condition). In order to allow for comparisons across studies, researchers should use standardized and validated outcome measures so that reporting on the effect of the interventions in terms of changes in core symptoms of ASD is more consistent. The impact of behavioural and developmental interventions upon family outcomes (e.g., functioning, quality of life, and finances) and their possible negative effects should also be further explored.

Studies on the effectiveness of behavioural and developmental interventions for ASD should continue to make improvements to meet accepted methodological standards for clinical research including: the use of randomization and allocation concealment, the implementation of intervention protocols that capture a wide range of skills and symptoms, blinded outcome assessment, assessing treatment fidelity, and implementing longitudinal designs with sufficient follow-up to evaluate treatment effects. One of the limitations of the existing literature is the small samples within individual studies. This limits generalizability and also raises questions around the interpretation of negative findings: whether such findings were due to inadequate sample size or true lack of effectiveness of the intervention. Better reporting of how the studies were planned, conducted and analyzed is required. Established guidelines to this end, such as the Consolidated Standards of Reporting Trials for reporting trials of behavioural interventions and Strengthening the Reporting of Observational Studies in Epidemiology guidelines, should be followed [165].

Programs can vary in terms of the degree of prescription versus flexibility of the intended approach, the extent to which adult control is necessary in fostering children’s development of social, communicative and other abilities, the degree of social and natural context of the intervention, the focus on adult versus child centered procedures, the exposure to more natural interactions and learning opportunities, the role of typical peers, and how the major goals of treatment are prioritized. The success of any approach will depend on the needs of the individual, which vary greatly. Rigorous scientific evaluation of the evidence is necessary to estimate the likely benefits of any particular approach. Studies that assist in determining whether an individual is being helped by a particular therapy might be extremely helpful by sparing the burden of participation if no benefits are identified facilitating a switch to other types of intervention.

### Conclusions

The most effective behavioural and developmental treatments for ASD should include interventions that address the behavioural, social, and communication deficits associated with the disorder. Intervention studies suffer from methodological problems that preclude definitive conclusions regarding their efficacy. This systematic review tried to elucidate a question regarding the effects of behavioural and developmental approaches to ASD and drew conclusions as to the potential effects of these interventions based on the results of clinical trials and observational cohort studies. Without better operational definitions of the critical components of interventions, consistency in choice and reporting of outcome measures, and enhanced descriptions of participant heterogeneity, we will see few gains in understanding ‘best practices.’

While this review suggests that Lovaas may improve some core symptoms of ASD compared to special education, these findings are based on pooling outcomes from a few, methodologically weak studies with few participants and relatively short-term follow-up. As no definitive behavioural or developmental intervention improves all symptoms for all individuals with ASD, it is recommended that clinical management be guided by individual needs and availability of resources. Future studies on the effectiveness of these interventions need to be more rigorous. Further, the evidence needs to be interpreted in light of the study populations, characteristics and application of the interventions, outcomes examined, and methodological quality. Over the long term, providing more rigorous evidence for interventions for children with ASD will contribute to positive outcomes for this population, enabling these individuals to contribute more effectively to the social and economic life of their communities. The past 40 years have seen many gains in the quality and quantity of intervention research for individuals with ASD. Research in this area has provided hope for many families and provided evidence that many individuals can learn and develop beyond earlier expectations. This systematic review summarizes this research and elucidates the many areas in which we have much to learn.

## Supporting Information

Supplement S1 Complete literature search strategy
(0.32 MB DOC)

Supplement S2 List of excluded studies
(0.39 MB DOC)
