# Supplementary material for: Behavioural and Developmental Interventions for Autism Spectrum Disorder: A Clinical Systematic Review
Source: PLoS One. 2008 Nov 18;3(11):e3755. doi: 10.1371/journal.pone.0003755 (PMC2582449; doi:10.1371/journal.pone.0003755)
Supplement: Supplement S1 — Complete literature search strategy (0.32 MB DOC) [file pone.0003755.s001.doc]

**Supplement A. Search Strategy**

Table A1. MEDLINE® – Ovid Version

Table A2. MEDLINE® In-Process and Other Non-Indexed Citations – Ovid Version

Table A3. PsycINFO® – Ovid Version

Table A4. ERIC – Ovid Version

Table A5. EMBASE – Ovid Version

Table A6. CINAHL® (Cumulative Index to Nursing & Allied Health Literature) – Ovid Version

Table A7. CINAHL® (Cumulative Index to Nursing & Allied Health Literature) – EBSCO Version

Table A8. AMED (Allied and Complementary Medicine) – Ovid Version

Table A9. Central (EBM Reviews - Cochrane Central Register of Controlled Trials) – Ovid Version

Table A10. PsychARTICLES (OVID Journals) – Ovid Version

Table A11. Web of Science® – Institute for Scientific Information – Thomson Corporation

Table A12. BIOSIS Previews® – Institute for Scientific Information – Thomson Corporation

Table A13. Social Sciences Abstracts

Table A14. Academic Search Premier – EBSCO Version

Table A15. Child Development and Adolescent Studies – EBSCO Version

Table A16. Linguistics and Language Behavior Abstracts

Table A17. Psychology and Behavioral Sciences Collection – EBSCO Version

Table A18. Cochrane Database of Systematic Reviews, Database of Abstracts of Reviews of Effects, Heath Technology Assessment Database, NHS Economic Evaluation Database – Wiley Version

Table A19. OCLC Papers First and OCLC Proceedings First – OCLC FirstSearch

Table A20. Dissertation Abstracts

Table A21. LILACS – OCLC FirstSearch

Table A22. NLM Gateway – National Library of Medicine

Table A23. DOAJ - Directory of Open Access Journals

**Databases searched for relevant studies**

| **Database** | **Years/issues** | **Date of search** |
| --- | --- | --- |
| Academic Search Premier | 1975 - 2007 | 08 May, 2007 |
| AMED | 1985 - 2007 | 03 May, 2007 |
| BIOSIS Previews | 1969 - 2007 | 09 May, 2007 |
| Child Development and Adolescent Studies | 1927 - 2007 | 08 May, 2007 |
| CINAHL® | 1982 - 2007 | 07 May, 2007 |
| Cochrane Central Register of Controlled Trials | 2nd Quarter 2007 | 03 May, 2007 |
| Cochrane Database of Systematic Reviews, Database of Abstracts of Reviews of Effects, HTA Database, NHS Economic Evaluation Database | 2nd Quarter 2007 | 10 May, 2007 |
| Dissertation Abstracts | 1931 - 2007 | 09 May, 2007 |
| DOAJ - Directory of Open Access Journals | 1977 - 2007 | 11 May, 2007 |
| EMBASE | 1988 - 2007 | 03 May, 2007 |
| ERIC | 1966 - 2007 | 03 May, 2007 |
| LILACS | 1982 - 2007 | 09 May, 2007 |
| Linguistics and Language Behavior Abstracts | 1973 - 2007 | 08 May, 2007 |
| MEDLINE® | 1966 - 2007 | 02 May, 2007 |
| MEDLINE® In-Process and Other Non-Indexed Citations | 1950 - 2007 | 15 May, 2007 |
| NLM Gateway | 1950 - 2007 | 10 May, 2007 |
| OCLC Papers First and OCLC Proceedings | 1993 - 2007 | 09 May, 2007 |
| PsycARTICLES (OVID Journals) | 1898 - 2007 | 03 May, 2007 |
| Psychology and Behavioral Sciences Collection | 1965 - 2007 | 08 May, 2007 |
| PsycINFO® | 1872 - 2007 | 02 May, 2007 |
| Social Sciences Abstracts | 1983 - 2007 | 04 May, 2007 |
| Web of Science® | 1900 - 2007 | 04 May, 2007 |

**Table A1. MEDLINE® - Ovid Version**

**Years/issue searched:** 1966 to 2007

**Search date:** 03 May, 2007

1. exp Child Development Disorders, Pervasive/

2. exp Autistic Disorder/

3. autis$.mp.

4. asd.ti,ab.

5. kanner$.ti,ab.

6. asperger$.ti,ab.

7. (pervasive and development and disorder).ti,ab.

8. PDD.ti,ab.

9. pdd-nos.ti,ab.

10. childhood disintegrative disorder.ti,ab.

11. ((speech or communicat$) adj3 disorder$).ti,ab.

12. (child$ adj3 schizophren$).ti,ab,sh.

13. (language adj3 delay$).ti,ab.

14. or/1-13

15. exp Behavior Therapy/

16. exp Imitative Behavior/

17. applied behavio?ral analy$.ti,ab,jn.

18. ABA.ti,ab.

19. intensive behavio?ral intervent$.ti,ab.

20. (IBI or IBT).ti,ab.

21. applied verbal behavio?r.ti,ab.

22. verbal behavio$.ti,ab.

23. (verbal adj5 (therap$ or communicat$)).ti,ab.

24. lovaas.ti,ab.

25. linwood.ti,ab.

26. Douglass.ti,ab.

27. CABAS.ti,ab.

28. DTT.ti,ab.

29. (Treatment adj2 Education adj2 Autistic adj communication adj Handicapped adj children).ti,ab.

30. teacch.ti,ab.

31. floor time.ti,ab.

32. "Social Communication Emotional Regulation Transactional Support".ti,ab.

33. scerts.ti,ab.

34. (pivotal adj 3 response).ti,ab.

35. discrete trial$.ti,ab.

36. (((sensory or auditory) adj integration) and (treat$ or therap$)).mp.

37. facilitated communication.ti,ab.

38. ((parent or parents or caregiver$ or care-giver$ or family or families or mother$ or father$ or maternal$ or paternal$) adj2 (treat$ or therap$ or interven$ or direct$ or program$ or train$ or mediat$ or rehabilit$)).mp.

39. Picture Exchange.ti,ab.

40. photic stimulation/ and (treat$ or therap$ or interven$ or direct$ or program$ or train$ or mediat$ or rehabilit$).mp.

41. exp Language Therapy/ or exp Speech Therapy/

42. occupational therapy/

43. exp Computer-Assisted Instruction/

44. (assist$ adj3 tech$).ti,ab.

45. exp Sensory Art Therapies/ or Play Therapy/

46. Early Intervention/

47. (computer adj3 (teach$ or instruct$)).ti,ab.

48. social stories.ti,ab.

49. prompt$.mp.

50. ((augment$ or social) adj3 communicat$).ti,ab.

51. (relationship adj develop$).ti,ab.

52. (cognitive and (treat$ or therap$ or psychotherap$)).mp.

53. cbt.ti,ab.

54. (sound adj3 (treat$ or therap$)).ti,ab.

55. (natural adj environment).ti,ab.

56. (activity adj schedule$).ti,ab.

57. (direct adj instruct$).ti,ab.

58. (giant adj step$).ti,ab.

59. developmental individual difference.ti,ab.

60. option.ti,ab.

61. (sonrise or kaufman).ti,ab.

62. precision.ti,ab.

63. (social adj skill$).ti,ab.

64. hanen.ti,ab.

65. miller.ti,ab.

66. patterning$.ti,ab.

67. philadelphia.ti,ab.

68. (dolman or delaccato).ti,ab.

69. (echange adj 3 developpement).ti,ab.

70. bartelemy.ti,ab.

71. (gentle adj teach$).ti,ab.

72. denver.ti,ab.

73. leap.ti,ab.

74. (learning experiences adj alternative program).ti,ab.

75. pcdi.ti,ab.

76. princeton child development institute.ti,ab,af.

77. rutgers.ti,ab.

78. (natural adj teach$).ti,ab.

79. milieu.ti,ab.

80. (neurodevelop$ adj treat$).ti,ab.

81. ndt.ti,ab.

82. walden.ti,ab.

83. adlerian.ti,ab.

84. theraplay.ti,ab.

85. Eden.ti,ab.

86. (social adj pragmatic).ti,ab.

87. "early bird".ti,ab.

88. (video adj3 model$).ti,ab.

89. (self adj3 (manage$ or monitor$)).ti,ab.

90. yale.ti,ab.

91. bancroft.ti,ab.

92. horizon.ti,ab.

93. (may adj institute).ti,ab.

94. or/15-93

95. 14 and 94

96. (200605$ or 200606$ or 200607$ or 200608$ or 200609$ or 20061$ or 2007$).ed.

97. clinical trial.pt.

98. randomi?ed.ti,ab.

99. placebo.ti,ab.

100. dt.fs.

101. randomly.ti,ab.

102. trial.ti,ab.

103. groups.ti,ab.

104. or/97-103

105. animals/

106. humans/

107. 105 not (105 and 106)

108. 104 not 107

109. RANDOMIZED CONTROLLED TRIAL.pt.

110. CONTROLLED CLINICAL TRIAL.pt.

111. RANDOMIZED CONTROLLED TRIALS/

112. RANDOM ALLOCATION/

113. DOUBLE BLIND METHOD/

114. SINGLE-BLIND METHOD/

115. or/109-114

116. ANIMAL/ not HUMAN/

117. 115 not 116

118. CLINICAL TRIAL.pt.

119. exp CLINICAL TRIALS/

120. (clin$ adj25 (trial$ or study or studies or design)).ti,ab.

121. ((singl$ or doubl$ or trebl$ or tripl$) adj25 (blind$ or mask$)).ti,ab.

122. PLACEBOS/

123. placebo$.ti,ab.

124. random$.ti,ab.

125. RESEARCH DESIGN/

126. or/118-125

127. 126 not 116

128. 127 not 117

129. COMPARATIVE STUDY/

130. exp EVALUATION STUDIES/

131. FOLLOW UP STUDIES/

132. (Follow up adj5 (study or studies or design)).ti,ab.

133. PROSPECTIVE STUDIES/

134. exp COHORT STUDIES/

135. CROSS-SECTIONAL STUDIES/

136. exp CASE-CONTROL STUDIES/

137. Epidemiologic studies/

138. Epidemiological factors/

139. exp Causality/

140. Age factors/

141. Comorbidity/

142. Odds ratio/

143. exp Risk/

144. Probability/

145. ((Allocat$ or control$ or assign$ or treatment or compar$ or interven$ or experiment$) and (group or groups)).mp.

146. (group or groups).ti,ab.

147. (control$ or prospectiv$ or retrospectiv$ or volunteer$ or participant$ or compar$).mp. and (trial$ or study or studies or design).ti,ab,sh. [mp=title, original title, abstract, name of substance word, subject heading word]

148. cohort$.ti,ab.

149. case-control$.ti,ab.

150. Cross sectional.ti,ab.

151. (observational adj5 (study or studies or design)).ti,ab.

152. Longitudinal.mp.

153. Retrospective.ti,ab.

154. Relative risk.ti,ab.

155. Odds ratio.ti,ab.

156. (case adj (comparison or referent)).ti,ab.

157. (Causation or causal$).ti,ab.

158. (Analytic adj (study or studies)).ti,ab.

159. single subject.mp. or SSRD.ti,ab.

160. "n-of-1".ti,ab.

161. or/129-160

162. 161 not 116

163. 162 not (117 or 128)

164. 117 or 128 or 162

165. 95 and 96 and (108 or 164)

**Table A2. MEDLINE® In-Process and Other Non-Indexed Citations – Ovid Version**

**Years/issue searched:** 1950 to 2007

**Search date:** 15 May, 2007

1. autis$.mp.

2. asd.ti,ab.

3. kanner$.ti,ab.

4. asperger$.ti,ab.

5. (pervasive and development and disorder).ti,ab.

6. PDD.ti,ab.

7. pdd-nos.ti,ab.

8. childhood disintegrative disorder.ti,ab.

9. ((speech or communicat$) adj3 disorder$).ti,ab.

10. (child$ adj3 schizophren$).ti,ab,sh.

11. (language adj3 delay$).ti,ab.

12. or/1-11

13. applied behavio?ral analy$.ti,ab,jn.

14. ABA.ti,ab.

15. intensive behavio?ral intervent$.ti,ab.

16. (IBI or IBT).ti,ab.

17. applied verbal behavio?r.ti,ab.

18. verbal behavio$.ti,ab.

19. (verbal adj5 (therap$ or communicat$)).ti,ab.

20. lovaas.ti,ab.

21. linwood.ti,ab.

22. Douglass.ti,ab.

23. CABAS.ti,ab.

24. DTT.ti,ab.

25. (Treatment adj2 Education adj2 Autistic adj communication adj Handicapped adj children).ti,ab.

26. teacch.ti,ab.

27. floor time.ti,ab.

28. "Social Communication Emotional Regulation Transactional Support".ti,ab.

29. scerts.ti,ab.

30. (pivotal adj 3 response).ti,ab.

31. discrete trial$.ti,ab.

32. (((sensory or auditory) adj integration) and (treat$ or therap$)).mp.

33. facilitated communication.ti,ab.

34. ((parent or parents or caregiver$ or care-giver$ or family or families or mother$ or father$ or maternal$ or paternal$) adj2 (treat$ or therap$ or interven$ or direct$ or program$ or train$ or mediat$ or rehabilit$)).mp.

35. Picture Exchange.ti,ab.

36. (assist$ adj3 tech$).ti,ab.

37. (direct adj instruct$).ti,ab.

38. (giant adj step$).ti,ab.

39. developmental individual difference.ti,ab.

40. option.ti,ab.

41. (sonrise or kaufman).ti,ab.

42. precision.ti,ab.

43. (social adj skill$).ti,ab.

44. hanen.ti,ab.

45. miller.ti,ab.

46. patterning$.ti,ab.

47. philadelphia.ti,ab.

48. (dolman or delaccato).ti,ab.

49. (echange adj 3 developpement).ti,ab.

50. bartelemy.ti,ab.

51. (gentle adj teach$).ti,ab.

52. denver.ti,ab.

53. leap.ti,ab.

54. (learning experiences adj alternative program).ti,ab.

55. pcdi.ti,ab.

56. princeton child development institute.ti,ab,af.

57. rutgers.ti,ab.

58. (natural adj teach$).ti,ab.

59. milieu.ti,ab.

60. (neurodevelop$ adj treat$).ti,ab.

61. ndt.ti,ab.

62. walden.ti,ab.

63. adlerian.ti,ab.

64. theraplay.ti,ab.

65. Eden.ti,ab.

66. (social adj pragmatic).ti,ab.

67. "early bird".ti,ab.

68. (video adj3 model$).ti,ab.

69. (self adj3 (manage$ or monitor$)).ti,ab.

70. yale.ti,ab.

71. bancroft.ti,ab.

72. horizon.ti,ab.

73. (may adj institute).ti,ab.

74. or/13-73

75. 12 and 74

**Table A3. PsycINFO® –Ovid Version**

**Years/issue searched:** 1872 to 2007

**Search date:** 02 May, 2007

1. exp Pervasive Developmental Disorders/

2. exp Autism/

3. exp Autistic Children/

4. autis$.mp.

5. kanner$.ti,ab.

6. asperger$.ti,ab.

7. (pervasive and development and disorder).ti,ab.

8. PDD.ti,ab.

9. pdd-nos.ti,ab.

10. childhood disintegrative disorder.ti,ab.

11. ((speech or communicat$) adj3 disorder$).ti,ab,sh.

12. (child$ adj3 schizophren$).ti,ab,sh.

13. (language adj3 delay$).ti,ab.

14. or/1-12

15. exp Behavior Therapy/

16. exp Cognitive Techniques/

17. exp Creative Arts Therapy/

18. exp "IMITATION (LEARNING)"/

19. exp Special Education/

20. Communication Skills Training/

21. Augmentative Communication/

22. applied behavio?ral analy$.ti,ab,jn.

23. ABA.ti,ab.

24. intensive behavio?ral intervent$.ti,ab.

25. (IBI or IBT).ti,ab.

26. applied verbal behavio?r.ti,ab.

27. verbal behavio$.ti,ab.

28. (verbal adj5 (therap$ or communicat$)).ti,ab.

29. exp Speech Therapy/

30. lovaas.ti,ab.

31. linwood.ti,ab.

32. Douglass.ti,ab.

33. CABAS.ti,ab.

34. DTT.ti,ab.

35. "Treatment and Education of Autistic and communication Handicapped children".ti,ab.

36. teacch.ti,ab.

37. floor time.ti,ab.

38. "Social Communication Emotional Regulation Transactional Support".ti,ab.

39. scerts.ti,ab.

40. (pivotal adj 3 response).ti,ab.

41. discrete trial$.ti,ab.

42. exp Sensory Integration/

43. (((sensory or auditory) adj integration) and (treat$ or therap$)).mp.

44. facilitated communication.ti,ab.

45. exp Parent Training/

46. ((parent or parents or caregiver$ or care-giver$ or family or families or mother$ or father$ or maternal$ or paternal$) adj2 (treat$ or therap$ or interven$ or direct$ or program$ or train$ or mediat$ or rehabilit$)).mp.

47. Picture Exchange.ti,ab.

48. (assist$ adj3 tech$).ti,ab.

49. exp Computer-Assisted Instruction/

50. Early Intervention/

51. (computer adj3 (teach$ or instruct$)).ti,ab.

52. social stories.ti,ab.

53. exp Prompting/

54. prompt$.mp.

55. ((augment$ or social) adj3 communicat$).ti,ab.

56. (relationship adj develop$).ti,ab.

57. (cognitive and (treat$ or therap$ or psychotherap$)).mp.

58. cbt.ti,ab.

59. (sound adj3 (treat$ or therap$)).ti,ab.

60. (natural adj environment).ti,ab.

61. (activity adj schedule$).ti,ab.

62. (direct adj instruct$).ti,ab.

63. (giant adj step$).ti,ab.

64. developmental individual difference.ti,ab.

65. option.ti,ab.

66. (sonrise or kaufman).ti,ab.

67. precision.ti,ab.

68. (social adj skill$).ti,ab.

69. hanen.ti,ab.

70. miller.ti,ab.

71. patterning$.ti,ab.

72. philadelphia.ti,ab.

73. (dolman or delaccato).ti,ab.

74. (echange adj 3 developpement).ti,ab.

75. bartelemy.ti,ab.

76. (gentle adj teach$).ti,ab.

77. denver.ti,ab.

78. leap.ti,ab.

79. (learning experiences adj alternative program).ti,ab.

80. pcdi.ti,ab.

81. princeton child development institute.ti,ab,af.

82. rutgers.ti,ab.

83. (natural adj teach$).ti,ab.

84. milieu.ti,ab.

85. (neurodevelop$ adj treat$).ti,ab.

86. ndt.ti,ab.

87. walden.ti,ab.

88. adlerian.ti,ab.

89. theraplay.ti,ab.

90. Eden.ti,ab.

91. (social adj pragmatic).ti,ab.

92. "early bird".ti,ab.

93. (video adj3 model$).ti,ab.

94. (self adj3 (manage$ or monitor$)).ti,ab.

95. (yale or bancroft or horizon).ti,ab.

96. "may institute".ti,ab.

97. or/15-96

98. 14 and 97

99. exp CLINICAL TRIALS/

100. control group/

101. random$.mp.

102. "sampling (experimental)"/ or Biased Sampling/ or Random Sampling/

103. ((singl$ or doubl$ or tripl$ or trebl$) adj10 (blind$ or mask$)).mp.

104. (cross?over or placebo$ or control$ or factorial or sham$).mp.

105. double dummy.mp.

106. ((clin$ or intervention$ or compar$ or experiment$ or preventive or therap$) adj10 (trial$ or study or studies)).mp.

107. Experimental Subjects/ or Experiment volunteers/ or Experiment controls/ or Experimental Replication/

108. clinical research.mp. or exp Treatment Effectiveness Evaluation/

109. Treatment Outcomes/ or Psychotherapeutic outcomes/

110. (outcome$ adj assessment).mp.

111. (longitudinal study or meta analysis or program evaluation or prospective study or retrospective study or treatment outcome study or empirical study or experimental replication or followup study).fc.

112. clinical case report.fc.

113. (clin$ adj25 (trial$ or study or studies or design)).ti,ab.

114. ((singl$ or doubl$ or trebl$ or tripl$) adj25 (blind$ or mask$)).ti,ab.

115. (efficacy or effective$ or findings or results).mp.

116. RESEARCH DESIGN/

117. FOLLOW-UP STUDIES/

118. (Follow up adj5 (study or studies or design)).ti,ab.

119. PROSPECTIVE STUDIES/

120. LONGITUDINAL STUDIES/

121. Comorbidity/

122. exp Probability/

123. ((Allocat$ or control$ or assign$ or treatment or compar$ or interven$ or experiment$) and (group or groups)).mp.

124. (group or groups).ti,ab.

125. ((control$ or multicenter or prospectiv$ or retrospectiv$ or evaluation or outcome$ or volunteer$ or subjects or participant$ or compar$) and (trial$ or study or studies or design)).mp.

126. Ss.ab.

127. cohort$.ti,ab.

128. case-control$.ti,ab.

129. Cross sectional.ti,ab.

130. (observational adj5 (study or studies or design)).ti,ab.

131. Longitudinal.mp.

132. Retrospective.ti,ab.

133. risk.ti,ab.

134. Odds ratio.ti,ab.

135. (case adj (comparison or referent)).ti,ab.

136. (Causation or causal$).ti,ab.

137. (Analytic adj (study or studies)).ti,ab.

138. exp Placebo/

139. exp Empirical Methods/

140. Repeated Measures/

141. Between Groups Design/

142. exp Evaluation/

143. cohort analysis/

144. single subject$.mp. or SSRD.ti,ab. or "n-of-1".ti,ab.

145. or/99-143

146. 98 and 145

147. limit 146 to human

148. limit 98 to ("0200 clinical case study" or "0400 empirical study" or "0410 experimental replication" or "0430 followup study" or "0450 longitudinal study" or "0451 prospective study" or "0452 retrospective study" or "0600 field study" or 1800 quantitative study)

149. limit 148 to human

150. 147 or 149

151. limit 150 to (classic book or handbook manual or reference book or "textbook/study guide")

152. 150 not 151

**Table A4. ERIC (Education Resources Information Center) – Ovid Version**

**Years/issue searched:** 1966 to 2007

**Search date:** 03 May, 2007

1. exp Pervasive Developmental Disorders/

2. exp Autism/

3. exp Asperger Syndrome/

4. autis$.mp.

5. kanner$.ti,ab.

6. asperger$.ti,ab.

7. (pervasive and development and disorder).ti,ab.

8. PDD.ti,ab.

9. pdd-nos.ti,ab.

10. childhood disintegrative disorder.ti,ab.

11. ((speech or communicat$) adj3 disorder$).ti,ab,sh.

12. (child$ adj3 schizophren$).ti,ab,sh.

13. (language adj3 delay$).ti,ab.

14. Delayed Speech/

15. or/1-13

16. exp Behavior Modification/

17. exp Behavior Change/

18. exp IMITATION/

19. exp Special Education/

20. exp Classroom Techniques/

21. Cognitive Restructuring/

22. (behavio$ adj5 (therap$ or interven$)).ti,ab.

23. applied behavio?ral analy$.ti,ab,jn.

24. ABA.ti,ab.

25. intensive behavio?ral intervent$.ti,ab.

26. (IBI or IBT).ti,ab.

27. exp Verbal Communication/

28. applied verbal behavio?r.ti,ab.

29. verbal behavio$.ti,ab.

30. (verbal adj5 (therap$ or communicat$)).ti,ab.

31. exp Speech Therapy/

32. occupational therapy/

33. Music Therapy/ or Art Therapy/ or Dance Therapy/ or Play Therapy/

34. lovaas.ti,ab.

35. linwood.ti,ab.

36. Douglass.ti,ab.

37. CABAS.ti,ab.

38. DTT.ti,ab.

39. (Treatment adj2 Education adj2 Autistic adj communication adj Handicapped adj children).ti,ab.

40. teacch.ti,ab.

41. floor time.ti,ab.

42. (Social adj Communication adj Emotional adj Regulation adj Transactional adj Support).ti,ab.

43. scerts.ti,ab.

44. (pivotal adj 3 response).ti,ab.

45. discrete trial$.ti,ab.

46. exp Sensory Integration/

47. (((sensory or auditory) adj integration) and (treat$ or therap$)).mp.

48. facilitated communication.ti,ab.

49. ((parent or parents or caregiver$ or care-giver$ or family or families or mother$ or father$ or maternal$ or paternal$) adj2 (treat$ or therap$ or interven$ or direct$ or program$ or train$ or mediat$ or rehabilit$)).mp.

50. Picture Exchange.ti,ab.

51. exp Computer-Assisted Instruction/

52. (assist$ adj3 tech$).ti,ab.

53. (computer adj3 (teach$ or instruct$)).ti,ab.

54. exp Early Intervention/

55. social stories.ti,ab.

56. exp Prompting/

57. prompt$.mp.

58. ((augment$ or social) adj3 communicat$).ti,ab.

59. (relationship adj develop$).ti,ab.

60. (cognitive and (treat$ or therap$ or psychotherap$)).mp.

61. cbt.ti,ab.

62. (natural adj environment).ti,ab.

63. (activity adj schedule$).ti,ab.

64. (direct adj instruct$).ti,ab.

65. (giant adj step$).ti,ab.

66. developmental individual difference.ti,ab.

67. option.ti,ab.

68. (sonrise or kaufman).ti,ab.

69. precision.ti,ab.

70. (social adj skill$).ti,ab.

71. hanen.ti,ab.

72. miller.ti,ab.

73. patterning$.ti,ab.

74. philadelphia.ti,ab.

75. (dolman or delaccato).ti,ab.

76. (echange adj 3 developpement).ti,ab.

77. bartelemy.ti,ab.

78. (gentle adj teach$).ti,ab.

79. denver.ti,ab.

80. leap.ti,ab.

81. (learning experiences adj alternative program).ti,ab.

82. pcdi.ti,ab.

83. princeton child development institute.ti,ab,af.

84. rutgers.ti,ab.

85. (natural adj teach$).ti,ab.

86. milieu.ti,ab.

87. (neurodevelop$ adj treat$).ti,ab.

88. ndt.ti,ab.

89. walden.ti,ab.

90. adlerian.ti,ab.

91. theraplay.ti,ab.

92. Eden.ti,ab.

93. (social adj pragmatic).ti,ab.

94. (early adj bird).ti,ab.

95. (video adj3 model$).ti,ab.

96. (self adj3 (manage$ or monitor$)).ti,ab.

97. (yale or bancroft or horizon).ti,ab.

98. (may adj institute).ti,ab.

99. or/16-98

100. 15 and 99

101. limit 100 to (books or "collected works (general and serials)" or conference proceedings or dissertations or "evaluative or feasibility reports" or general reports or information analyses or journal articles or project descriptions or "research or technical reports" or "speeches or conference papers" or statistical data or "tests or evaluation instruments")

**Table A5. EMBASE – Ovid Version**

**Years/issue searched:** 1988 to 2007

**Search date:** 03 May, 2007

1. autism/

2. asperger syndrome/

3. childhood disintegrative disorder/

4. infantile autism/

5. "pervasive developmental disorder not otherwise specified"/

6. autis$.mp.

7. asd.ti,ab.

8. kanner$.ti,ab.

9. asperger$.ti,ab.

10. (pervasive and development and disorder).ti,ab.

11. PDD.ti,ab.

12. pdd-nos.ti,ab.

13. childhood disintegrative disorder.ti,ab.

14. ((speech or communicat$) adj3 disorder$).ti,ab.

15. (child$ adj3 schizophren$).ti,ab,sh.

16. (language adj3 delay$).ti,ab.

17. or/1-16

18. exp Behavior Therapy/

19. cognitive therapy/

20. family therapy/

21. milieu therapy/

22. music therapy/

23. applied behavio?ral analy$.ti,ab,jn.

24. ABA.ti,ab.

25. intensive behavio?ral intervent$.ti,ab.

26. (IBI or IBT).ti,ab.

27. applied verbal behavio?r.ti,ab.

28. verbal behavio$.ti,ab.

29. (verbal adj5 (therap$ or communicat$)).ti,ab.

30. lovaas.ti,ab.

31. linwood.ti,ab.

32. Douglass.ti,ab.

33. CABAS.ti,ab.

34. DTT.ti,ab.

35. "Treatment and Education of Autistic and communication Handicapped children".ti,ab.

36. teacch.ti,ab.

37. floor time.ti,ab.

38. "Social Communication Emotional Regulation Transactional Support".ti,ab.

39. scerts.ti,ab.

40. (pivotal adj 3 response).ti,ab.

41. discrete trial$.ti,ab.

42. (((sensory or auditory) adj integration) and (treat$ or therap$)).mp.

43. facilitated communication.ti,ab,sh.

44. ((parent or parents or caregiver$ or care-giver$ or family or families or mother$ or father$ or maternal$ or paternal$) adj2 (treat$ or therap$ or interven$ or direct$ or program$ or train$ or mediat$ or rehabilit$)).mp.

45. Picture Exchange.ti,ab.

46. photostimulation/

47. exp Speech Therapy/

48. occupational therapy/

49. Play Therapy/

50. (computer adj3 (teach$ or instruct$)).ti,ab.

51. social stories.ti,ab.

52. prompt$.mp.

53. (augment$ adj communicat$).ti,ab.

54. (relationship adj develop$).ti,ab.

55. (cognitive and (treat$ or therap$ or psychotherap$)).mp.

56. cbt.ti,ab.

57. (sound adj3 (treat$ or therap$)).ti,ab.

58. (natural adj environment).ti,ab.

59. (activity adj schedule$).ti,ab.

60. (direct adj instruct$).ti,ab.

61. (giant adj step$).ti,ab.

62. developmental individual difference$.ti,ab.

63. option.ti,ab.

64. (sonrise or kaufman).ti,ab.

65. precision.ti,ab.

66. (social adj skill$).ti,ab.

67. hanen.ti,ab.

68. miller.ti,ab.

69. patterning$.ti,ab.

70. philadelphia.ti,ab.

71. (dolman or delaccato).ti,ab.

72. (echange adj 3 developpement).ti,ab.

73. bartelemy.ti,ab.

74. (gentle adj teach$).ti,ab.

75. denver.ti,ab.

76. leap.ti,ab.

77. (learning experiences adj alternative program).ti,ab.

78. pcdi.ti,ab.

79. princeton child development institute.ti,ab,af.

80. rutgers.ti,ab.

81. (natural adj teach$).ti,ab.

82. milieu.ti,ab.

83. (neurodevelop$ adj treat$).ti,ab.

84. ndt.ti,ab.

85. walden.ti,ab.

86. adlerian.ti,ab.

87. theraplay.ti,ab.

88. Eden.ti,ab.

89. (social adj pragmatic).ti,ab.

90. "early bird".ti,ab.

91. (video adj3 model$).ti,ab.

92. (self adj3 (manage$ or monitor$)).ti,ab.

93. yale.ti,ab.

94. bancroft.ti,ab.

95. horizon.ti,ab.

96. "may institute".ti,ab.

97. or/18-96

98. 17 and 97

99. Randomized Controlled Trial/

100. exp Randomization/

101. Double Blind Procedure/

102. Single Blind Procedure/

103. or/99-102

104. Clinical Trial/

105. (clin$ adj25 (trial$ or study or studies or design)).mp.

106. ((singl$ or doubl$ or trebl$ or tripl$) adj25 (blind$ or mask$)).mp.

107. exp Placebo/

108. (placebo$ or random$).mp.

109. exp Methodology/

110. exp Comparative Study/

111. exp Evaluation/

112. exp Follow Up/

113. exp Prospective Study/

114. clinical study/

115. case control study/

116. family study/

117. longitudinal study/

118. retrospective study/

119. cohort analysis/

120. exp Risk/

121. ((allocat$ or compar$ or assign$ or treatment or control$ or interven$ or experiment$) and (group or groups)).mp.

122. (group or groups).ti,ab.

123. ((control$ or prospectiv$ or retrospectiv$ or volunteer$ or participant$ or compar$) and (trial$ or study or studies or design)).ti,ab,sh.

124. cohort$.ti,ab.

125. "case-control".ti,ab. or "case report".mp.

126. "Cross sectional".ti,ab.

127. (observational adj5 (study or studies or design)).ti,ab.

128. Longitudinal.mp.

129. Retrospective.ti,ab.

130. "Relative risk".ti,ab.

131. "Odds ratio".ti,ab.

132. (Follow up adj5 (study or studies or design)).ti,ab.

133. (case adj (comparison or referent)).ti,ab.

134. (Causation or causal$).ti,ab.

135. (Analytic adj (study or studies)).ti,ab.

136. (epidemiologic$ adj (study or studies)).ti,ab.

137. single subject$.mp. or SSRD.ti,ab.

138. "n-of-1".ti,ab.

139. or/104-138

140. 103 or 139

141. limit 140 to human

142. Nonhuman/

143. 141 not 142

144. Randomized Controlled Trial/

145. exp Randomization/

146. Double Blind Procedure/

147. Single Blind Procedure/

148. or/14-147

149. 98 and 148

**Table A6. CINAHL® (Cumulative Index to Nursing & Allied Health Literature) – Ovid Version**

**Years/issue searched:** 1902 to June 2006

**Search date:** 05 June, 2006

1. exp Child Development Disorders, Pervasive/

2. exp Autistic Disorder/

3. autis$.mp.

4. asd.ti,ab.

5. kanner$.ti,ab.

6. asperger$.ti,ab.

7. (pervasive and development and disorder).ti,ab.

8. PDD.ti,ab.

9. pdd-nos.ti,ab.

10. childhood disintegrative disorder.ti,ab.

11. ((speech or communicat$) adj3 disorder$).mp.

12. (child$ adj3 schizophren$).ti,ab,sh.

13. (language adj3 delay$).ti,ab.

14. or/1-13

15. exp Behavior Therapy/

16. exp Social Skills Training/

17. applied behavio?ral analy$.ti,ab,jn.

18. ABA.ti,ab.

19. intensive behavio?ral intervent$.ti,ab.

20. (IBI or IBT).ti,ab.

21. applied verbal behavio?r.ti,ab.

22. verbal behavio$.ti,ab.

23. (verbal adj5 (therap$ or communicat$)).ti,ab.

24. lovaas.ti,ab.

25. linwood.ti,ab.

26. Douglass.ti,ab.

27. CABAS.ti,ab.

28. DTT.ti,ab.

29. (Treatment adj2 Education adj2 Autistic adj communication adj Handicapped adj children).ti,ab.

30. teacch.ti,ab.

31. floor time.ti,ab.

32. "Social Communication Emotional Regulation Transactional Support".ti,ab.

33. scerts.ti,ab.

34. (pivotal adj 3 response).ti,ab.

35. discrete trial$.ti,ab.

36. (((sensory or auditory) adj integration) and (treat$ or therap$)).mp.

37. Sensory Motor Integration/

38. facilitated communication.ti,ab.

39. Family Therapy/

40. ((parent or parents or caregiver$ or care-giver$ or family or families or mother$ or father$ or maternal$ or paternal$) adj2 (treat$ or therap$ or interven$ or direct$ or program$ or train$ or mediat$ or rehabilit$)).mp.

41. Picture Exchange.ti,ab.

42. Sensory stimulation/

43. exp Language Therapy/ or exp Speech Therapy/ or (Alternative adj Augmentative adj Communication).sh.

44. exp occupational therapy/

45. exp Computer-Assisted Instruction/

46. (assist$ adj3 tech$).ti,ab.

47. Dance Therapy/ or Music Therapy/ or Play Therapy/ or Socioenvironmental therapy/

48. Early Intervention/

49. (computer adj3 (teach$ or instruct$)).ti,ab.

50. social stories.ti,ab.

51. prompt$.mp.

52. ((augment$ or social) adj3 communicat$).ti,ab.

53. (relationship adj develop$).ti,ab.

54. (cognitive and (treat$ or therap$ or psychotherap$)).mp.

55. cbt.ti,ab.

56. (sound adj3 (treat$ or therap$)).ti,ab.

57. (natural adj environment).ti,ab.

58. (activity adj schedule$).ti,ab.

59. (direct adj instruct$).ti,ab.

60. (giant adj step$).ti,ab.

61. developmental individual difference.ti,ab.

62. option.ti,ab.

63. (sonrise or kaufman).ti,ab.

64. precision.ti,ab.

65. (social adj skill$).ti,ab.

66. hanen.ti,ab.

67. miller.ti,ab.

68. patterning$.ti,ab.

69. philadelphia.ti,ab.

70. (dolman or delaccato).ti,ab.

71. (echange adj 3 developpement).ti,ab.

72. bartelemy.ti,ab.

73. (gentle adj teach$).ti,ab.

74. denver.ti,ab.

75. leap.ti,ab.

76. (learning experiences adj alternative program).ti,ab.

77. pcdi.ti,ab.

78. princeton child development institute.ti,ab,af.

79. rutgers.ti,ab.

80. (natural adj teach$).ti,ab.

81. milieu.ti,ab.

82. (neurodevelop$ adj treat$).ti,ab.

83. ndt.ti,ab.

84. walden.ti,ab.

85. adlerian.ti,ab.

86. theraplay.ti,ab.

87. Eden.ti,ab.

88. (social adj pragmatic).ti,ab.

89. "early bird".ti,ab.

90. (video adj3 model$).ti,ab.

91. (self adj3 (manage$ or monitor$)).ti,ab.

92. yale.ti,ab.

93. bancroft.ti,ab.

94. horizon.ti,ab.

95. (may adj institute).ti,ab.

96. or/15-95

97. 14 and 96

**Table A7. CINAHL® (Cumulative Index to Nursing & Allied Health Literature) – EBSCO Version**

**Years/issue searched:** 2006 to 2007

**Search date:** 07 May, 2007

S47 ( S10 and S45 ) Limiters - Published Date from: 200601-200712; Publication Type: Abstract, Book, Book Chapter, Clinical Trial, Conference, Corrected Article, Doctoral Dissertation, Journal Article, Letter, Masters Thesis, Nursing Interventions, Practice Guidelines, Proceedings, Protocol, Research, Research Instrument, Research Instrument Utilization, Research Instrument Validation, Search Strategy, Systematic Review

S46 ( S10 and S45 )

S45 ( S44 or S43 or S42 or S41 or S40 or S39 or S38 or S37 or S36 or S35 or S34 or S33 or S32 or S31 or S30 or S29 or S28 or S27 or S26 or S25 or S24 or S23 or S22 or S44 or S43 or S42 or S41 or S40 or S39 or S38 or S37 or S36 or S35 or S34 )

S44 ( yale or bancroft or horizon ) or may N1 institute

S43 video N3 model* or self N3 manage* or self N3 monitor*

S42 ( ndt or walden or adlerian or theraplay or Eden ) or social N1 pragmatic or "early bird"

S41 natural N1 teach* or milieu or neurodevelop* N1 treat*

S40 pcdi or "princeton child development institute" or rutgers

S39 ( denver or leap ) or experiences N1 "alternative program"

S38 echange N3 developpement or bartelemy or gentle N1 teach*

S37 giant N1 step* or developmental W1 individual W1 difference or option

S36 ( sonrise or kaufman or precision ) or social N1 skill* or ( hanen or miller or patterning* or philadelphia or dolman or delaccato )

S35 natural N1 environment or activity N1 schedule* or direct N1 instruct*

S34 sound N3 treat* or sound N3 therap*

S33 cbt

S32 cognitive and ( treat* or therap* or psychotherap* )

S31 relationship N1 develop

S30 augment* N3 communicat* or social N3 communicat*

S29 social W1 stories or prompt*

S28 computer N3 teach* or computer N3 instruct*

S27 ( MH “Dance Therapy” or MH “Music Therapy” or MH “Play Therapy” or MH “Socioenvironmental therapy” ) or MH "Early Intervention"

S26 MH "occupational therapy+" or MH "Computer-Assisted Instruction+" or assist* N3 tech3

S25 MH "Language Therapy+" or MH "Speech Therapy+" or Alternative N1 Augmentative N1 Communication

S24 Picture Exchange or MH "Sensory stimulation"

S23 ( parent or parents or caregiver* or care-giver* or family or families or mother* or father* or maternal* or paternal* ) and ( treat* or therap* or interven* or direct* or program* or train* or mediat* or rehabilit* )

S22 pivotal N3 response* or discrete N1 trial*

S21 floor w1 time or "Social Communication Emotional Regulation Transactional Support" or scerts

S20 MH "Sensory Motor Integration" or facilitated N1 communicat* or MH "Family Therapy"

S19 ( sensory or auditory ) and integration

S18 Treatment N1 Education N1 Autistic N1 Communication N1 Handicapped N1 children

S17 ( lovaas or linwood or Douglass or CABAS or DTT or teacch )

S16 verbal N5 therap* or verbal N5 communicat*

S15 verbal N1 behavio*

S14 ( ABA or IBI or IBT ) and intensive W1 behavio* W1 intervent*

S13 applied W1 behavio* W1 analy*

S12 (MH Social Skills Training+)

S11 (MH Behavior Therapy+)

S10 ( (S9 or S8 or S7 or S6 or S5 or S4 or S3 or S2 or S1) )

S9 language N3 delay*

S8 child* N3 schizophren*

S7 communicat* N3 disorder*

S6 speech N3 disorder*

S5 ( pervasive and develop* and disorder* )

S4 “childhood disintegrative disorder”

S3 ( autis* or ASD or kanner* or asperger* or PDD or PDD-NOS )

S2 (MH "Autistic Disorder")

S1 (MH "Child Development Disorders, Pervasive+")

**Table A8. AMED (Allied and Complementary Medicine) Ovid Version**

**Years/issue searched:** 1985 to 2007

**Search date:** 03 May, 2007

1. exp Autistic Disorder/

2. autis$.mp.

3. asd.ti,ab.

4. kanner$.ti,ab.

5. asperger$.ti,ab.

6. (pervasive and development and disorder).ti,ab.

7. PDD.ti,ab.

8. pdd-nos.ti,ab.

9. childhood disintegrative disorder.ti,ab.

10. ((speech or communicat$) adj3 disorder$).mp.

11. Schizophrenia/ and (child$ or adolesc$ or teen or teenage$).mp.

12. (child$ adj3 schizophren$).ti,ab,sh.

13. (language adj3 delay$).ti,ab.

14. or/1-13

15. exp Behavior Therapy/

16. Milieu Therapy/

17. applied behavio?ral analy$.ti,ab,jn.

18. ABA.ti,ab.

19. intensive behavio?ral intervent$.ti,ab.

20. (IBI or IBT).ti,ab.

21. applied verbal behavio?r.ti,ab.

22. verbal behavio$.ti,ab.

23. (verbal adj5 (therap$ or communicat$)).ti,ab.

24. lovaas.ti,ab.

25. linwood.ti,ab.

26. Douglass.ti,ab.

27. CABAS.ti,ab.

28. DTT.ti,ab.

29. (Treatment adj2 Education adj2 Autistic adj communication adj Handicapped adj children).ti,ab.

30. teacch.ti,ab.

31. floor time.ti,ab.

32. "Social Communication Emotional Regulation Transactional Support".ti,ab.

33. scerts.ti,ab.

34. (pivotal adj 3 response).ti,ab.

35. discrete trial$.ti,ab.

36. (((sensory or auditory) adj integration) and (treat$ or therap$)).mp.

37. Sensory Integration/

38. facilitated communication.ti,ab.

39. Family Therapy/

40. ((parent or parents or caregiver$ or care-giver$ or family or families or mother$ or father$ or maternal$ or paternal$) adj2 (treat$ or therap$ or interven$ or direct$ or program$ or train$ or mediat$ or rehabilit$)).mp.

41. Picture Exchange.ti,ab.

42. exp Language Therapy/ or exp Speech Therapy/

43. exp Computer-Assisted Instruction/

44. (assist$ adj3 tech$).ti,ab.

45. Dance Therapy/ or Music Therapy/ or Play Therapy/

46. (computer adj3 (teach$ or instruct$)).ti,ab.

47. social stories.ti,ab.

48. prompt$.mp.

49. ((augment$ or social) adj3 communicat$).ti,ab.

50. (relationship adj develop$).ti,ab.

51. (cognitive and (treat$ or therap$ or psychotherap$)).mp.

52. cbt.ti,ab.

53. (sound adj3 (treat$ or therap$)).ti,ab.

54. (natural adj environment).ti,ab.

55. (activity adj schedule$).ti,ab.

56. (direct adj instruct$).ti,ab.

57. (giant adj step$).ti,ab.

58. developmental individual difference.ti,ab.

59. option.ti,ab.

60. (sonrise or kaufman).ti,ab.

61. precision.ti,ab.

62. (social adj skill$).ti,ab.

63. hanen.ti,ab.

64. miller.ti,ab.

65. patterning$.ti,ab.

66. philadelphia.ti,ab.

67. (dolman or delaccato).ti,ab.

68. (echange adj 3 developpement).ti,ab.

69. bartelemy.ti,ab.

70. (gentle adj teach$).ti,ab.

71. denver.ti,ab.

72. leap.ti,ab.

73. (learning experiences adj alternative program).ti,ab.

74. pcdi.ti,ab.

75. princeton child development institute.ti,ab,af.

76. rutgers.ti,ab.

77. (natural adj teach$).ti,ab.

78. milieu.ti,ab.

79. (neurodevelop$ adj treat$).ti,ab.

80. ndt.ti,ab.

81. walden.ti,ab.

82. adlerian.ti,ab.

83. theraplay.ti,ab.

84. Eden.ti,ab.

85. (social adj pragmatic).ti,ab.

86. "early bird".ti,ab.

87. (video adj3 model$).ti,ab.

88. (self adj3 (manage$ or monitor$)).ti,ab.

89. yale.ti,ab.

90. bancroft.ti,ab.

91. horizon.ti,ab.

92. (may adj institute).ti,ab.

93. or/15-92

94. 14 and 93

95. limit 94 to (brief report or brief research report or congress proceedings or journal article or meeting paper or meeting report or monograph or proceedings or report or symposium or thesis)

**Table A9. Central (EBM Reviews - Cochrane Central Register of Controlled Trials) – Ovid Version**

**Years/issue searched:** 1898 to 2007

**Search date:** 03 May, 2007

1. exp Child Development Disorders, Pervasive/

2. exp Autistic Disorder/

3. autis$.mp.

4. asd.ti,ab.

5. kanner$.ti,ab.

6. asperger$.ti,ab.

7. (pervasive and development and disorder).ti,ab.

8. PDD.ti,ab.

9. pdd-nos.ti,ab.

10. childhood disintegrative disorder.ti,ab.

11. ((speech or communicat$) adj3 disorder$).ti,ab.

12. (child$ adj3 schizophren$).ti,ab,sh.

13. (language adj3 delay$).ti,ab.

14. or/1-13

15. exp Behavior Therapy/

16. exp Imitative Behavior/

17. applied behavio?ral analy$.ti,ab,jn.

18. ABA.ti,ab.

19. intensive behavio?ral intervent$.ti,ab.

20. (IBI or IBT).ti,ab.

21. applied verbal behavio?r.ti,ab.

22. verbal behavio$.ti,ab.

23. (verbal adj5 (therap$ or communicat$)).ti,ab.

24. lovaas.ti,ab.

25. linwood.ti,ab.

26. Douglass.ti,ab.

27. CABAS.ti,ab.

28. DTT.ti,ab.

29. (Treatment adj2 Education adj2 Autistic adj communication adj Handicapped adj children).ti,ab.

30. teacch.ti,ab.

31. floor time.ti,ab.

32. "Social Communication Emotional Regulation Transactional Support".ti,ab.

33. scerts.ti,ab.

34. (pivotal adj 3 response).ti,ab.

35. discrete trial$.ti,ab.

36. (((sensory or auditory) adj integration) and (treat$ or therap$)).mp.

37. facilitated communication.ti,ab.

38. ((parent or parents or caregiver$ or care-giver$ or family or families or mother$ or father$ or maternal$ or paternal$) adj2 (treat$ or therap$ or interven$ or direct$ or program$ or train$ or mediat$ or rehabilit$)).mp.

39. Picture Exchange.ti,ab.

40. photic stimulation/ and (treat$ or therap$ or interven$ or direct$ or program$ or train$ or mediat$ or rehabilit$).mp.

41. exp Language Therapy/ or exp Speech Therapy/

42. occupational therapy/

43. exp Computer-Assisted Instruction/

44. (assist$ adj3 tech$).ti,ab.

45. exp Sensory Art Therapies/ or Play Therapy/

46. Early Intervention/

47. (computer adj3 (teach$ or instruct$)).ti,ab.

48. social stories.ti,ab.

49. prompt$.mp.

50. ((augment$ or social) adj3 communicat$).ti,ab.

51. (relationship adj develop$).ti,ab.

52. (cognitive and (treat$ or therap$ or psychotherap$)).mp.

53. cbt.ti,ab.

54. (sound adj3 (treat$ or therap$)).ti,ab.

55. (natural adj environment).ti,ab.

56. (activity adj schedule$).ti,ab.

57. (direct adj instruct$).ti,ab.

58. (giant adj step$).ti,ab.

59. developmental individual difference.ti,ab.

60. option.ti,ab.

61. (sonrise or kaufman).ti,ab.

62. precision.ti,ab.

63. (social adj skill$).ti,ab.

64. hanen.ti,ab.

65. miller.ti,ab.

66. patterning$.ti,ab.

67. philadelphia.ti,ab.

68. (dolman or delaccato).ti,ab.

69. (echange adj 3 developpement).ti,ab.

70. bartelemy.ti,ab.

71. (gentle adj teach$).ti,ab.

72. denver.ti,ab.

73. leap.ti,ab.

74. (learning experiences adj alternative program).ti,ab.

75. pcdi.ti,ab.

76. princeton child development institute.ti,ab,af.

77. rutgers.ti,ab.

78. (natural adj teach$).ti,ab.

79. milieu.ti,ab.

80. (neurodevelop$ adj treat$).ti,ab.

81. ndt.ti,ab.

82. walden.ti,ab.

83. adlerian.ti,ab.

84. theraplay.ti,ab.

85. Eden.ti,ab.

86. (social adj pragmatic).ti,ab.

87. "early bird".ti,ab.

88. (video adj3 model$).ti,ab.

89. (self adj3 (manage$ or monitor$)).ti,ab.

90. yale.ti,ab.

91. bancroft.ti,ab.

92. horizon.ti,ab.

93. (may adj institute).ti,ab.

94. or/15-93

95. 14 and 94

**Table A10. PsychARTICLES (OVID Journals) – Ovid Version**

**Years/issue searched:** 1898 to 2007

**Search date:** 03 May, 2007

1. autis$.ti,ab,jn.

2. asd.ti,ab.

3. kanner$.ti,ab.

4. asperger$.ti,ab.

5. (pervasive and development and disorder).ti,ab.

6. PDD.ti,ab.

7. pdd-nos.ti,ab.

8. childhood disintegrative disorder.ti,ab.

9. ((speech or communicat$) adj3 disorder$).ti,ab.

10. (child$ adj3 schizophren$).ti,ab,sh.

11. (language adj3 delay$).ti,ab.

12. or/1-11

13. (behavio?r adj therap$).ti,ab.

14. (Imitat$ adj2 Behavio?r).ti,ab.

15. applied behavio?ral analy$.ti,ab,jn.

16. ABA.ti,ab.

17. intensive behavio?ral intervent$.ti,ab.

18. (IBI or IBT).ti,ab.

19. applied verbal behavio?r.ti,ab.

20. verbal behavio$.ti,ab.

21. (verbal adj5 (therap$ or communicat$)).ti,ab.

22. lovaas.ti,ab.

23. linwood.ti,ab.

24. Douglass.ti,ab.

25. CABAS.ti,ab.

26. DTT.ti,ab.

27. (Treatment adj2 Education adj2 Autistic adj communication adj Handicapped adj children).ti,ab.

28. teacch.ti,ab.

29. floor time.ti,ab.

30. "Social Communication Emotional Regulation Transactional Support".ti,ab.

31. scerts.ti,ab.

32. (pivotal adj 3 response).ti,ab.

33. discrete trial$.ti,ab.

34. (((sensory or auditory) adj integration) and (treat$ or therap$)).ti,ab.

35. facilitated communication.ti,ab.

36. ((parent or parents or caregiver$ or care-giver$ or family or families or mother$ or father$ or maternal$ or paternal$) adj2 (treat$ or therap$ or interven$ or direct$ or program$ or train$ or mediat$ or rehabilit$)).ti,ab.

37. Picture Exchange.ti,ab.

38. photic stimulation.ti,ab. and (treat$ or therap$ or interven$ or direct$ or program$ or train$ or mediat$ or rehabilit$).mp.

39. ((Language or speech or occupation$) adj therap$).ti,ab.

40. "Computer-Assisted Instruction".ti,ab.

41. (assist$ adj3 tech$).ti,ab.

42. ((Art or play) adj Therap$).ti,ab.

43. "Early Intervention".ti,ab.

44. (computer adj3 (teach$ or instruct$)).ti,ab.

45. social stories.ti,ab.

46. prompt$.mp.

47. ((augment$ or social) adj3 communicat$).ti,ab.

48. (relationship adj develop$).ti,ab.

49. (cognitive and (treat$ or therap$ or psychotherap$)).mp.

50. cbt.ti,ab.

51. (sound adj3 (treat$ or therap$)).ti,ab.

52. (natural adj environment).ti,ab.

53. (activity adj schedule$).ti,ab.

54. (direct adj instruct$).ti,ab.

55. (giant adj step$).ti,ab.

56. developmental individual difference.ti,ab.

57. option.ti,ab.

58. (sonrise or kaufman).ti,ab.

59. precision.ti,ab.

60. (social adj skill$).ti,ab.

61. hanen.ti,ab.

62. miller.ti,ab.

63. patterning$.ti,ab.

64. philadelphia.ti,ab.

65. (dolman or delaccato).ti,ab.

66. (echange adj 3 developpement).ti,ab.

67. bartelemy.ti,ab.

68. (gentle adj teach$).ti,ab.

69. denver.ti,ab.

70. leap.ti,ab.

71. (learning experiences adj alternative program).ti,ab.

72. pcdi.ti,ab.

73. princeton child development institute.ti,ab,af.

74. rutgers.ti,ab.

75. (natural adj teach$).ti,ab.

76. milieu.ti,ab.

77. (neurodevelop$ adj treat$).ti,ab.

78. ndt.ti,ab.

79. walden.ti,ab.

80. adlerian.ti,ab.

81. theraplay.ti,ab.

82. Eden.ti,ab.

83. (social adj pragmatic).ti,ab.

84. "early bird".ti,ab.

85. (video adj3 model$).ti,ab.

86. (self adj3 (manage$ or monitor$)).ti,ab.

87. yale.ti,ab.

88. bancroft.ti,ab.

89. horizon.ti,ab.

90. (may adj institute).ti,ab.

91. or/13-90

92. 12 and 91

93. limit 92 to (meeting abstracts or original articles or reports or "review articles")

94. limit 93 to psycarticles

**Table A11. Web of Science® – Institute for Scientific Information – Thomson Corporation**

**Years/issue searched:** 1900 to 2007

**Search date:** 04 May, 2007

#20 #19 AND #18 DocType=Article OR Bibliography OR Correction OR Meeting Abstract OR Meeting-Abstract OR Reprint OR Review; Language=All languages;

#19#17 OR #16 OR #15 OR #14 OR #13 OR #12 OR #11 OR #10 OR #9 OR #8 OR #7 OR #6 OR #5 OR #4 OR #3

#18 #2 OR #1*;*

#17 TS=((neurodevelop* SAME treat*) or ndt or walden or adlerian or theraplay or Eden or (social SAME pragmatic) or early bird or (video SAME model*) or (self SAME (manage* or monitor*)) or yale or bancroft or horizon or may institute)

#16 TS=(denver or leap or (learning experiences SAME alternative program) or pcdi or princeton child development institute or rutgers or (natural SAME teach*) or milieu)

#15 TS=(precision or (social SAME skill*) or hanen or miller or patterning* or philadelphia or (dolman or delaccato) or (echange SAME developpement) or bartelemy or (gentle SAME teach*))

#14 TS=(cbt or (sound SAME (treat* or therap*)) or (natural SAME environment) or (activity SAME schedule*) or (direct SAME instruct*) or (giant SAME step*) or developmental individual difference or option or (sonrise or kaufman))

#13 TS=(social stories or prompt* or ((augment* or social) SAME communicat*) or (relationship SAME develop*) or (cognitive and (treat* or therap* or psychotherap*)))

#12 TS=(Dance Therapy or Music Therapy or Play Therapy or Socioenvironmental therapy or Early Intervention or (computer SAME (teach* or instruct*)))

#11 TS=(Picture Exchange or Sensory stimulation or Language Therapy or Speech Therapy or (Alternative SAME Augmentative SAME Communication) or occupational therapy or Computer-Assisted Instruction or (assist* SAME tech*))

#10 TS=((parent or parents or caregiver* or care-giver* or family or families or mother* or father* or maternal* or paternal*) SAME (treat* or therap* or interven* or direct* or program* or train* or mediat* or rehabilit*))

#9 TS=(Sensory Motor Integration or facilitated communication or Family Therapy)

#8 TS=((pivotal SAME response) or discrete trial* or (((sensory or auditory) SAME integration) and (treat* or therap*)))

#7 TS=(teacch or floor time or Social Communication Emotional Regulation Transactional Support or scerts)

#6 TS=(Treatment SAME Education SAME Autistic SAME communication SAME Handicapped SAME children)

#5 TS=(lovaas or linwood or Douglass or CABAS or DTT)

#4 TS=(verbal behavio* or (verbal SAME (therap* or communicat*)))

#3 TS=(Behavior Therapy or Social Skills Training or (applied SAME behavio* SAME analy*) or ABA or (intensive behavio* SAME intervent*) or (IBI or IBT))

#2 TS=(Kanner* or Speech Disorder* or Communication disorder* or Autis* or Asperger* or PDD or PDD-NOS or Childhood Disintegrative Disorder* or Childhood Schizophrenia)

#1 TS=(Pervasive SAME Development Disorder*)

**Table A12. BIOSIS Previews® – Institute for Scientific Information – Thomson Corporation**

**Years/issue searched:** 1969 to 2007

**Search date:** 09 May, 2007

#20 #19 AND #18 DocType=Article OR Article Thesis Dissertation OR Book Chapter OR Meeting OR Meeting Paper OR Technical Report OR Thesis Dissertation; LitType=All literature types; Language=All languages; Taxa Notes=Humans;

#19 #17 OR #16 OR #15 OR #14 OR #13 OR #12 OR #11 OR #10 OR #9 OR #8 OR #7 OR #6 OR #5 OR #4 OR #3

#18 #2 OR #1

#17 TS=(Treatment SAME Education SAME Autistic SAME communication SAME Handicapped SAME children)

#16 TS=((neurodevelop* SAME treat*) or ndt or walden or adlerian or theraplay or Eden or (social SAME pragmatic) or early bird or (video SAME model*) or (self SAME (manage* or monitor*)) or yale or bancroft or horizon or may institute)

#15 TS=(denver or leap or (learning experiences SAME alternative program) or pcdi or princeton child development institute or rutgers or (natural SAME teach*) or milieu)

#14 TS=(precision or (social SAME skill*) or hanen or miller or patterning* or philadelphia or (dolman or delaccato) or (echange SAME developpement) or bartelemy or (gentle SAME teach*))

#13 TS=(cbt or (sound SAME (treat* or therap*)) or (natural SAME environment) or (activity SAME schedule*) or (direct SAME instruct*) or (giant SAME step*) or developmental individual difference or option or (sonrise or kaufman))

#12 TS=(social stories or prompt* or ((augment* or social) SAME communicat*) or (relationship SAME develop*) or (cognitive and (treat* or therap* or psychotherap*)))

#11 TS=(Dance Therapy or Music Therapy or Play Therapy or Socioenvironmental therapy or Early Intervention or (computer SAME (teach* or instruct*)))

#10 TS=(Picture Exchange or Sensory stimulation or Language Therapy or Speech Therapy or (Alternative SAME Augmentative SAME Communication) or occupational therapy or Computer-Assisted Instruction or (assist* SAME tech*))

#9 TS=((parent or parents or caregiver* or care-giver* or family or families or mother* or father* or maternal* or paternal*) SAME (treat* or therap* or interven* or direct* or program* or train* or mediat* or rehabilit*))

#8 TS=(Sensory Motor Integration or facilitated communication or Family Therapy)

#7 TS=((pivotal SAME response) or discrete trial* or (((sensory or auditory) SAME integration) and (treat* or therap*)))

#6 TS=(teacch or floor time or Social Communication Emotional Regulation Transactional Support or scerts)

#5 TS=(Treatment SAME Education SAME Autistic SAME communication SAME Handicapped SAME children)

#4 TS=(lovaas or linwood or Douglass or CABAS or DTT)

#3 TS=(Behavior Therapy or Social Skills Training or (applied SAME behavio* SAME analy*) or ABA or (intensive behavio* SAME intervent*) or (IBI or IBT))

#2 TS=(Kanner* or Speech Disorder* or Communication disorder* or Autis* or Asperger* or PDD or PDD-NOS or Childhood Disintegrative Disorder* or Childhood Schizophrenia)

#1 TS=(Pervasive SAME Development Disorder*)

**Table A13. Social Sciences Abstracts**

**Years/issue searched:** 1983 to 2007

**Search date:** 04 May, 2007

Pervasive Development Disorder* or Kanner* or Speech Disorder* or Communication Disorder* or AutiS* or Asperger* or PDD or PDD-NOS

AND

("Behavior Therapy" or IBI or IBT)

precision or (social W2 skill*) or hanen or miller or patterning* or philadelphia or (dolman W2 delaccato)

"social stories" or prompt* or (social W2 communicat*) or (relationship W2 develop*) or (cognitive and (treat* or therap* or psychother*))

"Picture Exchange" or "Sensory stimulation" or "Language Therapy" or "Speech Therapy"

(direct W2 instruct*) or (giant W2 step*) or developmental individual difference or option or (sonrise or kaufman)

CBT or (sound W2 treat*) or (sound W2 therap*) or (natural W2 environment) or (activity W2 schedule*) or (Augment* W3 Communicat*) OR "occupational therapy" or "Computer-Assisted Instruction" or (assist* W tech*)

pcdi or "princeton child development institute" or rutgers or (natural W2 teach*) or milieu or (echange W2 developpement) or bartelemy or (gentle NEAR teach*) or denver or leap or (learning experiences W2 alternative program)

"Early Intervention" or (computer W2 teach*) or (computer w2 instruct*) or (neurodevelop* W2 treat*) or ndt or walden or adlerian or theraplay or Eden or (social W2 pragmatic)

("early bird" or (video W2 model*) or (self W2 manage*) or (self w2 monitor*)

Skills Training or applied behavio?ral analy* or ABA

applied verbal behavio?r or verbal behavio*

((Augment* W3 Communicat*) OR "occupational therapy" or "Computer-Assisted Instruction" or (assist* W1 tech*))

yale or bancroft or horizon or “may institute”

(Dance or Music or Play or Socioenvironmental) and Therap*

((parent* or caregive* or care-giver* or family or families or mother* or father*) and (treat* or thera* or interv* or direct* or program*))

verbal W2 (therap* or communicat*)) or lovaas or linwood or Douglass or CABAS or DTT

(Treatment W2 Education W2 Autistic W2 communication W2 Handicapped W2 children) or

teacch or “floor time” or "Social Communication Emotional Regulation Transactional Support"

scerts or (pivotal W2 response) or discrete trial* or (((sensory or auditory) W2 integration) and (treat* or therap*)) or

“Sensory Motor Integration” or “facilitated communication”

**Table A14. Academic Search Premier – EBSCO Version**

**Years/issue searched:** 1975 to 2007

**Search date:** 08 May, 2007

S24 ( S23 or S22 or S21 or S20 or S19 or S18 or S17 or S16 or S15 or S14 or S13 or S12 or S11 or S10 or S9 or S8 or S7 or S6 or S5 or S4 or S3 or S2 ) and S1

S23 ( "Behavior Therapy" or IBI or IBT )

S22 ( precision or (social W2 skill*) or hanen or miller or patterning* or philadelphia or (dolman W2 delaccato) )

S21 ( "social stories" or prompt* or (social W2 communicat*) or (relationship W2 develop*) or (cognitive and (treat* or therap* or psychother*)) )

S20 ( "Picture Exchange" or "Sensory stimulation" or "Language Therapy" or "Speech Therapy" )

S19 ( (direct W2 instruct*) or (giant W2 step*) or developmental individual difference or option or (sonrise or kaufman) )

S18 ( CBT or (sound W2 treat*) or (sound W2 therap*) or (natural W2 environment) or (activity W2 schedule*) or (Augment* W3 Communicat*) OR "occupational therapy" or "Computer-Assisted Instruction" or (assist* W tech*) )

S17 ( pcdi or "princeton child development institute" or rutgers or (natural W2 teach*) or milieu or (echange W2 developpement) or bartelemy or (gentle NEAR teach*) or denver or leap or (learning experiences W2 alternative program) or "Computer-Assisted Instruction" or (assist* W tech*) )

S16 ( "Early Intervention" or (computer W2 teach*) or (computer w2 instruct*) ) or ( (neurodevelop* W2 treat*) or ndt or walden or adlerian or theraplay or Eden or (social W2 pragmatic) )

S15 ( "early bird" or (video W2 model*) ) or self W2 manage* or self w2 monitor*

S14 ( "Skills Training" or ABA ) or applied W1 behavio?ral W1 analy*

S13 ( applied verbal behavio?r or verbal behavio* )

S12 Augment* W3 Communicat* and ( "occupational therapy" or "Computer-Assisted Instruction" ) and assist* W1 tech*

S11 ( yale or bancroft or horizon or “may institute” )

S10 ( Dance or Music or Play or Socioenvironmental ) and Therap*

S9 ( parent* or caregive* or care-giver* or family or families or mother* or father* ) and ( treat* or thera* or interv* or direct* or program* )

S8 verbal W2 communicat* or verbal W2 therap*

S7 ( lovaas or linwood or Douglass or CABAS or DTT )

S6 (Treatment W2 Education W2 Autistic W2 communication W2 Handicapped W2 children)

S5 ( teacch or “floor time” or "Social Communication Emotional Regulation Transactional Support" )

S4 scerts or pivotal W2 response or discrete trial*

S3 ( sensory or auditory ) and ( treat* or therap* ) and integration

S2 ( “Sensory Motor Integration” or “facilitated communication” )

S1 ( Pervasive Development Disorder* or Kanner* or Speech Disorder* or Communication Disorder* or AutiS* or Asperger* or PDD or PDD-NOS )

**Table A15. Child Development and Adolescent Studies – EBSCO Version**

**Years/issue searched:** 1927 to 2007

**Search date:** 08 May, 2007

S24 ( S23 or S22 or S21 or S20 or S19 or S18 or S17 or S16 or S15 or S14 or S13 or S12 or S11 or S10 or S9 or S8 or S7 or S6 or S5 or S4 or S3 or S2 ) and S1

S23 ( "Behavior Therapy" or IBI or IBT )

S22 ( precision or (social W2 skill*) or hanen or miller or patterning* or philadelphia or (dolman W2 delaccato) )

S21 ( "social stories" or prompt* or (social W2 communicat*) or (relationship W2 develop*) or (cognitive and (treat* or therap* or psychother*)) )

S20 ( "Picture Exchange" or "Sensory stimulation" or "Language Therapy" or "Speech Therapy" )

S19 ( (direct W2 instruct*) or (giant W2 step*) or developmental individual difference or option or (sonrise or kaufman) )

S18 ( CBT or (sound W2 treat*) or (sound W2 therap*) or (natural W2 environment) or (activity W2 schedule*) or (Augment* W3 Communicat*) OR "occupational therapy" or "Computer-Assisted Instruction" or (assist* W tech*) )

S17 ( pcdi or "princeton child development institute" or rutgers or (natural W2 teach*) or milieu or (echange W2 developpement) or bartelemy or (gentle NEAR teach*) or denver or leap or (learning experiences W2 alternative program) or "Computer-Assisted Instruction" or (assist* W tech*) )

S16 ( "Early Intervention" or (computer W2 teach*) or (computer w2 instruct*) ) or ( (neurodevelop* W2 treat*) or ndt or walden or adlerian or theraplay or Eden or (social W2 pragmatic) )

S15 ( "early bird" or (video W2 model*) ) or self W2 manage* or self w2 monitor*

S14 ( "Skills Training" or ABA ) or applied W1 behavio?ral W1 analy*

S13 ( applied verbal behavio?r or verbal behavio* )

S12 Augment* W3 Communicat* and ( "occupational therapy" or "Computer-Assisted Instruction" ) and assist* W1 tech*

S11 ( yale or bancroft or horizon or “may institute” )

S10 ( Dance or Music or Play or Socioenvironmental ) and Therap*

S9 ( parent* or caregive* or care-giver* or family or families or mother* or father* ) and ( treat* or thera* or interv* or direct* or program* )

S8 verbal W2 communicat* or verbal W2 therap*

S7 ( lovaas or linwood or Douglass or CABAS or DTT )

S6 (Treatment W2 Education W2 Autistic W2 communication W2 Handicapped W2 children)

S5 ( teacch or “floor time” or "Social Communication Emotional Regulation Transactional Support" )

S4 scerts or pivotal W2 response or discrete trial*

S3 ( sensory or auditory ) and ( treat* or therap* ) and integration

S2 ( “Sensory Motor Integration” or “facilitated communication” )

S1 ( Pervasive Development Disorder* or Kanner* or Speech Disorder* or Communication Disorder* or AutiS* or Asperger* or PDD or PDD-NOS )

**Table A16. Linguistics and Language Behavior Abstracts**

**Years/issue searched:** 1973 to 2007

**Search date:** 08 May, 2007

(KW=Pervasive Child Development Disorder* or Kanner* or Speech Disorder* or Communication Disorder* or AutiS* or Asperger or PDD or PDD-NOS or Childhood Disintegrative Disorder* or Childhood Schizophrenia) and (KW=Behavior Therapy or Social Skills Training or applied behavio?ral analy* or ABA or intensive behavio?ral intervent* or (IBI or IBT) or applied verbal behavio?r or verbal behavio* or (verbal NEAR (therap* or communicat*)) or lovaas or linwood or Douglass or CABAS or DTT or (Treatment NEAR Education NEAR Autistic NEAR communication NEAR Handicapped NEAR children) or teacch or floor time or "Social Communication Emotional Regulation Transactional Support" or scerts or (pivotal NEAR response) or discrete trial* or (((sensory or auditory) NEAR integration) and (treat* or therap*)) or Sensory Motor Integration or facilitated communication or Family Therapy or ((parent or parents or caregiver* or care-giver* or family or families or mother* or father* or maternal* or paternal*) NEAR (treat* or therap* or interven* or direct* or program* or train* or mediat* or rehabilit*)) or Picture Exchange or Sensory stimulation or Language Therapy or Speech Therapy or (Alternative NEAR Augmentative NEAR Communication) or . occupational therapy or Computer-Assisted Instruction or (assist* NEAR tech*) or Dance Therapy or Music Therapy or Play Therapy or Socioenvironmental therapy or Early Intervention or (computer NEAR (teach* or instruct*)) or social stories or prompt* or ((augment* or social) NEAR communicat*) or (relationship NEAR develop*) or (cognitive and (treat* or therap* or psychotherap*)) or cbt or (sound NEAR (treat* or therap*)) or (natural NEAR environment) or (activity NEAR schedule*) or (direct NEAR instruct*) or (giant NEAR step*) or developmental individual difference or option or (sonrise or kaufman) or precision or (social NEAR skill*) or hanen or miller or patterning* or philadelphia or (dolman or delaccato) or (echange NEAR developpement) or bartelemy or (gentle NEAR teach*) or denver or leap or (learning experiences NEAR alternative program) or pcdi or “princeton child development institute” or rutgers or (natural NEAR teach*) or milieu or (neurodevelop* NEAR treat*) or ndt or walden or adlerian or theraplay or Eden or (social NEAR pragmatic) or "early bird" or (video NEAR model*) or (self NEAR (manage* or monitor*)) or yale or bancroft or horizon or “may institute”) AND (PT=(book chapter) OR PT=(conference paper) OR PT=(dissertation) OR PT=(journal article))

**Table A17. Psychology and Behavioral Sciences Collection – EBSCO Version**

**Years/issue searched:** 1965 to 2007

**Search date:** 08 May, 2007

S24 ( S23 or S22 or S21 or S20 or S19 or S18 or S17 or S16 or S15 or S14 or S13 or S12 or S11 or S10 or S9 or S8 or S7 or S6 or S5 or S4 or S3 or S2 ) and S1

S23 ( "Behavior Therapy" or IBI or IBT )

S22 ( precision or (social W2 skill*) or hanen or miller or patterning* or philadelphia or (dolman W2 delaccato) )

S21 ( "social stories" or prompt* or (social W2 communicat*) or (relationship W2 develop*) or (cognitive and (treat* or therap* or psychother*)) )

S20 ( "Picture Exchange" or "Sensory stimulation" or "Language Therapy" or "Speech Therapy" )

S19 ( (direct W2 instruct*) or (giant W2 step*) or developmental individual difference or option or (sonrise or kaufman) )

S18 ( CBT or (sound W2 treat*) or (sound W2 therap*) or (natural W2 environment) or (activity W2 schedule*) or (Augment* W3 Communicat*) OR "occupational therapy" or "Computer-Assisted Instruction" or (assist* W tech*) )

S17 ( pcdi or "princeton child development institute" or rutgers or (natural W2 teach*) or milieu or (echange W2 developpement) or bartelemy or (gentle NEAR teach*) or denver or leap or (learning experiences W2 alternative program) or "Computer-Assisted Instruction" or (assist* W tech*) )

S16 ( "Early Intervention" or (computer W2 teach*) or (computer w2 instruct*) ) or ( (neurodevelop* W2 treat*) or ndt or walden or adlerian or theraplay or Eden or (social W2 pragmatic) )

S15 ( "early bird" or (video W2 model*) ) or self W2 manage* or self w2 monitor*

S14 ( "Skills Training" or ABA ) or applied W1 behavio?ral W1 analy*

S13 ( applied verbal behavio?r or verbal behavio* )

S12 Augment* W3 Communicat* and ( "occupational therapy" or "Computer-Assisted Instruction" ) and assist* W1 tech*

S11 ( yale or bancroft or horizon or “may institute” )

S10 ( Dance or Music or Play or Socioenvironmental ) and Therap*

S9 ( parent* or caregive* or care-giver* or family or families or mother* or father* ) and ( treat* or thera* or interv* or direct* or program* )

S8 verbal W2 communicat* or verbal W2 therap*

S7 ( lovaas or linwood or Douglass or CABAS or DTT )

S6 (Treatment W2 Education W2 Autistic W2 communication W2 Handicapped W2 children)

S5 ( teacch or “floor time” or "Social Communication Emotional Regulation Transactional Support" )

S4 scerts or pivotal W2 response or discrete trial*

S3 ( sensory or auditory ) and ( treat* or therap* ) and integration

S2 ( “Sensory Motor Integration” or “facilitated communication” )

S1 ( Pervasive Development Disorder* or Kanner* or Speech Disorder* or Communication Disorder* or Autis* or Asperger* or PDD or PDD-NOS )

**Table A18. Cochrane Database of Systematic Reviews, Database of Abstracts of Reviews of Effects, Heath Technology Assessment Database, NHS Economic Evaluation Database – Wiley Version**

**Years/issue searched:** 2nd Quarter 2007

**Search date:** 10 May, 2007

#1 ("pervasive development" NEAR disorder*) or kanner* or speech disorder* or communication disorder* or autis* or asperger or pdd or pdd-nos or childhood disintegrative disorder* or "childhood schizophrenia" in Title, Abstract or Keywords and "behavior therapy" or "social skills training" or applied behavio?ral analy* or aba or intensive behavio?ral intervent* or (ibi or ibt) or applied verbal behavio?r or verbal behavio* or (verbal NEAR (therap* or communicat*)) or lovaas or linwood or douglass or cabas or dtt or (treatment NEAR education NEAR autistic NEAR communication NEAR handicapped NEAR children) or teacch or floor time or "social communication emotional regulation transactional support" or scerts or (pivotal NEAR response) or discrete trial* or (((sensory or auditory) NEAR integration) and (treat* or therap*)) or "sensory motor integration" or "facilitated communication" in Title, Abstract or Keywords in all products

#2 ("pervasive development" NEAR disorder*) or kanner* or speech disorder* or communication disorder* or autis* or asperger or pdd or pdd-nos or childhood disintegrative disorder* or "childhood schizophrenia" in All Fields and "family therapy" or ((parent or parents or caregiver* or care-giver* or family or families or mother* or father* or maternal* or paternal*) NEAR (treat* or therap* or interven* or direct* or program* or train* or mediat* or rehabilit*)) or "picture exchange" or "sensory stimulation" or "language therapy" or "speech therapy" or (alternative NEAR augmentative NEAR communication) or "occupational therapy" or "computer-assisted instruction" or (assist* NEAR tech*) or "dance therapy" or "music therapy" or "play therapy" or "socioenvironmental therapy" or "early intervention" or (computer NEAR (teach* or instruct*)) or "social stories" or prompt* or ((augment* or social) NEAR communicat*) in Title, Abstract or Keywords in all products

#3 ("pervasive development" NEAR disorder*) or kanner* or speech disorder* or communication disorder* or autis* or asperger or pdd or pdd-nos or childhood disintegrative disorder* or "childhood schizophrenia" in Title, Abstract or Keywords and (relationship NEAR develop*) or (cognitive and (treat* or therap* or psychotherap*)) or cbt or (sound NEAR (treat* or therap*)) or (natural NEAR environment) or (activity NEAR schedule*) or (direct NEAR instruct*) or (giant NEAR step*) or "developmental individual difference" or option or (sonrise or kaufman) or precision or (social NEAR skill*) or hanen or miller or patterning* or philadelphia or (dolman or delaccato) or (echange NEAR developpement) or bartelemy or (gentle NEAR teach*) or denver or leap or (learning experiences NEAR alternative program) or pcdi or "princeton child development institute" or rutgers or (natural NEAR teach*) or milieu or (neurodevelop* NEAR treat*) or ndt or walden or adlerian or theraplay or eden or (social NEAR pragmatic) or "early bird" or (video NEAR model*) or (self NEAR (manage* or monitor*)) or yale or bancroft or horizon or "may institute" in Title, Abstract or Keywords in all products

#4 (#1 OR #2 OR #3)

**Table A19. OCLC Papers First and OCLC Proceedings First – OCLC FirstSearch**

**Years/issue searched:** 1993 to 2007

**Search date:** 09 May, 2007

(kw: Pervasive W Development or kw: Kanner* or kw: Speech w Disorder* or kw: Communication w Disorder* or kw: Autis* or kw: Asperger* or kw: PDD or kw: PDD-NOS or kw: Disintegrative w Disorder* or kw: Childhood w Schizophrenia) and ((kw: Behavior W Therapy) or (kw: Social W Skills W Training) or (kw: applied W behavio?ral W analy*) or kw: ABA or (kw: intensive W behavio?ral and kw: intervent*) or (kw: IBI or kw: IBT) or (kw: applied W verbal W behavio?r) or (kw: verbal W behavio*) or (kw: verbal W (therap* or kw: communicat*)) or kw: lovaas or kw: linwood or kw: Douglass or kw: CABAS or kw: DTT or (kw: Treatment W Education W Autistic W communication W Handicapped W children) or kw: teacch or (kw: floor w time) or (kw: Social W Communication W Emotional and kw: Regulation W Transactional W Support) or kw: scerts or (kw: pivotal W response) or (kw: discrete W trial*) or ((kw: sensory or kw: auditory) W integration) or (kw: Sensory W Motor W Integration) or (kw: facilitated W communication) or (kw: Family W Therapy) or ((kw: parent or kw: parents or kw: caregiver* or kw: care-giver* or kw: family or kw: families or kw: mother* or kw: father* or kw: maternal* or kw: paternal*) W (treat* or kw: therap* or kw: interven* or kw: direct* or kw: program* or kw: train* or kw: mediat* or kw: rehabilit*)) or (kw: Picture W Exchange) or ((kw: Sensory W stimulation) or (kw: Language W Therapy) or (kw: Speech W Therapy) or (kw: Alternative W Augmentative W Communication) or (kw: occupational W therapy) or (kw: Computer-Assisted W Instruction) or (kw: assistive* W technolog*) or (kw: Dance W Therapy) or (kw: Music W Therapy) or (kw: Play W Therapy) or (kw: Socioenvironmental W therapy) or (kw: Early W Intervention) or (kw: computer W (teach* or kw: instruct*)) or (kw: social W stories) or kw: prompt OR kw: prompting or ((kw: augment* or kw: social) W communicat*)) or ((kw: relationship W development*) or (kw: cognitive and (kw: treatment* or kw: therap* or kw: psychotherap*)) or kw: cbt or (kw: sound W (treatment* or kw: therap*)) or (kw: natural W environment) or (kw: activity W schedule*) or (kw: direct W instruct*) or (kw: giant W step*)) or ((kw: developmental W individual W difference) or kw: option or (kw: sonrise or kw: kaufman) or kw: precision or (kw: social W skill*) or kw: hanen or kw: miller or kw: patterning* or kw: philadelphia or (kw: dolman or kw: delaccato) or (kw: echange W developpement) or kw: bartelemy or (kw: gentle W teach*)) or (kw: denver or kw: leap or (kw: learning W experiences W alternative W program*) or kw: pcdi or (kw: princeton W child W development W institute) or kw: rutgers or (kw: natural W teach*) or kw: milieu) or ((kw: neurodevelopment* W treatment*) or kw: ndt or kw: walden or kw: adlerian or kw: theraplay or kw: Eden or (kw: social W pragmatic) or (kw: early W bird) or (kw: video W modelling) or (kw: self W (manage* or kw: monitor*)) or kw: yale or kw: bancroft or kw: horizon or (kw: may W institute)))

(ti: Pervasive W Development or ti: Kanner* or ti: Speech w Disorder* or ti: Communication w Disorder* or ti: Autis* or ti: Asperger* or ti: PDD or ti: PDD-NOS or ti: Disintegrative w Disorder* or ti: Childhood w Schizophrenia) and ((ti: Behavior W Therapy) or (ti: Social W Skills W Training) or (ti: applied W behavio?ral W analy*) or ti: ABA or (ti: intensive W behavio?ral and ti: intervent*) or (ti: IBI or ti: IBT) or (ti: applied W verbal W behavio?r) or (ti: verbal W behavio*) or (ti: verbal W (therap* or ti: communicat*)) or ti: lovaas or ti: linwood or ti: Douglass or ti: CABAS or ti: DTT or (ti: Treatment W Education W Autistic W communication W Handicapped W children) or ti: teacch or (ti: floor w time) or (ti: Social W Communication W Emotional and ti: Regulation W Transactional W Support) or ti: scerts or (ti: pivotal W response) or (ti: discrete W trial*) or ((ti: sensory or ti: auditory) W integration) or (ti: Sensory W Motor W Integration) or (ti: facilitated W communication) or (ti: Family W Therapy) or ((ti: parent or ti: parents or ti: caregiver* or ti: care-giver* or ti: family or ti: families or ti: mother* or ti: father* or ti: maternal* or ti: paternal*) W (treat* or ti: therap* or ti: interven* or ti: direct* or ti: program* or ti: train* or ti: mediat* or ti: rehabilit*)) or (ti: Picture W Exchange) or ((ti: Sensory W stimulation) or (ti: Language W Therapy) or (ti: Speech W Therapy) or (ti: Alternative W Augmentative W Communication) or (ti: occupational W therapy) or (ti: Computer-Assisted W Instruction) or (ti: assistive* W technolog*) or (ti: Dance W Therapy) or (ti: Music W Therapy) or (ti: Play W Therapy) or (ti: Socioenvironmental W therapy) or (ti: Early W Intervention) or (ti: computer W (teach* or ti: instruct*)) or (ti: social W stories) or ti: prompt OR ti: prompting or ((ti: augment* or ti: social) W communicat*)) or ((ti: relationship W development*) or (ti: cognitive and (ti: treatment* or ti: therap* or ti: psychotherap*)) or ti: cbt or (ti: sound W (treatment* or ti: therap*)) or (ti: natural W environment) or (ti: activity W schedule*) or (ti: direct W instruct*) or (ti: giant W step*)) or ((ti: developmental W individual W difference) or ti: option or (ti: sonrise or ti: kaufman) or ti: precision or (ti: social W skill*) or ti: hanen or ti: miller or ti: patterning* or ti: philadelphia or (ti: dolman or ti: delaccato) or (ti: echange W developpement) or ti: bartelemy or (ti: gentle W teach*)) or (ti: denver or ti: leap or (ti: learning W experiences W alternative W program*) or ti: pcdi or (ti: princeton W child W development W institute) or ti: rutgers or (ti: natural W teach*) or ti: milieu) or ((ti: neurodevelopment* W treatment*) or ti: ndt or ti: walden or ti: adlerian or ti: theraplay or ti: Eden or (ti: social W pragmatic) or (ti: early W bird) or (ti: video W modelling) or (ti: self W (manage* or ti: monitor*)) or ti: yale or ti: bancroft or ti: horizon or (ti: may W institute)))

**Table A20. Dissertation Abstracts**

**Years/issue searched:** 1931 to 2007

**Search date:** 09 May, 2007

Pervasive Development Disorder* or Kanner* or Speech Disorder* or Communication Disorder* or AutiS* or Asperger* or PDD or PDD-NOS

("Behavior Therapy" or IBI or IBT)

precision or (social W/2 skill*) or hanen or miller or patterning* or philadelphia or (dolman W/2 delaccato)

"social stories" or prompt* or (social W/2 communicat*) or (relationship W/2 develop*) or (cognitive and (treat* or therap* or psychother*))

"Picture Exchange" or "Sensory stimulation" or "Language Therapy" or "Speech Therapy"

(direct W/2 instruct*) or (giant W/2 step*) or developmental individual difference or option or (sonrise or kaufman)

(CBT or (sound W/2 (treat* or therap*)) or (natural W/2 environment) or (activity W/2 schedule*))

(Augment* W/3 Communicat*) OR "occupational therapy" or "Computer-Assisted Instruction" or (assist* W/1 tech*)

(pcdi or "princeton child development institute" or rutgers or (natural W/2 teach*) or milieu)

((echange W/2 developpement) or bartelemy or (gentle NEAR teach*) or denver or leap or (learning experiences W/2 alternative program))

"Early Intervention" or (computer w/2 (teach* or instruct*))

((neurodevelop* W/2 treat*) or ndt or walden or adlerian or theraplay or Eden or (social W/2 pragmatic))

("early bird" or (video W/2 model*) or (self W/2 (manage* or monitor*))

Skills Training or applied behavio?ral analy* or ABA)

(applied verbal behavio?r or verbal behavio*)

((Dance or Music or Play or Socioenvironmental) w/1 Therapy)

((Augment* W/3 Communicat*) OR "occupational therapy" or "Computer-Assisted Instruction" or (assist* W/1 tech*))

yale or bancroft or horizon or “may institute”

(((parent* or caregive* or care-giver* or family or families or mother* or father*) W/2 (treat* or thera* or interv* or direct* or program*)))

verbal W/2 (therap* or communicat*)) or lovaas or linwood or Douglass or CABAS or DTT

(Treatment W/2 Education W/2 Autistic W/2 communication W/2 Handicapped W/2 children) or

teacch or “floor time” or "Social Communication Emotional Regulation Transactional Support"

scerts or (pivotal W/2 response) or discrete trial* or (((sensory or auditory) W/2 integration) and (treat* or therap*)) or

“Sensory Motor Integration” or “facilitated communication”

**Table A21. LILACS – OCLC FirstSearch**

**Years/issue searched:** 1982 to 2007

**Search date:** 09 May, 2007

(kw: Pervasive W Development or kw: Kanner* or kw: Speech w Disorder* or kw: Communication w Disorder* or kw: Autis* or kw: Asperger* or kw: PDD or kw: PDD-NOS or kw: Disintegrative w Disorder* or kw: Childhood w Schizophrenia) and ((kw: Behavior W Therapy) or (kw: Social W Skills W Training) or (kw: applied W behavio?ral W analy*) or kw: ABA or (kw: intensive W behavio?ral and kw: intervent*) or (kw: IBI or kw: IBT) or (kw: applied W verbal W behavio?r) or (kw: verbal W behavio*) or (kw: verbal W (therap* or kw: communicat*)) or kw: lovaas or kw: linwood or kw: Douglass or kw: CABAS or kw: DTT or (kw: Treatment W Education W Autistic W communication W Handicapped W children) or kw: teacch or (kw: floor w time) or (kw: Social W Communication W Emotional and kw: Regulation W Transactional W Support) or kw: scerts or (kw: pivotal W response) or (kw: discrete W trial*) or ((kw: sensory or kw: auditory) W integration) or (kw: Sensory W Motor W Integration) or (kw: facilitated W communication) or (kw: Family W Therapy) or ((kw: parent or kw: parents or kw: caregiver* or kw: care-giver* or kw: family or kw: families or kw: mother* or kw: father* or kw: maternal* or kw: paternal*) W (treat* or kw: therap* or kw: interven* or kw: direct* or kw: program* or kw: train* or kw: mediat* or kw: rehabilit*)) or (kw: Picture W Exchange) or ((kw: Sensory W stimulation) or (kw: Language W Therapy) or (kw: Speech W Therapy) or (kw: Alternative W Augmentative W Communication) or (kw: occupational W therapy) or (kw: Computer-Assisted W Instruction) or (kw: assistive* W technolog*) or (kw: Dance W Therapy) or (kw: Music W Therapy) or (kw: Play W Therapy) or (kw: Socioenvironmental W therapy) or (kw: Early W Intervention) or (kw: computer W (teach* or kw: instruct*)) or (kw: social W stories) or kw: prompt OR kw: prompting or ((kw: augment* or kw: social) W communicat*)) or ((kw: relationship W development*) or (kw: cognitive and (kw: treatment* or kw: therap* or kw: psychotherap*)) or kw: cbt or (kw: sound W (treatment* or kw: therap*)) or (kw: natural W environment) or (kw: activity W schedule*) or (kw: direct W instruct*) or (kw: giant W step*)) or ((kw: developmental W individual W difference) or kw: option or (kw: sonrise or kw: kaufman) or kw: precision or (kw: social W skill*) or kw: hanen or kw: miller or kw: patterning* or kw: philadelphia or (kw: dolman or kw: delaccato) or (kw: echange W developpement) or kw: bartelemy or (kw: gentle W teach*)) or (kw: denver or kw: leap or (kw: learning W experiences W alternative W program*) or kw: pcdi or (kw: princeton W child W development W institute) or kw: rutgers or (kw: natural W teach*) or kw: milieu) or ((kw: neurodevelopment* W treatment*) or kw: ndt or kw: walden or kw: adlerian or kw: theraplay or kw: Eden or (kw: social W pragmatic) or (kw: early W bird) or (kw: video W modelling) or (kw: self W (manage* or kw: monitor*)) or kw: yale or kw: bancroft or kw: horizon or (kw: may W institute)))  and li: LILAC

(ti: Pervasive W Development or ti: Kanner* or ti: Speech w Disorder* or ti: Communication w Disorder* or ti: Autis* or ti: Asperger* or ti: PDD or ti: PDD-NOS or ti: Disintegrative w Disorder* or ti: Childhood w Schizophrenia) and ((ti: Behavior W Therapy) or (ti: Social W Skills W Training) or (ti: applied W behavio?ral W analy*) or ti: ABA or (ti: intensive W behavio?ral and ti: intervent*) or (ti: IBI or ti: IBT) or (ti: applied W verbal W behavio?r) or (ti: verbal W behavio*) or (ti: verbal W (therap* or ti: communicat*)) or ti: lovaas or ti: linwood or ti: Douglass or ti: CABAS or ti: DTT or (ti: Treatment W Education W Autistic W communication W Handicapped W children) or ti: teacch or (ti: floor w time) or (ti: Social W Communication W Emotional and ti: Regulation W Transactional W Support) or ti: scerts or (ti: pivotal W response) or (ti: discrete W trial*) or ((ti: sensory or ti: auditory) W integration) or (ti: Sensory W Motor W Integration) or (ti: facilitated W communication) or (ti: Family W Therapy) or ((ti: parent or ti: parents or ti: caregiver* or ti: care-giver* or ti: family or ti: families or ti: mother* or ti: father* or ti: maternal* or ti: paternal*) W (treat* or ti: therap* or ti: interven* or ti: direct* or ti: program* or ti: train* or ti: mediat* or ti: rehabilit*)) or (ti: Picture W Exchange) or ((ti: Sensory W stimulation) or (ti: Language W Therapy) or (ti: Speech W Therapy) or (ti: Alternative W Augmentative W Communication) or (ti: occupational W therapy) or (ti: Computer-Assisted W Instruction) or (ti: assistive* W technolog*) or (ti: Dance W Therapy) or (ti: Music W Therapy) or (ti: Play W Therapy) or (ti: Socioenvironmental W therapy) or (ti: Early W Intervention) or (ti: computer W (teach* or ti: instruct*)) or (ti: social W stories) or ti: prompt OR ti: prompting or ((ti: augment* or ti: social) W communicat*)) or ((ti: relationship W development*) or (ti: cognitive and (ti: treatment* or ti: therap* or ti: psychotherap*)) or ti: cbt or (ti: sound W (treatment* or ti: therap*)) or (ti: natural W environment) or (ti: activity W schedule*) or (ti: direct W instruct*) or (ti: giant W step*)) or ((ti: developmental W individual W difference) or ti: option or (ti: sonrise or ti: kaufman) or ti: precision or (ti: social W skill*) or ti: hanen or ti: miller or ti: patterning* or ti: philadelphia or (ti: dolman or ti: delaccato) or (ti: echange W developpement) or ti: bartelemy or (ti: gentle W teach*)) or (ti: denver or ti: leap or (ti: learning W experiences W alternative W program*) or ti: pcdi or (ti: princeton W child W development W institute) or ti: rutgers or (ti: natural W teach*) or ti: milieu) or ((ti: neurodevelopment* W treatment*) or ti: ndt or ti: walden or ti: adlerian or ti: theraplay or ti: Eden or (ti: social W pragmatic) or (ti: early W bird) or (ti: video W modelling) or (ti: self W (manage* or ti: monitor*)) or ti: yale or ti: bancroft or ti: horizon or (ti: may W institute))) and li: LILAC

**Table A22. NLM Gateway – National Library of Medicine**

**Years/issue searched:** 1950 to 2007

**Search date:** 10 May, 2007

Kanners OR Autistic OR autism OR Aspergers or PDD or "PDD-NOS" or "Childhood Disintegrative Disorder" OR Childhood Schizophrenia[MESH] OR Autistic Disorder[MESH] OR Child Development Disorders, Pervasive[MESH] OR Communication Disorders[MESH] OR Asperger Syndrome[MESH] OR Speech Disorders[MESH]

**Table A23. DOAJ - Directory of Open Access Journals**

**Years/Issue Searched:** 1977 to 2007

**Search date:** 11 May, 2007

Autism or autistic
