# Supplementary material for: Behavioural and Developmental Interventions for Autism Spectrum Disorder: A Clinical Systematic Review
Source: PLoS One. 2008 Nov 18;3(11):e3755. doi: 10.1371/journal.pone.0003755 (PMC2582449; doi:10.1371/journal.pone.0003755)
Supplement: Supplement S2 — List of excluded studies (0.39 MB DOC) [file pone.0003755.s002.doc]

**Supplement B. Excluded Studies and Non-Obtained Studies**

One thousand and twenty eight studies were excluded. The reasons for exclusion are as follows: (1) the study was not primary research on ASD (n= 83), (2) the study did not assess a cognitive/behavioural intervention for ASD (n= 15), (3) the study did not report adequately on any measurable data for health related outcomes relevant to the review (n= 101), (4) the study did not examine an ASD population (n= 46), and (5) the study design was not an RCT or an observational analytical cohort study (n= 783).

**Excluded – Not primary research on ASD (N = 83)**

The following studies were excluded because they were not primary research on ASD.

1. Keep your eye on .Lego building blocks to teach social skills in autistic children. Brown Unive Child Adolesc Behav Lett 2006;22(10):2.

2. Aldred C, Stockport NHS Foundation Trust. Child's talk: intensive communication and interactive therapy for children with autism. A pilot study to measure outcome of therapy: do the social communication skills of an intervened group of autistic children differ significantly from a non-intervened group and are the skills maintained 6 months post-invention? Oxford (UK): Steve Shirley Foundation, 2000.

3. Aldred C, Pollard C, Phillips R, et al. Multidisciplinary social communication intervention for children with autism and pervasive developmental disorder: the Child's Talk project. Educ Child Psychol 2001;18(2):76-87.

4. Attwood T. Cognitive behaviour therapy for children and adults with Asperger's syndrome. Behav Change 2004;21(3):147-61.

5. Baker BS. The use of music with autistic children. J Psychos Nurs 1982;20(4):31-4.

6. Barnett A. Misapplications reviews: dealing with autism. Interfaces 1989;19(3):27-32.

7. Bell CM. Music therapy with autistic spectrum disorder . Bazian Ltd (Editors): Wessex Institute for Health Research and Development, University of Southampton, 2003.

8. Biklen D, Schubert A. New words: the communication of students with autism. Rem Spec Educ 1991;12(6):46-57.

9. Billard A, Robins B, Nadel J, et al. Building Robota, a mini-humanoid robot for the rehabilitation of children with autism. Assist Technol 2007;19(1):37-49.

10. Bloom B. A descriptive study of early childhood intervention programs in Saskatchewan final report of "the alpern-boll" data, 1984-1990. Saskatoon, SK: Department of Social Services, Community Living Division, Regina, SK and Saskatchewan Association for Community Living, Saskatoon, SK, 1991.

11. Bottroff V, Zeitz J. A model of support and intervention for individuals with Asperger syndrome and their families. J Intell Disabil Res 2004;48:445.

12. Brinton B, Fujiki M. Social competence in children with language impairment: making connections. Semin Speech Lang 2005;26(3):151-9.

13. Burrows K. Early intervention for children with autism. Ped Child Health 2004;9(4):267-70.

14. Buschbacher PW, Fox L. Understanding and intervening with the challenging behavior of young children with autism spectrum disorder. Lang Speech Hear Serv Sch 2003;34(3):217-27.

15. Butter EM, Wynn J, Mulick JA. Early intervention critical to autism treatment. Pediatr Ann 2003;32(10):677-84.

16. Chapman L, Trowbridge M. Social stories for reducing fear in the outdoors. Horizons 2000;121:38-40.

17. Charman T, Howlin P, Aldred C, et al. Research into early intervention for children with autism and related disorders: methodological and design issues: report on a workshop funded by the Wellcome Trust, Institute of Child Health, London, UK, November 2001. Autism 2003;7(2):217-25.

18. Dillenburger K, Keenan M, Gallagher S, et al. Autism: intervention and parental empowerment. Child Care Pract 2002;8(3):216-9.

19. Dimond C, Hyde C. Parent education programmes for children's behaviour problems. Medium to long term effectiveness (Structured abstract) [web page]. 2007; Available at: http://www.mrw.interscience.wiley.com/cochrane/cldare/articles/DARE-20008583/frame.html. Accessed May, 2007.

20. Fennick E, Royle J. Community inclusion for children and youth with developmental disabilities. Focus Autism Other Dev Disabil 2003;18(1):20.

21. Ferritor DE. Modifying interaction patterns: an experimental training program for parents of autistic children [dissertation]. Seattle (WA): University of Washington; 1969.

22. Finn P, Bothe AK, Bramlett RE. Science and pseudoscience in communication disorders: criteria and applications. Am J Speech Lang Pat 2005;14(3):172-86.

23. Foley BE, Staples AH. Developing Augmentative and Alternative Communication (AAC) and literacy interventions in a supported employment setting. Top Lang Disord 2003;23(4):325-43.

24. Fuentes J, Gallano I, Gallano I. Applying TEACCH in developing autism services in Spain: the Gautena project. Int J Ment Health 2000;29(2):78-88.

25. Fuggle K, Fixter A, Brown SW. Music therapy in the treatment of a child with epilepsy and autism. Epilepsia 1995;36(Suppl. 3):S74.

26. Green VA, Pituch KA, Itchon J, et al. Internet survey of treatments used by parents of children with autism. Res Dev Disabil 2006;27(1):70-84.

27. Handleman JS, Harris SL. Douglass Developmental Disabilities Center: An ABA program for children and adults with autism spectrum disorders. Int J Behav Consult Ther 2005;1(4):301-11.

28. Handleman JS, Harris SL, Kristoff B, et al. A specialized program for preschool children with autism. Lang Speech Hear Serv Sch 1991;22(3):107-10.

29. Harris SL. Involving college students and parents in a child care setting: a day school for the child with autistic behaviors. Child Care Q 1974;3(3):188-94.

30. Harris SL, Delmolino L. Applied behavior analysis: its application in the treatment of autism and related disorders in young children. Infant Young Child 2002;14(3):11-7.

31. Hirsch RF. Auditory verbal and visual manual sign decoding of verbal and nonverbal autistic children: a study of cross-modality versus within-modality processing [dissertation]. Los Angeles (CA): University of Southern California; 1973.

32. Howlin P. Reaching the autistic child: a parent training program. Behav Res Ther 1974;12(4):362-3.

33. Ingenmey R. Delayed prompts promote spontaneous speech in an autistic child during play [dissertation]. Halifax (NS): Mount Saint Vincent University; 1989.

34. Kampa A, Kennedy J, Velde B, et al. The Friendship Club: developing reciprocal relationships in children with Asperger's syndrome. OT Practice 2003;8(19):25-7.

35. Kasari C, Freeman S, Paparella T. Interventions targeting joint attention and symbolic play can improve aspects of these skills in young children with autism. EBMH 2007;10 (1):21.

36. Kasari C, Freeman S, Paparella T, et al. Early intervention on core deficits in autism. Clin Neuropsych 2005;2(6):380-8.

37. Kasari C, Freeman SFN, Paparella T. Early intervention in autism: joint attention and symbolic play.Glidden LM, editor. International Review of Research in Mental Retardation. 23. San Diego: Academic Press Inc; 2001:207-37.

38. Kubina RM Jr, Morrison R, Lee DL. Benefits of adding precision teaching to behavioral interventions for students with autism . Behav Intervent 2002;17(4):233-46.

39. Le Couteur A, Sowter M. Early assessment and intervention in autism: adapting the family focussed Hanen Parent Programme for pre-school children with autism spectrum disorders. 1997 Conference proceediongs: Living and learning with autism: perspectives from the individual, the family and the professional; Durham, UK. Autism Research Unit; 1997. p. 29-42.

40. Leroy G, Chuang S, Huang J, et al. Digital libraries on handhelds for autistic children. Proceedings of the 5th ACM/IEEE-CS joint conference on Digital libraries; Denver, CO. New York, (NY): ACM Press; 2005. p. 387.

41. Levinge A. 'The use of I and me': music therapy with an autistic child. J Br Music Ther 1990;4(2):15-8.

42. Levy S, Kim A, Olive ML. Interventions for young children with autism: a synthesis of the literature. Focus Autism Other Dev Disabil 2006;21(1):55-62.

43. Light JC, Roberts B, Dimarco R, et al. Augmentative and alternative communication to support receptive and expressive communication for people with autism. J Commun Disord 1998;31(2):153-80.

44. Lord C, Wagner A, Rogers S, et al. Challenges in evaluating psychosocial interventions for autistic spectrum disorders. J Autism Dev Disord 2005;35(6):695-708; discussion 709-11.

45. Lovaas OI, Smith T.Thompson T, Gray DB, editors. Destructive behavior in developmental disabilities: diagnosis and treatment. Thousand Oaks: Sage Publications, Inc; 1994:243-60.

46. Lowery EF. Autistic aloofness reconsidered: case reports of two children in play therapy. Bull Menninger Clin 1985;49(2):135-50.

47. Magerotte G, Roge B. Family quality of life and early intervention in autism (Auti-Qol). J Intell Disabil Res 2004;48:328.

48. Maley A, Maybery M. The effectiveness of early intervention treatment for children with autism spectrum disorders. Aust J Psychol 2003;55(Suppl. S):194.

49. Marriage KJ, Gordon V, Brand L. A social skills group for boys with Asperger's syndrome. Aust NZ J Psychiatr 1995;29(1):58-62.

50. Mastropieri MA, Scruggs TE. Early intervention for socially withdrawn children. J Spec Educ 1985;19(4):429-41.

51. McClannahan LE, MacDuff GS, Krantz PJ. Behavior analysis and intervention for adults with autism. Behav Modif 2002;26(1):9-26.

52. McConachie H. Joint attention and symbolic play in young children with autism. Child Care Hlth Dev 2006;32(6):752.

53. Means JR, Merrens MR. Interpersonal training for an autistic child. Percept Motor Skills 1969;28(3):972-4.

54. Mesibov GB, Browder DA, Kirkland C. Using individualized schedules as a component of positive behavioral support for students with developmental disabilities. J Pos Behav Interv 2002;4(2):73-9.

55. Miller EP. Language acquisition training for parents of autistic children [dissertation]. Louisville (KT): University of Louisville; 1981.

56. Oliver CJ. Triage of the autistic spectrum child utilizing the congruence of case management concepts and Orem's nursing theories. Lippincott's Case Manage 2003;8(2):66-82.

57. Paisley DD, Cahill F. An analysis of how a secure environment for adults with autistic spectrum disorder and challenging behaviour provides and applies the TEACCH philosophy within the restrictions created by detention under the Mental Health Act 1983. 11th Durham Conference on Autism; Durham, UK. Sunderland: Autism Research Unit; 2000. p. 33-42.

58. Park D. Operant conditioning of a speaking autistic child. J Autism Child Schiz 1974;4(2):189-91.

59. Quill K, Gurry S, Larkin A. Daily life therapy: a Japanese model for educating children with autism. J Autism Dev Disord 1989;19(4):625-35.

60. Quill KA. Visually cued instruction for children with autism and pervasive developmental disorders. Focus Autistic Behav 1995;10(3):10-20.

61. Quinn S. Winnipeg's elementary autistic option: an integrated cluster program. International conference: Autism: a world of options; Toronto; Canada. Arlington (TX): Future Education, Inc; 1993. p. 101-4.

62. Rathkey JH, Krug DA, Flax SW, et al. A microprocessor-based aid for training autistic children. Biomed Sci Instrum 1978;14:87-96.

63. Rowe C. The Stanley Segal award: do social stories benefit children with autism in mainstream primary schools? Br J Special Educ 1999;26(1):12-4.

64. Rutter M, Sussenwein F. A developmental and behavioral approach to the treatment of preschool autistic children. J Autism Child Schiz 1971;1(4):376-97.

65. Sasaki M. Aspects of autism in Japan before and after the introduction of TEACCH. Int J Ment Health 2000;29(2):3-18.

66. Schoen SF, Bullard M. Action research during recess: a time for children with autism to play and learn. Teach Except Child 2002;35(1):36-9.

67. Schuler A, Gonsier-Gerdin J, Wolfberg P. The efficacy of speech and language intervention: autism. Semin Speech Lang 1990;11(4):242-55.

68. Schulz M. Socializing influence of remedial educational vaulting on children with autistic attitudes (Asperger syndrome). Proceedings of 9th the International Therapeutic Riding Congress; Denver; CO. Denver: North American Riding for the Handicapped Association; 1997. p. 65-73.

69. Shapiro T, Hertzig M. Applied behavioral analysis: astonishing results? J Am Acad Child Adolesc Psychiatry 1995;34(10):1255-6.

70. Shields J. The NAS earlybird programme: autism-specific early intervention for parents. Prof Care Mother Child 2000;10(2):53-4.

71. Shields J. The NAS earlybird programme: partnership with parents in early intervention. Autism 2001;5(1):49-56.

72. Smith T, Lovaas OI. Intensive and early behavioral intervention with autism: the UCLA young autism project. Infant Young Child 1998;10(3):67-78.

73. Temple K. A randomized comparison of the effect of two prelinguistic communication interventions on the acquisition of spoken communication in preschoolers with ASD. Child Care Hlth Dev 2007;33(3):348-9.

74. Urry N. Behaviour modification with an autistic child. Nurs Times 1970;66(15):456-8.

75. Van Berckelaer-Onnes IA. Promoting early play. Autism 2003;7(4):415-23.

76. Wallen M, Bulkeley K. Three sessions of adult imitation increased some appropriate social behaviours of young children with autism. Aust Occup Ther J 2006;53 ( 2):139-40.

77. Wallen M, Stagnitti K. There was insufficient evidence to conclude whether parent-mediated early intervention was effective for children with autism. Aust Occup Ther J 2006;53(2):137-9.

78. Wallis C, Goehner AL. A tale of two schools. Time 2006;167 ( 20):48-51.

79. Watling R, Tomchek S, LaVesser P, et al. The scope of occupational therapy services for individuals with autism spectrum disorders across the lifespan. Am J Occup Ther 2005;59(6):680-3.

80. White AH. Cognitive behavioural therapy in children with autistic spectrum disorder. Bazian Ltd (editors). Wessex Institute for Health Research and Development, University of Southampton, 2004.

81. Williams EU. Rural necessity as the mother of invention: using collaboration to extend services for autism and low-incidence handicaps. Montgomery D, editor. Rural America: where all innovations begin; Savannah, GA.; 1993.

82. Williams TI. Effects of treatment strategies on the learning and development of autistic children [dissertation]. Dissert Abst Int 1988;49(4A):722.

83. Wing L. Management of early childhood autism. Br J Hosp Med 1981;25(4):355-6, 359.

**Excluded – Not on a Cognitive/Behavioural Intervention for ASD (N = 15)**

The following studies were excluded because they did not assess a cognitive/behavioural intervention for ASD.

1. Arick JR, Krug DA. Autistic children: a study of learning characteristics and programming needs. Am J Ment Defic 1978;83(2):200-2.

2. Campbell M, Anderson LT, Meier M, et al. A comparison of haloperidol and behavior therapy and their interaction in autistic children. J Am Acad Child Psychiatry 1978;17(4):640-55.

3. Chong IM. Assessing the differential outcomes procedure with children diagnosed with autism [dissertation]. Dissert Abst Int 2005;66(1B):536.

4. Elder JH, Shankar M, Shuster J, et al. The gluten-free, casein-free diet in autism: results of a preliminary double blind clinical trial. J Autism Dev Dis 2006; 36(3):413-20.

5. Frankel F, Freeman BJ, Ritvo E, et al. The effect of environmental stimulation upon the stereotyped behavior of autistic children. J Autism Child Schiz 1978;8(4):389-94.

6. Frankel F, Myatt R, Feinberg D. Parent-assisted friendship training for children with autism. Child Psychiat Hum D 2007;37 (4):337-46.

7. Graff RB, Green G, Libby ME. Effects of two levels of treatment intensity on a young child with severe disabilities. Behav Intervent 1998;13(1):21-41.

8. Holttum JR, Lubetsky MJ, Eastman LE. Comprehensive management of trichotillomania in a young autistic girl. J Am Acad Child Adolesc Psychiatry 1994;33(4):577-81.

9. Kerrin RG, Murdock JY, Sharpton WR, et al. Who's doing the pointing? Investigating facilitated communication in a classroom setting with students with autism. Focus Autism Other Dev Disabil 1998;13(2):73-9.

10. Konstantareas MM, Gravelle G. Facilitated communication. Autism 1998;2(4):389-414.

11. Makita K, Umezu K. An objective evaluation technique for autistic children: an introduction of CLAC scheme. Acta Paedopsychiatr 1973;39(8):237-53.

12. Oswald DP. Facilitator influence in facilitated communication. J Behav Educ 1994;4(2):191-9.

13. Rehfeldt RA, Chambers MR. Functional analysis and treatment of verbal perseverations displayed by an adult with autism. J Appl Behav Anal 2003;36(2):259-61 .

14. Runco MA, Schreibman L. Parental judgments of behavior therapy efficacy with autistic children: a social validation. J Autism Dev Disord 1983;13(3):237-48 .

15. Vanestenberg AM. The reduction of autistic symptoms through the utilization of hyperbaric oxygen therapy. Cincinnati (OH): Union Institute and University; 2006.

**Excluded – Outcomes; Inadequate Reporting (N = 101)**

The following study was excluded because data relevant to the outcomes of interest were inadequately reported.

1. Ayres AJ, Tickle LS. Hyper-responsivity to touch and vestibular stimuli as a predictor of positive response to sensory integration procedures by autistic children. Am J Occup Ther 1980;34(6):375-81.

2. Ball J. Increasing social interactions of preschoolers with autism through relationships with typically developing peers [dissertation]. Fort Lauderdale (FL): Nova Southeastern University; 1996.

3. Beadle-Brown J, Murphy G, Dorey H. Evaluation of early intervention in autism: a pilot study. J Intell Disabil Res 2004;48:328.

4. Bernhardt B, Smith D, Smith R. Language intervention with a "family-centered, collaborative, transdisciplinary, integrated" approach: an example. Child Lang Teach Ther 1992;8(3):265-84.

5. Bolte S, Feineis-Matthews S, Leber S, et al. The development and evaluation of a computer-based program to test and to teach the recognition of facial affect. Int J Circumpolar Health 2002;61(Suppl 2):61-8.

6. Boyd BA. Effects of restricted interests on the social behaviors of young children with autism spectrum disorders [dissertation]. Gainesville (FL): University of Florida; 2005.

7. Brereton AV, Tonge BJ, King NK. Autism: a parent-based early intervention. J Intell Disabil Res 2004;48:296.

8. Britten WJ . Preliminary outcomes of a group intervention for families with a child who has a developmental disability. 2003;55:170.

9. Brown MM. Auditory integration training and autism: two case studies. Br J Occup Ther 1999;62(1):13-8.

10. Burke JC. Assessing and training autistic children's responses to cross-modal stimuli containing one to four components [dissertation]. Santa Barbara (CA): University of California; 1987.

11. Cabay M. Brief report: a controlled evaluation of facilitated communication using open-ended and fill-in questions. J Autism Dev Disord 1994;24(4):517-27.

12. Carter C, Meckes L, Pritchard L, et al. The friendship club: an after-school program for children with Asperger syndrome. Fam Commun Health 2004;27(2):143-50 .

13. Castorina LL, Negri LM. The inclusion of siblings to promote generalisation in social skills training groups for boys with Asperger's syndrome. Aust J Psychol 2004;56(Suppl. S):167-8.

14. Celiberti DA. Training parents of children with autism to promote sibling play: randomized trials of three alternative training interventions. Dissert Abst Int A: Humanities and Social Sciences 1994;54(11-A):4033.

15. Chandler S, Christie P, Newson E, et al. Developing a diagnostic and intervention package for 2- to 3-year-olds with autism: outcomes of the frameworks for communication approach . Autism 2002;6(1):47-69.

16. Cheney CO. The comparative effectiveness of four instructional techniques with autistic adolescents. Dissert Abst Int 1985;45(9A):2833.

17. Chretien M, Kennedy SL, Moxness K. Implementation monitoring of an early intervention program for autistic children centred on the family. J Intell Disabil Res 2004;48:376.

18. Citterio DN. Autism and horses: intervention strategy from the point of view of a science of movement. Proceedings of the 9th International Therapeutic Riding Congress; Denver; CO. Denver: North American Riding for the Handicapped Association; 1997. p. 211.

19. Clark TR. The application of savant and splinter skills in the autistic population through curriculum design: a longitudinal multiple-replication case study [dissertation]. Sydney (Australia): University of New South Wales; 2001.

20. Cullen-Powell LA, Barlow JH, Cushway D. Exploring a massage intervention for parents and their children with autism: the implications for bonding and attachment. J Child Health Care 2005;9(4):245-55.

21. Dellatan AK. The use of music with chronic food refusal: a case study. Music Ther Perspect 2003;21(2):105-9.

22. Dimartino JA. Comparing two treatment methods in improving the handwriting skills of autistic students [dissertation]. Buffalo (NY): D'youville College; 1991.

23. Duran E. A university program provides services to young adults of severe handicaps and autism who are of limited english proficiency. El Paso: Texas University, 1984.

24. Dyer KI. The competition of stereotyped behavior with external reinforcers in autistic children [dissertation]. Santa Barbara (CA): University of California; 1985.

25. Edelson SM, Rimland B, Berger CL, et al. Evaluation of a mechanical hand-support for facilitated communication. J Autism Dev Disord 1998;28(2):153-7.

26. Eversole AJR. Social skills training with high functioning autistic adolescents [dissertation]. Denton (TX): University of North Texas; 1988.

27. Fiorile CA. An experimental analysis of the transformation of stimulus function from speaker to listener to speaker repertoires. Dissert Abst Int A: Humanities and Social Sciences 2005;(66):139A.

28. Fitzgerald GE, Werner JG. The use of the computer to support cognitive-behavioral interventions for students with behavioral disorders. J Comp Child Educ 1996;7(3-4):127-48.

29. Freeman BJ, Somerset T, Ritvo ER. Effect of duration of time out in suppressing disruptive behavior of a severely autistic child. Psychol Rep 1976;38(1):124-6.

30. Fullerton A, Coyne P. Developing skills and concepts for self-determination in young adults with autism. Focus Autism Other Dev Disabil 1999;14(1):42-52, 63.

31. Gal E, Goren-Bar D, Bauminger N, et al. Pilot study of enforced collaboration during computerized storytelling to enhance social communication of children with high-functioning autism. Cyberpsychol Behav 2006;9(6):674-5.

32. Garrels D. Autism, the ultimate learning disability: a case management approach. J Child Care 1983;1(4):23-35.

33. Gena A. Training and generalization of affective behavior displayed by youth with autism. Dissert Abst Int B: Sciences and Engineering 1995;55(11-B):5103.

34. Granpeesheh D. The effects of teaching common preschool games to autistic children on increasing peer interaction [dissertation]. Los Angeles (CA): University of California; 1990.

35. Green J. Social communication intervention effective for core impairments of autism. 2005;21(1):4.

36. Grindle CF. Human learning when reinforcement is delayed: the effects of response marking [dissertation]. Southampton (UK): University of Southampton; 2005.

37. Groden J. Procedures to increase social interaction among autistic adolescents: a multiple baseline analysis [dissertation]. Boston (MA): Boston College; 1982.

38. Gunter PL. An empirical and social validation of response covariation in the reduction of aberrant behavior in an autistic boy. Dissert Abst Int 1986;46(8-A):2264-5.

39. Gutstein S. The effectiveness of relationship development intervention in remediating core deficits of autism-spectrum children. J Dev Behav Pediatr 2004; 25(5):375.

40. Hastings R, Remington R, Kovshoff H, et al. Outcomes for families of children with autism in early intensive bhavioural intervention. J Intell Disabil Res 2004;48:327.

41. Heckaman KA. Effects of two response prompting procedures on disruptive behavior by students with moderate to severe disabilities during instruction on difficult tasks. Dissert Abst Int A: Humanities and Social Sciences 1996;56(9-A):3541.

42. Hedbring C. A comparison of two instructional interventions for remediating attentional deficits of autistic school-age children overselective in the visual modality. Dissert Abst Int 1983;44(6-A):1757.

43. Hinerman PS, Jenson WR, Walker GR, et al. Positive practice overcorrection combined with additional procedures to teach signed words to an autistic child. J Autism Dev Disord 1982;12(3):253-63.

44. Hobbs AM. Facilitating the inclusion of pupils with Asperger's syndrome in mainstream schools [dissertation]. Southampton (UK): University of Southampton; 2003.

45. Hollander AC. The effects of two holding positions upon self-injurious behavior, play, communication, and social behaviors in autistic children. Dissert Abst Int B: Sciences and Engineering 1994;54(7-B):3560.

46. Horn JK. A program of living skills training for autistic adolescents and adults in a residential treatment setting [dissertation]. San Diego (CA): United States International University; 1982.

47. Howlin P. A brief report on the elimination of long term sleeping problems in a 6-yr-old autistic boy. Behav Psychother 1984;12(3):257-60.

48. Ikeda MJ, Tucker R, Rankin B. Development, testing, and dissemination of nonaversive techniques for working with children with autism: demonstration of a "best practices" model for parents and teachers. The a-b-c-d model for supporting students with autism (antecedents, behaviors, consequences, data). Final report. Johnston, IA: Heartland Area Education Agency 11, 2002.

49. Jensen GD, Womack MG. Operant conditioning techniques applied in the treatment of an autistic child. Am J Orthopsychiat 1967;37(1):30-4.

50. Johnson J. The use of self-management procedures for maintaining responsiveness of autistic individuals in natural environments. Dissert Abst Int 1992;53(2-A):463.

51. Jung KE, Lee HJ, Lee YS, et al. Efficacy of sensory integration treatment based on virtual reality-tangible interaction for children with autistic spectrum disorder. Cyberpsychol Behav 2006;9(6):685.

52. Kennedy SL, Chretien M, Moxness K. Effects of an early intervention program for autistic children on parental stress and sense of competence. J Intell Disabil Res 2004;48:376.

53. Koegel LK. Teaching children with autism to use a self-initiated strategy to learn expressive vocabulary [dissertation]. Santa Barbara (CA): University of California; 1993.

54. Kozleski EB. The effects of nonverbal visual communication systems on autistic children [dissertation]. Greeley (CO): University of Northern Colorado; 1985.

55. Kuloglu-Aksaz N. The effect of informational counseling on the stress level of parents of children with autism in Turkey. J Autism Dev Disord 1994;24(1):109-10.

56. LeBlanc LA, Coates AM, Daneshvar S, et al. Using video modeling and reinforcement to teach perspective-taking skills to children with autism. J Appl Behav Anal 2003;36(2):253-7.

57. Lovaas OI, Schreibman L, Koegel RL. A behavior modification approach to the treatment of autistic children. J Autism Child Schiz 1974;4(2):111-29.

58. Luscre DM, Center DB. Procedures for reducing dental fear in children with autism. J Autism Dev Disord 1996;26(5):547-56.

59. Ma YC, Nagler J, Lee MH, et al. Impact of music therapy on the communication skills of toddlers with pervasive developmental disorder. Ann NY Acad Sci 2001;930:445-7.

60. MacDuff GS. Teaching children with autism to use photographic activity schedules: maintenance and generalization of complex response chains. Dissert Abst Int B: Sciences and Engineering 1995;55(9-B):4109.

61. Marshall NR, Hegrenes J. The use of written language as a communication system for an autistic child. J Speech Hear Disord 1972;37(2):258-61.

62. Matson JL. Simple correction for treating an autistic boy's encopresis. Psychol Rep 1977;41(3 Pt 1):802.

63. Mccarthy PAT. The effects of a differential reinforcement of low rates of responding intervention on behaviors of adults with autism during vocational training at a competitive job site (severely handicapped, mental retardation, employment, on-the-job training, behavior management) [dissertation]. Columbia (SC): University of South Carolina; 1984.

64. Mceachin JJ. Outcome of autistic children receiving intensive behavioral treatment: psychological status 3 to 12 years later [dissertation]. Los Angeles (CA): University of California, Los Angeles; 1987.

65. Mesibov GB. Social skills training with verbal autistic adolescents and adults: a program model. J Autism Dev Disord 1984;14(4):395-404.

66. Meyers ML. Teaching speech to autistic children through a program of specific music therapy [dissertation]. Union (NJ): Kean University; 1973.

67. Miller SB, Toca JM. Adapted melodic intonation therapy: a case study of an experimental language program for an autistic child. J Clin Psychiat 1979;40(4):201-3.

68. Moran DR. Developing generalized teaching skills in parents of autistic children. Dissert Abst Int 1989;49(7-A):1741.

69. Morrison RS. The effects of correspondence training and activity schedules on the play behavior of preschoolers with autism in an inclusive classroom [dissertation]. Columbus (OH): Ohio State University; 1999.

70. Mundschenk NA. The effects of sufficient exemplars on the social inclusion of autistic students. Dissert Abst Int 1993;53(7-A):2329.

71. Newey I, Collins S, Fowler D. Evaluating psychological interventions for young men with Asperger syndrome: cognitive behavioural therapy to address anxiety and teaching theory of mind to address social functioning. J Intell Disabil Res 2004;48:301.

72. Newman DL. The role of functional reinforcement in the acquisition and generalization of communicative language in an autistic population [dissertation]. New Brunswick (NY): Rutgers the State University of New Jersey; 1987.

73. Nichols JH. Social skills training with verbal autistic adolescents: a case study approach [dissertation]. Denton (TX): University of North Texas; 1988 .

74. Oke NJ. A group training program for siblings of children with autism: acquisition of language training procedures and related behavior change. Dissert Abst Int B: Sciences and Engineering 1994;54(9-B):4951.

75. Perry A, Bryson S, Bebko J. Multiple method validation study of facilitated communications: preliminary group results. J Devel Disabil 1993;2(2):1-19.

76. Pierson JA. The effectiveness of the effects of the Differential reinforcement of other behavior and gentle teaching methods on target behavior of children with autism. Dissert Abst Int 1990;50(10-A):3205.

77. Plienis AJ, Robbins FR, Dunlap G. Parent adjustment and family stress as factors in behavioral parent training for young autistic children. J Multihand Pers 1988;1(1):31-52.

78. Pries MG. The effects of rhythmic visual stimulation on the self-stimulatory behavior of severely developmentally disabled children [dissertation]. St. Louis (MO): Washington University; 1982.

79. Rinehart NJ, Brereton AV, Tonge BJ, et al. Autism: a parent-based early intervention. Aust J Psychol 2003;55(Suppl S):208.

80. Rolland C, Nanclares-Nogues V. Parent-therapist implemented, integrated treatment for young children with autism: a comparison with therapist-implemented IBT. J Dev Behav Pediatr 2005; 26(6):464.

81. Savidge C, Christie D, Brooks E, et al. A pilot social skills group for socially disorganized children. Clin Child Psychol Psychiatry 2004;9(2):289-96.

82. Schroeder C. Behavior modification training with autistic children comparing novel and familiar stimulus materials [dissertation]. Los Angeles (CA): University of California; 1981.

83. Scolnick B. Effects of electroencephalogram biofeedback with Asperger's syndrome. Int J Rehabil Res 2005;28(2):159-63.

84. Shadduck JA. A parent training program for parents of autistic children: the effects of intervention on family functioning. Dissert Abst Int 1990;50(7-B):3177.

85. Shumsky R. The effects of self-instructional training on the attending behavior, math performance and self-talk of children with autism [dissertation]. New Brunswick (NJ): Rutgers the State University of New Jersey; 1989.

86. Slaw KM. The amelioration of self-stimulation in an autistic teenager: a view toward the environment [dissertation]. Urbana-Champaign (IL): University of Illinois at Urbana-Champaign; 1987.

87. Smith MD, Belcher RG. Brief report: facilitated communication with adults with autism. J Autism Dev Disord 1993;23(1):175-83.

88. Solomon R, Necheles J, Ferch C, et al. Program evaluation of a statewide autism training and early intervention project: the University of Michigan Play project. Pediatr Res 2003;53(4 Part 2):79A-80.

89. Sorensen PL. The Hanen early language parent program: an evaluation of its effectiveness in modifying parent-child interactions when dealing with parents of school-aged, autistic children [dissertation]. Fredericton (NB): University of New Brunswick; 1990.

90. Stahmer AC. Teaching symbolic play to children with autism using pivotal response training: effects on play, language and interaction [dissertation]. San Diego (CA): University of California; 1993.

91. Strickland D. Evaluating a video-enhanced virtual reality program for teaching restaurant social skills to children with autism. Cyberpsychol Behav 2004;7(3):310.

92. Swinehart SR. Effects of auditory oral patterns as an intervention for expressive language with students with disabilities [dissertation]. Allendale (MI): Grand Valley State University; 1997.

93. Szabo IT. Promoting the generalization of appropriate play in autistic children. Dissert Abst Int 1987;47(7-B):3096.

94. Violette JD. Effects of interlocutor directiveness and lexical familiarity on an autistic child's immediate echolalia [dissertation]. Tucson (AR): University of Arizona; 1987.

95. Walker D. A comparison of one-to-one and group teaching instructional methods across classrooms serving students with autism and other developmental disabilities. Dissert Abst Int 1990;50(7-A):2019.

96. Ward AJ. Early childhood autism and structural therapy: outcome after 3 years. J Consult Clin Psychol 1978;46(3):586-7.

97. Warnick AS. An investigation of dance-movement therapy as a therapeutic modality for autistic children: a case study approach. Dissert Abst Int B: Sciences and Engineering 1996;57(1-B):0716.

98. Watling RL. The effect of sensory integration on behavior and engagement in young children with autistic spectrum disorders. Dissert Abst Int B: Sciences and Engineering 2004;65(5-B):2383.

99. White JH, Hornsby LG, Gordon R. Treating infantile autism with parent therapists. Int J Child Psychother 1972;1:83-95.

100. Wolosky PS. Teaching fundamental motor skills to children with autism/pervasive developmental disorder [dissertation]. Morgantown (WV): West Virginia University; 1992.

101. Zappella M. Young autistic children treated with ethologically oriented family therapy. Fam Syst Med 1990;8(1):14-27.

.**Excluded – Population; Non-ASD (N = 46)**

The following studies were excluded because they did not examine an ASD population.

1. Azrin NH, Besalel VA, Jamner JP, et al. Comparative study of behavioral methods of treating severe self-injury. Behav Resid Treat 1988;3(2):119-52.

2. Benaroya S, Wesley S, Ogilvie H, et al. Sign language and multisensory input training of children with communication and related developmental disorders. J Autism Child Schiz 1977;7(1):23-31.

3. Blake P, Moss T. The development of socialization skills in an electively mute child. Behav Res Ther 1967;5(4):349-56.

4. Britton LN, Carr JE, Landaburu HJ, et al. The efficacy of non-contingent reinforcement as treatment for automatically reinforced stereotypy . Behav Intervent 2002;17(2):93-103.

5. Contreras CM. Impact of parent education on parental expectations of children with autism. San Jose (CA): San Jose State University; 2006.

6. Craig-Unkefer LA. Increasing the social-communicative skills of at-risk preschool age children in a play context (language, intervention, head start). Dissert Abst Int A: Humanities and Social Sciences 1999;60(5-A):1445.

7. Dale PS, Crain-Thoreson C, Notari-Syverson A, et al. Parent-child book reading as an intervention technique for young children with language delays. Top Early Child Spec 1996;16(2):213-35.

8. Erguner-Tekinalp B, Akkok F. The effects of a coping skills training program on the coping skills, hopelessness, and stress levels of mothers of children with autism. Int J Adv Couns 2004;26(3):257-69.

9. Giarelli E, Souders M, Pinto-Martin J, et al. Intervention pilot for parents of children with autistic spectrum disorder. Pediatr Nurs 2005;31(5):389-99.

10. Harrison DR. Immediacy of feedback: its effects on academic performance. Sch Appl Learning Theor 1973;5(2):4-14.

11. Hudson R. The effect of brief training on attitudes and interactions of co-workers of individuals with autism. Dissert Abst Int B: Sciences and Engineering 2005;66(2-B):1207.

12. Ishiguro C, Ohashi K, Mizukoshi K. Dysequilibrium in children with autism or minimal brain-dysfunction and its improvement with sensory integration therapy. Brain Dev-Jpn 1985;7(2):165.

13. Jepsen RH, VonThaden K. The effect of cognitive education on the performance of students with neurological developmental disabilities. Neurorehab 2002;17(3):201-9.

14. Kaiser AP, Ostrosky MM, Alpert CL. Training teachers to use environmental arrangement and milieu teaching with nonvocal preschool children. J Assoc Pers Severe Hand 1993;18(3):188-99.

15. Koegel RL, Rincover A. Some detrimental effects of using extra stimuli to guide learning in normal and autistic children. J Abnorm Child Psych 1976;4(1):59-71.

16. Leblanc MP, Ricciardi JN, Luiselli JK. Improving discrete trial instruction by paraprofessional staff through an abbreviated performance feedback intervention. Educ Treat Child 2005;28(1):76-82.

17. Liddle K. Implementing the picture exchange communication system (PECS). Int J Lang Comm Dis 2001;36:391-5.

18. Longenecker HQ. Parent stress related to having a child with autism: Impact of educational programming. Dissert Abst Int B: Sciences and Engineering 2003;64(1-B):424.

19. Mayo YL. Long term follow-up in parent training: a low cost alternative for parents in developing countries. Dissert Abst Int A: Humanities and Social Sciences 1997;57(10-A):4556.

20. Mazuryk GF, Barker P, Harasym L. Behavior therapy for autistic children: a study of acceptability and outcome. Child Psychiat Hum D 1978;9(2):119-25.

21. McLean LP, McLean JE. A language training program for nonverbal autistic children. J Speech Hear Disord 1974;39(2):186-93.

22. Milch R. Scheduling variables in parent training: a comparison of ten and twenty week behavioral parent training programs for parents of autistic children. Dissert Abst Int 1983;44(5-B):1600.

23. Parsons S, Mitchell P, Leonard A. The use and understanding of virtual environments by adolescents with autistic spectrum disorders. J Autism Dev Disord 2004;34(4):449-66.

24. Paul R, Cohen DJ. Responses to contingent queries in adults with mental retardation and pervasive developmental disorders. Appl Psycholinguist 1984;5(4):349-57.

25. Polirstok SR, Dana L, Buono S, et al. Improving functional communication skills in adolescents and young adults with severe autism using gentle teaching and positive approaches. Top Lang Disord 2003;23(2):146-53.

26. Randell T, Hall M, Bizo L, et al. DTkid: interactive simulation software for training tutors of children with autism. J Autism Dev Dis 2007;37 ( 4):637-47.

27. Rix K. Teaching a mother to attend differentially to her mentally handicapped child's behaviour. Behav Psychother 1988;16(2):122-32.

28. Romanczyk RG, Diament C, Goren ER, et al. Increasing isolate and social play in severely disturbed children: intervention and postintervention effectiveness. J Autism Child Schiz 1975;5(1):57-70.

29. Russo DC, Koegel RL, Lovaas OI. A comparison of human and automated instruction of autistic children. J Abnorm Child Psych 1978;6(2):189-201.

30. Schell RE, Adams WP. Training parents of a young child with profound behavior deficits to be teacher-therapists. J Spec Educ 1968;2(4):439-54.

31. Schwartz IS, Garfinkle AN, Bauer J. The picture exchange communication system: communicative outcomes for young children with disabilities. Top Early Child Spec 1998;18(3):144-59.

32. Shapiro T, Frosch E, Arnold S. Communicative interaction between mothers and their autistic children: application of a new instrument and changes after treatment. J Am Acad Child Adolesc Psychiatry 1987;26(4):485-90.

33. Shu BC, Lung FW. The effect of support group on the mental health and quality of life for mothers with autistic children. J Intell Disabil Res 2005;49(1):47-53.

34. Sigafoos J, Drasgow E. Conditional use of aided and unaided AAC: a review and clinical case demonstration. Focus Autism Other Dev Disabil 2001;16(3):152-61 .

35. Smith C, Goddard S, Fluck M. A scheme to promote social attention and functional language in young children with communication difficulties and autistic spectrum disorder. Educ Psychol Pract 2004;20(4):319-33.

36. Smith SA, Press B, Koenig KP, et al. Effects of sensory integration intervention on self-stimulating and self-injurious behaviors. Am J Occup Ther 2005;59(4):418-25.

37. Smith T, Groen AD, Wynn JW. Randomized trial of intensive early intervention for children with pervasive developmental disorder [errata]. Am J Ment Retard 2001;106(3):208.

38. Spinks T. A language proposal to maximize communication for an echolalic responding adult. Austral J Spec Educ 1987;11(1):28-35.

39. Stange EJ Jr. Visually based social skills training for students with autism spectrum disorder [dissertation]. Tampa (FL): University of South Florida; 2001.

40. Stermac L, Josefowitz N. A board game for teaching social skills to institutionalized adolescents. J Child Care 1985;2(3):31-8.

41. Szempruch J, Jacobson JW. Evaluating facilitated communications of people with developmental disabilities. Res Dev Disabil 1993;14(4):253-64.

42. Taubman M, Brierley S, Wishner J, et al. The effectiveness of a group discrete trial instructional approach for preschoolers with developmental disabilities. Res Dev Disabil 2001;22(3):205-19.

43. Thaut MH. Music therapy as a treatment tool for autistic children [dissertation]. East Lansing (MI): Michigan State University; 1980.

44. Thiemann KS, Goldstein H. Social stories, written text cues, and video feedback: effects on social communication of children with autism. J Appl Behav Anal 2001;34(4):425-46.

45. Williams LL. The influence of human contact on the output of facilitated communication [dissertation]. Athens (OH): Ohio University; 1993.

46. Wimpory DC, Nash S. Musical interaction therapy: therapeutic play for children with autism. Child Lang Teach Ther 1999;15(1):17-28.

**Excluded – Study design was not a Clinical Trial or an Observational Analytical Cohort Study (N = 785)**

The following studies were excluded because the study design was not a clinical trial or an observational analytical cohort study (as defined by the review)

1. Accardo CM. Generalization of imitation skills among children with autism during the first 3 months of early intensive behavior treatment programs. Dissert Abst Int A: Humanities and Social Sciences 2004;65(6-A):2085.

2. Adams L, Gouvousis A, VanLue M, et al. Social story intervention: improving communication skills in a child with an autism spectrum disorder. Focus Autism Other Dev Disabil 2004;19(2):87-94.

3. Agosta E, Graetz JE, Mastropieri MA, et al. Teacher-researcher partnerships to improve social behavior through social stories. Interv Sch Clin 2004;39(5):276-87 .

4. Ahearn WH. Using simultaneous presentation to increase vegetable consumption in a mildly selective child with autism. J Appl Behav Anal 2003;36(3):361-5.

5. Aiken JM. The effects of long and short intertrial intervals on three autistic children's stereotypic behavior [dissertation]. Nashville (TN): Peabody College for Teachers of Vanderbilt University; 1982.

6. Aiken JM, Salzberg CL. The effects of a sensory extinction procedure on stereotypic sounds of two autistic children. J Autism Dev Disord 1984;14(3):291-9.

7. Akmanoglu N, Batu S. Teaching pointing to numerals to individuals with autism using simultaneous prompting. Educ Training Dev Disabil 2004;39(4):326-36.

8. Akmanoglu-Uludag N, Batu S. Teaching naming relatives to individuals with autism using simultaneous prompting. Educ Training Dev Disabil 2005;40(4):401-10 .

9. Albert BF. The effect of environmental "softness" upon the behaviors of autistic children. Dissert Abst Int B: Sciences and Engineering 2003;64(3-B):1546.

10. Alcantara PR. Effects of videotape instructional package on purchasing skills of children with autism. Except Children 1994;61(1):40-55.

11. Alonim H. The Mifne Method-ISRAEL: early intervention in the treatment of autism/PDD: a therapeutic programme for the nuclear family and their child. J Child Adolesc Ment Health 2004;16(1):39-43.

12. Amuso SA. The effect animal assisted therapy has on the spontaneous interaction of autistic and/or pervasively developmentally delayed children [dissertation]. New York (NY): Touro College; 2003.

13. Anderson AE. Augmentative communication and autism: a comparison of sign language and the picture exchange communication system. Dissert Abst Int 2002;62(9B):4269.

14. Anderson SR, Avery DL, DiPietro EK, et al. Intensive home-based early intervention with autistic children. Educ Treat Child 1987;10(4):352-66.

15. Ando H. Training autistic children to urinate in the toilet through operant conditioning techniques. J Autism Child Schiz 1977;7(2):151-63.

16. Angell SL. Using conjoint behavioral consultation with children who have autism: effectiveness, acceptability, and generalization of skills. Dissert Abst Int A: Humanities and Social Sciences 2006;66(7-A):2490.

17. Apple AL. A comparison of the use of constant time delay alone and constant time delay with instructive feedback to teach children with autism to discriminate stimuli by function, feature and class [dissertation]. Seattle (WA): University of Washington; 2005.

18. Apple AL, Billingsley F, Schwartz IS. Effects of video modeling alone and with self-management on compliment-giving behaviors of children with high-functioning ASD. J Pos Behav Interv 2005;7(1):33-46.

19. Arco L. Improving program outcome with process-based feedback. J Org Behav Managem 1997;17(1):37-64.

20. Arntzen E, Gilde K, Pedersen E. Generalized schedule following in a youth with autism. Scand J Behav Ther 1998;27(3):135-41.

21. Ault MJ, Wolery M, Gast DL, et al. Comparison of response prompting procedures in teaching numeral identification to autistic subjects. J Autism Dev Disord 1988;18(4):627-36.

22. Ayres AJ, Mailloux ZK. Possible pubertal effect on therapeutic gains in an autistic girl. Am J Occup Ther 1983;37(8):535-40.

23. Bailey SL, Pokrzywinski J, Bryant LE. Using water mist to reduce self-injurious and stereotypic behavior. Appl Res Ment Retard 1983;4(3):229-41.

24. Bainbridge N, Myles BS. The use of priming to introduce toilet training to a child with autism. Focus Autism Other Dev Disabil 1999;14(2):106-9.

25. Baker LJ, Milner Y. Sensory reinforcement with autistic children. Behav Psychother 1985;13(4):328-41.

26. Baker MJ. Incorporating children with autism's thematic ritualistic behaviors into games to increase social play interactions with siblings. Dissert Abst Int B: Sciences and Engineering 2000;60(12-B):6353.

27. Baker MJ. Incorporating the thematic ritualistic behaviors of children with autism into games: increasing social play interactions with siblings. J Pos Behav Interv 2000;2(2):66-84.

28. Barnes MA. The effects of "westpaw" on bilateral coordination in children with perceptual-motor deficits: a case study [dissertation]. New York (NY): Touro College; 1994.

29. Barnhill GP, Cook KT, Tebbenkamp K, et al. The effectiveness of social skills intervention targeting nonverbal communication for adolescents with asperger syndrome and related pervasive developmental delays. Focus Autism Other Dev Disabil 2002;17(2):112-8.

30. Barrera RD, Lobato-Barrera D, Sulzer-Azaroff B. A simultaneous treatment comparison of three expressive language training programs with a mute autistic child. J Autism Dev Disord 1980;10(1):21-37.

31. Barrera RD, Sulzer-Azaroff B. An alternating treatment comparison of oral and total communications training programs with echolalic autistic children. J Appl Behav Anal 1983;16(4):379-94.

32. Barry LM, Burlew SB. Using social stories to teach choice and play skills to children with autism. Focus Autism Other Dev Disabil 2004;19(1):45-51.

33. Barry TD, Klinger LG, Lee JM, et al. Examining the effectiveness of an outpatient clinic-based social skills group for high-functioning children with autism. J Autism Dev Disord 2003;33(6):685-701.

34. Bartels WM. Implementation of the Developmental, Individual-Differences, Relationship-Based (DIR) model in a preschool for children with autism spectrum disorders. Dissert Abst Int B: Sciences and Engineering 2004;65(5-B):2612.

35. Bartlett D, Ora JP, Brown E, et al. The effects of reinforcement on psychotic speech in a case of early infantile autism, age 12. J Behav Ther Exp Psy 1971;2(2):145-9.

36. Bartman S, Freeman N. Teaching language to a two-year-old with autism. J Devel Disabil 2003;10(1):47-53.

37. Bauminger N. The facilitation of social-emotional understanding and social interaction in high-functioning children with autism: intervention outcomes. J Autism Dev Disord 2002;32(4):283-98.

38. Beadle-Brown J, Murphy G, Wing L. The Camberwell cohort 25 years on: characteristics and changes in skills over time. J Appl Res Intellect 2006;19(4):317-29.

39. Bebko JM, Perry A, Bryson S. Multiple method validation study of facilitated communication: II individual differences and subgroup results. J Autism Dev Disord 1996;26(1):19-42.

40. Bedrosian J, Lasker J, Speidel K, et al. Enhancing the written narrative skills of an AAC student with autism: evidence-based research issues. Top Lang Disord 2003;23(4):305-24.

41. Beglinger L, Smith T. Concurrent validity of social subtype and IQ after early intensive behavioral intervention in children with autism: a preliminary investigation. J Autism Dev Disord 2005;35(3):295-303.

42. Beisler JM, Tsai LY. A pragmatic approach to increase expressive language skills in young autistic children. J Autism Dev Disord 1983;13(3):287-303.

43. Belcher TL. Behavioral treatment vs. behavioral control: a case study. J Dev Phys Disabil 1995;7(3):235-41.

44. Belchic JK, Harris SL. The use of multiple peer exemplars to enhance the generalization of lay skills to the siblings of children with autism. Child Fam Behav Ther 1994;16(2):1-25.

45. Bellini S, Akullian J, Hopf A. Increasing social engagement in young children with autism spectrum disorders using video self-modeling. School Psychol Rev 2007;36 (1):80-90.

46. Bellon ML, Ogletree BT, Harn WE. Repeated storybook reading as a language intervention for children with autism: a case study on the application of scaffolding. Focus Autism Other Dev Disabil 2000;15(1):52-8.

47. Ben-Arieh J. A comparative study of joint action routines (JAR) and discrete trial training (DTT) [dissertation]. Lawrence (KS): University of Kansas; 2003.

48. Ben-Itzchak E, Zachor DA. The effects of intellectual functioning and autism severity on outcome of early behavioral intervention for children with autism. Res Dev Disabil 2007;28 ( 3):287-303.

49. Ben-Tall A. Treatment of social-communicative behaviors associated with restricted repetitive interests in high-functioning adolescents with autism. Dissert Abst Int B: Sciences and Engineering 1999;59(10-B):5568.

50. Bentivegna S, Schwartz L, Deschner D. The use of art with an autistic child in residential care. Am J Art Ther 1983;22(2):51-6.

51. Berkowitz S. A comparison of two methods of prompting in training discrimination of communication book pictures by autistic students. J Autism Dev Disord 1990;20(2):255-62.

52. Berman CM. The effects of rate contingent consequences and charting on response rates for two children with autism [dissertation]. Denton (TX): University of North Texas; 2004.

53. Bernard-Opitz V, Sriram N, Sapuan S. Enhancing vocal imitations in children with autism using the IBM SpechViewer. Autism 1999;3(2):131-47.

54. Bigelow KM, Huynen KB, Lutzker JR. Using a changing criterion design to teach fire escape to a child with developmental disabilities. J Dev Phys Disabil 1993;5(2):121-8.

55. Biklen D, Morton MW, Saha SN, et al. "I amn not a utistivc on thje typ" ("I'm not autistic on the typewriter"). Disab Hand Soc 1991;6(3):161-80.

56. Bingham-Watts KL. Effects of a remote-controlled tactile prompt on the initiation skills of a child with autism [dissertation]. Denton (TX): University of North Texas; 1999.

57. Blank M, Milewski J. Applying psycholinguistic concepts to the treatment of an autistic child. Appl Psycholinguist 1981;2(1):65-84.

58. Blasco SP. Case study: art expression as a guide to music therapy. Am J Art Ther 1978;17(2):51-6.

59. Bledsoe R, Myles BS, Simpson RL. Use of a social story intervention to improve mealtime skills of an adolescent with Asperger syndrome. Autism 2003;7(3):289-95.

60. Blew PA, Schwartz IS, Luce SC. Teaching functional community skills to autistic children using nonhandicapped peer tutors. J Appl Behav Anal 1985;18(4):337-42.

61. Blindert HD, Hartridge CL, Gwadry FG. Case study: controlling self-injurious escape behaviors. Behav Intervent 1995;10(3):173-9.

62. Bock MA. Sorting laundry: categorization strategy application to an authentic learning activity by children with autism. Focus Autism Other Dev Disabil 1999;14(4):220-30.

63. Boettcher MA. Teaching social conversation skills to children with autism through self-management: an analysis of treatment gains and meaningful outcomes. Dissert Abst Int B: Sciences and Engineering 2004;65(6-B):3135.

64. Bomba C, O'Donnell L, Markowitz C, et al. Evaluating the impact of facilitated communication on the communicative competence of fourteen students with autism. J Autism Dev Disord 1996;26(1):43-58.

65. Born-Miller KL. The use of an errorless teaching procedure to teach children with autism for whom trial-and-error teaching has failed. Dissert Abst Int B: Sciences and Engineering 2002;63(4-B):2088.

66. Bosseler A, Massaro DW. Development and evaluation of a computer-animated tutor for vocabulary and language learning in children with autism. J Autism Dev Disord 2003;33(6):653-72.

67. Boulware GL. A comparison of simultaneous and most-to-least prompting procedures in teaching receptive identification of pictures to toddlers with autism [dissertation]. Seattle (WA): University of Washington; 2001.

68. Brady DO. The effects of simultaneous treatments for language acquisition in an autistic child [dissertation]. Norman (OK): The University of Oklahoma; 1975.

69. Brady MP, McEvoy MA, Wehby J, et al. Using peers as trainers to increase an autistic child's social interactions. Except Children 1987;34(3):213-9.

70. Braithwaite KL, Richdale AL. Functional communication training to replace challenging behaviors across two behavioral outcomes. Behav Intervent 2000;15(1):21-36.

71. Brawley ER, Harris FR, Allen KE, et al. Behavior modification of an autistic child. Behav Sci 1969;14(2):87-97.

72. Breen C. The training and generalization of social interaction during breaktime at two job sites in the natural environment. English, 1984.

73. Breen C, Haring T, Pitts-Conway V, et al. The training and generalization of social interaction during breaktime at two job sites in the natural environment. J Assoc Pers Severe Hand 1985;10(1):41-50.

74. Brightwell A. Childhood autism and the effect of an integrated preschool program. Dissert Abst Int 2003;63(11A):3850.

75. Brim D. Analysis of social referencing skills in children with autism [dissertation]. Dissert Abst Int 2003;63(9B):4401.

76. Brookman-Frazee L. Using parent/clinician partnerships in parent education programs for children with autism. J Pos Behav Interv 2004;6(4):195-213.

77. Brown JL. Script fading as a procedure for teaching unscripted language to children with autism. Dissert Abst Int B: Sciences and Engineering 2003;(64):1477-B.

78. Brown MC. Antecedent selection: the use of simplified speech versus typical speech in discrete trial teaching formats [dissertation]. Boca Raton (FL): Florida Atlantic University; 2001.

79. Brown RA, Pace ZS. Treatment of extreme negativism and autistic behavior in a 6 year old boy. Except Children 1969;36(2):115-22.

80. Brown WH, Ragland EU, Fox JJ. Effects of group socialization procedures on the social interactions of preschool children. Res Dev Disabil 1988;9(4):359-76.

81. Brownell MD. Musically adapted social stories to modify behaviors in students with autism: four case studies. J Music Ther 2002;39(2): 117-44.

82. Browning AD. Choice making: determining its effects on challenging behaviors and positive affect for a student with autism [dissertation]. Fullerton (CA): California State University, Fullerton; 2001.

83. Browning RM. Treatment effects of a total behavior modification program with five autistic children. Behav Res Ther 1971;9(4):319-27.

84. Bruinsma YEM. Increases in the joint attention behavior of eye gaze alternation to share enjoyment as a collateral effect of pivotal response treatment for three children with autism [dissertation]. Santa Barbara (CA): University of California, Santa Barbara; 2004.

85. Bryan LC, Gast DL. Teaching on-task and on-schedule behaviors to high-functioning children with autism via picture activity schedules. J Autism Dev Disord 2000;30(6):553-67.

86. Buch GA. Teaching parents and paraprofessionals how to provide behavioral intensive early intervention for children with autism and pervasive developmental disorder. Dissert Abst Int B: Sciences and Engineering 1996;56(9-B):5153.

87. Buckley SD, Newchok DK. Differential impact of response effort within a response chain on use of mands in a student with autism. Res Dev Disabil 2005;26(1):77-85.

88. Buckley SD, Strunck PG, Newchok DK. A comparison of two multicomponent procedures to increase food consumption. Behav Intervent 2005;20(2):139-46.

89. Buffington DM. Procedures for teaching appropriate gestural communication skills to children with autism. Dissert Abst Int B: Sciences and Engineering 1997;57(9-B):5900.

90. Buffington DM, Krantz PJ, McClannahan LE, et al. Procedures for teaching appropriate gestural communication skills to children with autism. J Autism Dev Disord 1998;28(6):535-45.

91. Buggey T. Video self-modeling applications with students with autism spectrum disorder in a small private school setting. Focus Autism Other Dev Disabil 2005;20(1):52-63.

92. Buggey T, Toombs K, Gardener P, et al. Training responding behaviors in students with autism: using videotaped self-modeling. J Pos Behav Interv 1999;1(4):205-14.

93. Burt DB, Fuller SP, Lewis KR. Competitive employment of adults with autism. J Autism Dev Disord 1991;21(2):237-42.

94. Cafiero JM. The effect of an augmentative communication intervention on the communication, behavior, and academic program of an adolescent with autism. Focus Autism Other Dev Disabil 2001;16(3):179-89.

95. Cafiero JM. Teaching parents of children with autism Picture Communication Symbols as a natural language to decrease levels of family stress. Dissert Abst Int A: Humanities and Social Sciences 1996;56(7-A):2636.

96. Cale SI. Context-based assessment and intervention for problem behavior in children with autism spectrum disorders. Dissert Abst Int B: Sciences and Engineering 2005;66(6-B):3399.

97. Callahan K, Rademacher JA. Using self-management strategies to increase the on-task behavior of a student with autism. J Pos Behav Interv 1999;1(2):117-22.

98. Camburn MM. Intensive behavioral treatment of pervasive developmental disorder-nos: a case study. Dissert Abst Int B: Sciences and Engineering 1995;56(2-B):1096.

99. Campbell RV, Lutzker JR. Using functional equivalence training to reduce severe challenging behavior: a case study. J Dev Phys Disabil 1993;5(3):203-16.

100. Cardaciotto LA, Herbert JD. Cognitive behavior therapy for social anxiety disorder in the context of asperger's syndrome: a single-subject report. Cogn Behav Pract 2004;11(1):75-81.

101. Carr D. Effects of exemplar training in exclusion responding on auditory-visual discrimination tasks with children with autism. J Appl Behav Anal 2003;36(4):507-24.

102. Carr EG, Binkoff JA, Kologinsky E, et al. Acquisition of sign language by autistic children I: expressive labelling. J Appl Behav Anal 1978;11(4):489-501.

103. Carr EG, Dores PA. Patterns of language acquisition following simultaneous communication with autistic children. Anal Interv Dev Disabil 1981;1(3-4):347-61.

104. Carr EG, Kemp DC. Functional equivalence of autistic leading and communicative pointing: analysis and treatment. J Autism Dev Disord 1989;19(4):561-78.

105. Carter CM. Using choice with interactive play to increase language skills in children with autism [dissertation]. Santa Barbara (CA): University of California; 2000.

106. Case-Smith J, Bryan T. The effects of occupational therapy with sensory integration emphasis on preschool-age children with autism. Am J Occup Ther 1999;53(5):489-97.

107. Casey LO. Development of communicative behavior in autistic children: a parent program using manual signs. J Autism Child Schiz 1978;8(1):45-59.

108. Charlop-Christy MH, Carpenter M, Le L, et al. Using the picture exchange communication system (PECS) with children with autism: assessment of PECS acquisition, speech, social-communicative behavior, and problem behavior. J Appl Behav Anal 2002;35(3):213-31.

109. Charlop-Christy MH, Carpenter MH. Modified incidental teaching sessions: a procedure for parents to increase spontaneous speech in their children with autism. J Pos Behav Interv 2000;2(2):98-112.

110. Charlop-Christy MH, Daneshvar S. Using video modeling to teach perspective taking to children with autism. J Pos Behav Interv 2003;5(1):12-21.

111. Charlop-Christy MH, Haymes LK. Using objects of obsession as token reinforcers for children with autism. J Autism Dev Disord 1998;28(3):189-98.

112. Charlop-Christy MH, Haymes LK. Using obsessions as reinforcers with and without mild reductive procedures to decrease inappropriate behaviors of children with autism. J Autism Dev Disord 1996;26(5):527-46.

113. Charlop-Christy MH, Kelso SE. Teaching children with autism conversational speech using a cue card/written script program. Educ Treat Child 2003;26(2):108-27.

114. Charlop-Christy MH, Le L, Freeman KA. A comparison of video modeling with in vivo modeling for teaching children with autism. J Autism Dev Disord 2000;30(6):537-52.

115. Charlop MH, Kurtz PF, Casey FG. Using aberrant behaviors as reinforcers for autistic children. J Appl Behav Anal 1990;23(2):163-81.

116. Charlop MH, Kurtz PF, Milstein JP. Too much reinforcement, too little behavior: assessing task interspersal procedures in conjunction with different reinforcement schedules with autistic children. J Appl Behav Anal 1992;25(4):795-808.

117. Charlop MH, Milstein JP. Teaching autistic children conversational speech using video modeling. J Appl Behav Anal 1989;22(3):275-85.

118. Charlop MH, Schreibman L, Thibodeau MG. Increasing spontaneous verbal responding in autistic children using a time delay procedure. J Appl Behav Anal 1985;18(2):155-66.

119. Charlop MH, Schreibman L, Tryon AS. Learning through observation: the effects of peer modeling on acquisition and generalization in autistic children. J Abnorm Child Psych 1983;11(3):355-66.

120. Charlop MH, Trasowech JE. Increasing autistic children's daily spontaneous speech. J Appl Behav Anal 1991;24(4):747-61.

121. Charlop MH, Walsh ME. Increasing autistic children's spontaneous verbalizations of affection: an assessment of time delay and peer modeling procedures. J Appl Behav Anal 1986;19(3):307-14.

122. Chiang I. Effects of a therapeutic recreation intervention within a technology-based physical activity context on the social interaction of male youth with autism spectrum disorders [dissertation]. Bloomington (IN): Indiana University; 2003.

123. Chin HY, Bernard-Opitz V. Teaching conversation skills to children with autism: effect on the development of a theory of mind. J Autism Dev Disord 2000;30(6):569-83.

124. Cicchino C, Monteleone V. The efficacy of sensory integration therapy on pre-school children with autism and its effects on verbalizations and engagement [dissertation]. New York (NY): Touro College; 2000.

125. Cicero FR, Pfadt A. Investigation of a reinforcement-based toilet training procedure for children with autism. Res Dev Disabil 2002;23(5):319-31.

126. Clark CP. The differential effects of simplified speech versus typical speech on response accuracy in discrete trial teaching formats with prekindergarten-age students with autism. Dissert Abst Int A: Humanities and Social Sciences 2000;(61):133A-4.

127. Clark K, Green G. Comparison of two procedures for teaching dictated-word/symbol relations to learners with autism. J Appl Behav Anal 2004;37(4):503-7.

128. Clarke JC, Thomason S. The use of an aversive smell to eliminate autistic self-stimulatory behavior. Child Fam Behav Ther 1983;5(3):51-67.

129. Clarke S, Dunlap G, Vaughn B. Family-centered, assessment-based intervention to improve behavior during an early morning routine. J Pos Behav Interv 1999;1(4):235-41.

130. Clauss EL. Effects of music on attention and self-stimulatory behaviors in autistic people [dissertation]. Hempstead (NY): Hofstra University; 1994.

131. Cochran KM. The use of prompting with students with autism in the general education setting [dissertation]. Fullerton (CA): California State University; 2005.

132. Coleman CL, Holmes PA. The use of noncontingent escape to reduce disruptive behaviors in children with speech delays. J Appl Behav Anal 1998;31(4):687-90 .

133. Coleman SL, Stedman JM. Use of a peer model in language training in an echolalic child. J Behav Ther Exp Psy 1974;5(3-4):275-9.

134. Collier D. A comparison of two models designed to teach autistic children a motor task [dissertation]. Montreal (QC): Mcgill University (Canada); 1985 .

135. Colligan RC, Bellamy CM. Effects of a two year treatment program for a young autistic child. Psychother Theor Res 1968;5(4):214-9.

136. Conroy MA, Asmus JM, Sellers JA, et al. The use of an antecedent-based intervention to decrease stereotypic behavior in a general education classroom: a case study. Focus Autism Other Dev Disabil 2005;20(4):223-30.

137. Cook DG. A sensory approach to the treatment and management of children with autism. Focus Autistic Behav 1990;5(6):1-19.

138. Cook KET. An analysis of two approaches to social skills instruction for students with Asperger syndrome [dissertation]. Lawrence (KS): University of Kansas; 2002.

139. Cordisco LK, Strain PS, Depew N. Assessment for generalization of parenting skills in home settings. J Assoc Per Sev Handic 1988;13(3):202-10.

140. Cotter MKW. Improving the social behavior of high-functioning children with autism: a social skills support group intervention [dissertation]. Birmingham (AB): University of Alabama; 1997.

141. Coyle C, Cole P. A videotaped self-modelling and self-monitoring treatment program to decrease off-task behaviour in children with autism. J Intell Dev Dis 2004;29(1):3-15.

142. Crager DE, Horvath LS. The application of social skills training in the treatment of a child with asperger's disorder. Clin Case Stud 2003;2(1):34-49.

143. Craighead WE, O'Leary KD, Allen JS. Teaching and generalization of instruction-following in an "autistic" child. J Behav Ther Exp Psy 1973;4(2):171-6.

144. Crozier S, Tincani MJ. Using a modified social story to decrease disruptive behavior of a child with autism. Focus Autism Other Dev Disabil 2005;20(3):150-7.

145. Cullain RE. The effects of social stories on anxiety levels and excessive behavioral expressions of elementary school-aged children with autism. Dissert Abst Int A: Humanities and Social Sciences 2002;62(7-A):2383.

146. Cullen LA, Barlow JH, Cushway D. Positive touch, the implications for parents and their children with autism: an exploratory study. Complement Ther Clin Pract 2005;11(3):182-9.

147. Cutforth ER. Reducing the inappropriate behavior of a child with autistic disorder [dissertation]. San Jose (CA): San Jose State University; 2000.

148. D'Ateno P, Mangiapanello K, Taylor BA. Using video modeling to teach complex play sequences to a preschooler with autism. J Pos Behav Interv 2003;5(1):5-11.

149. Dadds M, Schwartz S, Adams T, et al. The effects of social context and verbal skill on the stereotypic and task-involved behavior of autistic children. J Child Psychol Psyc 1988;29(5):669-76.

150. Dauphin M, Kinney EM, Stromer R. Using video-enhanced activity schedules and matrix training to teach sociodramatic play to a child with autism. J Pos Behav Interv 2004;6(4):238-50.

151. Dawson G, Galpert L. Mothers' use of imitative play for facilitating social responsiveness and toy play in young autistic children. Dev Psychopathol 1990;2(2):151-62.

152. Delano M, Snell ME. The effects of social stories on the social engagement of children with autism. J Pos Behav Interv 2006;8(1):29-42.

153. DeLeon IG, Anders BM, Rodriguez-Catter V, et al. The effects of noncontingent access to single-versus multiple-stimulus sets on self-injurious behavior. J Appl Behav Anal 2000;33(4):623-26.

154. DeLeon IG, Neidert PL, Anders BM, et al. Choices between positive and negative reinforcement during treatment for escape-maintained behavior. J Appl Behav Anal 2001;34(4):521-5.

155. Demiri V. Teaching social skills to children with autism using social stories: an empirical study. Dissert Abst Int B: Sciences and Engineering 2004;65(5-B):2619.

156. Deris AR, Hagelman EM, Schilling K, et al. Using a weighted or pressure vest for a child with autistic spectrum disorder [web page]. 2006; Accessed May, 2007.

157. Dettmer S, Simpson RL, Myles BS, et al. The use of visual supports to facilitate transitions of students with autism. Focus Autism Other Dev Disabil 2000;15(3):163-9.

158. Dexter ME. The effects of aided language stimulation upon verbal output and augmentative communication during storybook reading for children with pervasive developmental disabilities [dissertation]. Baltimore (MD): Johns Hopkins University; 1998.

159. Din FS, McLaughlin D. Teach children with autism with the discrete-trial approach. Annual Conference of the Eastern Educational Research Association; Clearwater, FL.; 2000.

160. Dixon MR, Cummings A. Self-control in children with autism: response allocation during delays to reinforcement. J Appl Behav Anal 2001;34(4):491-5.

161. Dixon RS, Moore DW, Hartnett N, et al. Reducing inappropriate questioning behaviour in an adolescent with autism: a case study. Behav Change 1995;12(3):163-6.

162. Donnellan AM, LaVigna GW. Nonaversive control of socially stigmatizing behaviors. Pointer 1986;30(4):25-31.

163. Dooley P, Wilczenski FL, Torem C. Using an activity schedule to smooth school transitions. J Pos Behav Interv 2001;3(1):57-61.

164. Doyle MM. An interplay-based social skills group for children with Asperger's syndrome. Dissert Abst Int A: Humanities and Social Sciences 2001;62(4-A):1583.

165. Dozier CL, Carr JE, Enloe K, et al. Using fixed-time schedules to maintain behavior: a preliminary investigation. J Appl Behav Anal 2001;34(3):337-40.

166. Ducharme JM, Drain TL. Errorless academic compliance training: improving generalized cooperation with parental requests in children with autism. J Am Acad Child Adolesc Psychiatry 2004;43(2):163-71.

167. Ducharme JM, Lucas H, Pontes E. Errorless embedding in the reduction of severe maladaptive behavior during interactive and learning tasks. Behav Ther 1994;25(3):489-501.

168. Dudley LL, Johnson C, Barnes RS. Decreasing rumination using a starchy food satiation procedure. Behav Intervent 2002;17(1):21-9.

169. Duker PC, Rasing E. Effects of redesigning the physical environment on self-stimulation and on-task behavior in three autistic-type developmentally disabled individuals. J Autism Dev Disord 1989;19(3):449-60.

170. Duker PC, Schaapveld M. Increasing on-task behaviour through interruption-prompting. J Intell Disabil Res 1996;40 (4):291-7.

171. Dunlap G, Fox L. A demonstration of behavioral support for young children with autism. J Pos Behav Interv 1999;1(2):77-87.

172. Dunlap G, Koegel RL, Johnson J, et al. Maintaining performance of autistic clients in community settings with delayed contingencies. J Appl Behav Anal 1987;20(2):185-91.

173. Duran E. Teaching janitorial skills to autistic adolescents. Adolescence 1985;20(77):225-32.

174. Dyches TT. Effects of switch training on the communication of children with autism and severe disabilities. Focus Autism Other Dev Disabil 1998;13(3):151-62.

175. Early BP. Decelerating self-stimulating and self-injurious behaviors of a student with autism: behavioral intervention in the classroom. Soc Work Educ 1995;17(4):244-55.

176. Eason LJ. Stimulus variation and the acquisition of behavior: a comparison of constant task and varied task teaching methods. J Spec Educ Technol 1983;6(4):5-13.

177. Easterbrooks SR, Handley CM. Behavior change in a student with a dual diagnosis of deafness and pervasive developmental disorder: a case study. Am Ann Deaf 2005;150(5):401-7.

178. Eastridge DD. Using video modeling to teach reciprocal play to a young child with autism [dissertation]. Reno (NV): University of Nevada, Reno; 2003.

179. Eberlin M, McConnachie G, Ibel S, et al. Facilitated communication: a failure to replicate the phenomenon. J Autism Dev Disord 1993;23(3):507-30.

180. Edgerton CL. The effect of improvisational music therapy on the communicative behaviors of autistic children. J Music Ther 1994;31(1):31-62.

181. Edran AF. Picture communication system: a method to support language acquisition to students with moderate to severe disabilities [dissertation]. Long Beach (CA): California State University; 2002.

182. Eikeseth S. Intensive behavioural intervention for children with autism: a reply to Prior. J Paediatr Child Health 2005;41(7):391-2.

183. Eikeseth S, Jahr E. The UCLA reading and writing program: an evaluation of the beginning stages. Res Dev Disabil 2001;22(4):289-307.

184. Elder JH, Valcante G, Yarandi H, et al. Evaluating in-home training for fathers of children with autism using single-subject experimentation and group analysis methods. Nurs Res 2005;54(1):22-32.

185. Eliasoph E, Donnellan AM. A group therapy program for individuals identified as autistic who are without speech and use facilitated communication. Int J Group Psychother 1995;45(4):549-60.

186. Epstein LJ, Taubman MT, Lovaas OI. Changes in self-stimulatory behaviors with treatment. J Abnorm Child Psych 1985;13(2):281-93.

187. Fantuzzo J, Smith C. Linking community-based treatment settings for a disturbed autistic child. Educ Training Ment Retard 1984;19(2):102-7.

188. Ferinden WEJr, Cooper JM. Successful treatment of childhood autism. Language 1973;Speech and Hearing Services in Schools(1973):127-31.

189. Fernandes FD. Language therapy results with adolescents of the autistic spectrum. Profono 2005;17(1):67-76.

190. Fertel-Daly D, Bedell G, Hinojosa J. Effects of a weighted vest on attention to task and self-stimulatory behaviors in preschoolers with pervasive developmental disorders. Am J Occup Ther 2001;55(6):629-40.

191. Fineman KR. Shaping and increasing verbalizations in an autistic child in response to visual-color stimulation. Percept Motor Skills 1968;27(3):1071-4.

192. Fischer I, Glanville BW. Programmed teaching of autistic children: scholastic progress over one year. Arch General Psychiat 1970;23(1):90-4.

193. Foxx RM. The use of a negative reinforcement procedure to increase the performance of autistic and mentally retarded children on discrimination training tasks. Anal Interv Dev Disabil 1984;4(3):253-65.

194. Francke J, Geist EA. The effects of teaching play strategies on social interaction for a child with autism: a case study. J Res Child Educ 2003;18(2):125-40.

195. Fraser DL. A nonverbal intervention for the severely language disordered young child: an intensive approach. 6th Biennial National Training Institute of the National Center for Clinical Infant Programs; Washington, DC.; 1989.

196. Frea WD. Reducing stereotypic behavior by teaching orienting responses to environmental stimuli. J Assoc Pers Severe Hand 1997;22(1): 28-35.

197. Frea WD, Arnold CL, Vittimberga GL. A demonstration of the effects of augmentative communication on the extreme aggressive behavior of a child with autism within an integrated preschool setting . J Pos Behav Interv 2001;3(4):194-8.

198. Fredeen R. Increasing initiations towards peers in children with autism using pivotal response training and collateral gains in quality of initiations [dissertation]. Santa Barbara (CA): University of California; 2005.

199. Freeman KA, Piazza CC. Combining stimulus fading, reinforcement, and extinction to treat food refusal. J Appl Behav Anal 1998;31(4):691-4.

200. Freschi DF, DiLeo PD. Treatment of self-abusive behaviors using positive intervention with an autistic boy. Except Children 1982;49(1):77-8.

201. Friman PC. Effects of punishment procedures on the self-stimulatory behavior of an autistic child. Anal Interv Dev Disabil 1984;4(1): 39-46.

202. Galiatsatos GT, Graff RB. Combining descriptive and functional analyses to assess and treat screaming. Behav Intervent 2003;18(2):123-38.

203. Gamby TE. Remediating generalization deficits in children with autism: an empirical investigation. Dissert Abst Int B: Sciences and Engineering 2002;62(12-B):5962.

204. Ganz JB. Collateral effects of an augmentative communication system on word utterances in children with characteristics of autism [dissertation]. Lawrence (KS): University of Kansas; 2002.

205. Ganz JB, Sigafoos J. Self-monitoring: are young adults with MR and autism able to utilize cognitive strategies independently? Educ Training Dev Disabil 2005;40(1):24-33.

206. Ganz JB, Simpson RL. Effects on communicative requesting and speech development of the picture exchange communication system in children with characteristics of autism. J Autism Dev Disord 2004;34(4):395-409.

207. Garfinkle AN. Using theory-of-mind to increase social competence in young children with autism: a model for praxis in early childhood special education. Dissert Abst Int A: Humanities and Social Sciences 2000;60(7-A):2444.

208. Garfinkle AN, Schwartz IS. Peer imitation: increasing social interactions in children with autism and other developmental disabilities in inclusive preschool classrooms . Top Early Child Spec 2002;22(1):26-38.

209. Garrison-Harrell L, Kamps D, Kravits T. The effects of peer networks on social-communicative behaviors for students with autism. Focus Autism Other Dev Disabil 1997;12(4):241-54.

210. Gaylord-Ross RJ, Haring TG, Breen C, et al. The training and generalization of social interaction skills with autistic youth. J Appl Behav Anal 1984;17(2):229-47.

211. Gena A, Couloura S, Kymissis E. Modifying the affective behavior of preschoolers with autism using in-vivo or video modeling and reinforcement contingencies. J Autism Dev Disord 2005;35(5):545-56 .

212. Gena A, Krantz PJ, McClannahan LE, et al. Training and generalization of affective behavior displayed by youth with autism. J Appl Behav Anal 1996;29(3):291-304.

213. Gerdtz J. Evaluating behavioral treatment of disruptive classroom behaviors of an adolescent with autism. Res Social Work Pract 2000;10(1):98-110.

214. Gerhardt PF, Weiss MJ, Delmolino L. Treatment of severe aggression in an adolescent with autism. Behav Anal Today 2004;4(4):386-94.

215. Gevers C, Clifford P, Mager M, et al. Brief report: a theory-of-mind-based social-cognition training program for school-aged children with pervasive developmental disorders: an open study of its effectiveness. J Autism Dev Dis 2006;36(4):567-71.

216. Gillberg C, Johansson M, Steffenburg, et al. Auditory integration training in children with autism. Autism 1997;1(1):97-100.

217. Girardi A. Predictions of behavioural treatment outcome for young children with autism. Ottawa, (Canada): Carleton University; 2006.

218. Gold AJ. Increasing the number of social initiations during cooperative play in children with autism through the use of a multiple-script-set procedure. Dissert Abst Int B: Sciences and Engineering 2004;64(8-B):4018.

219. Goldstein H, Kaczmarek L, Pennington R, et al. Peer-mediated intervention: attending to, commenting on, and acknowledging the behavior of preschoolers with autism. J Appl Behav Anal 1992;25(2):289-305.

220. Gonzalez-Lopez A, Kamps DM. Social skills training to increase social interactions between children with autism and their typical peers. Focus Autism Other Dev Disabil 1997;12(1):2-14.

221. Gordon R. The effects of contingent versus non-contingent running on the out-of-seat behavior of an autistic boy. Child Fam Behav Ther 1986;8(3):37-44.

222. Graetz JE. Promoting social behavior for adolescents with autism with social stories [dissertation]. Fairfax (VA): George Mason University; 2003.

223. Graziano AM. A group treatment approach to multiple problem behaviors of autistic children. Except Children 1970;36(10):765-70.

224. Green G, Brennan LC, Fein D. Intensive behavioral treatment for a toddler at high risk for autism. Behav Modif 2002;26(1):69-102.

225. Greig A, MacKay T. Asperger's syndrome and cognitive behaviour therapy: new applications for educational psychologists. Educ Child Psychol 2005;22(4):4-15.

226. Grey IM, Honan R, McClean B, et al. Evaluating the effectiveness of teacher training in applied behaviour analysis. J Intell Disabil 2005;9(3):209-27.

227. Grindle CF, Remington B. Teaching children with autism using conditioned cue-value and response-marking procedures: a socially valid procedure. Res Dev Disabil 2004;25(5):413-29.

228. Groden J, Cautela J. Procedures to increase social interaction among adolescents with autism: a multiple baseline analysis. J Behav Ther Exp Psy 1988;19(2):87-93.

229. Gunter P. The reduction of aberrant vocalizations with auditory feedback and resulting collateral behavior change of two autistic boys. Behav Disord 1984;9(4):254-63.

230. Gunter P, Fox JJ, Brady MP, et al. Nonhandicapped peers as multiple exemplars: a generalization tactic for promoting autistic students' social skills. Behav Disord 1988;13(2):116-26.

231. Gunter PL. A case study of the reduction of aberrant, repetitive responses of an adolescent with autism. Educ Treat Child 1993;16(2): 187-97.

232. Hagiwara T. Multimedia social story intervention for students with autism [dissertation]. Lawrence (KS): University of Kansas; 1998.

233. Hagiwara T, Myles BS. A multimedia social story intervention: teaching skills to children with autism. Focus Autism Other Dev Disabil 1999;14(2):82-95.

234. Hagopian LP, Fisher WW, Legacy SM. Schedule effects of noncontingent reinforcement on attention-maintained destructive behavior in identical quadruplets. J Appl Behav Anal 1994;27(2):317-25.

235. Halpern A. A comparison of discrete trial instruction and mand training for teaching children with autism to make requests. Dissert Abst Int B: Sciences and Engineering 2004;65(6-B):3161.

236. Hamilton BL, Snell ME. Using the milieu approach to increase spontaneous communication book use across environments by an adolescent with autism. AAC: Augment Altern Commun 1993;9(4):259-72.

237. Hancock TB, Kaiser AP. The effects of trainer-implemented enhanced milieu teaching on the social communication of children with autism. Top Early Child Spec 2002;22(1):39-54.

238. Handen BL, Apolito PM, Seltzer GB. Use of differential reinforcement of low rates of behavior to decrease repetitive speech in an autistic adolescent. J Behav Ther Exp Psy 1984;15(4):359-64.

239. Handlan S, Bloom LA. The effect of educational curricula and modeling/coaching on the interactions of kindergarten children with their peers with autism. Focus Autistic Behav 1993;8(2):1-11.

240. Handleman JS. Transfer of verbal responses across instructional settings by autistic-type children. J Speech Hear Disord 1981;46(1):69-76.

241. Handleman JS, Harris SL. Generalization from school to home with autistic children. J Autism Dev Disord 1980;10(3):323-33.

242. Harchik AE. Teaching autistic and severely handicapped children to recruit praise: acquisition and generalization. Res Dev Disabil 1990;11(1):77-95.

243. Harchik AE, Luce SC, Harchik AJ, et al. Teaching autistic and severly handicapped children to recruit praise: acquisition and generalization. Res Dev Disabil 1990;11(1):77-95.

244. Hare DJ. The use of cognitive-behavioral therapy with people with asperger syndrome: a case study. Autism 1997;1(2):215-25.

245. Hargrave E, Swisher L. Modifying the verbal expression of a child with autistic behaviors. J Autism Child Schiz 1975;5(2):147-54.

246. Haring TG. The use of differential reinforcement of other behaviors to reduce stereotyped behavior of autistic students during group instruction. The socialization research project: final report. Santa Barbara (CA): San Francisco State University, 1984.

247. Haring TG, Breen CG, Pitts-Conway V, et al. Use of differential reinforcement of other behavior during dyadic instruction to reduce stereotyped behavior of autistic students. Am J Ment Defic 1986;90(6):694-702.

248. Haring TG, Kennedy CH, Adams MJ, et al. Teaching generalization of purchasing skills across community settings to autistic youth using videotape modeling. J Appl Behav Anal 1987;20(1):89-96.

249. Harris SL, Handleman JS, Fong PL. Imitation of self-stimulation: impact on the autistic child's behavior and affect. Child Fam Behav Ther 1987;9(1-2):1-21.

250. Harris SL, Wolchik SA. Suppression of self-stimulation: three alternative strategies. J Appl Behav Anal 1979;12(2):185-98.

251. Harris SL, Wolchik SA, Weitz S. The acquisition of language skills by autistic children: can parents do the job? J Autism Dev Disord 1981;11(4):373-84

252. Harrower JK. A comparison of prompt delivery procedures for increasing spontaneous speech among children with autism. Dissert Abst Int A: Humanities and Social Sciences 2000;60(12-A):4379.

253. Hartley ST, Salzwedel KD. Behavioral writing for an autistic-like child. Acad Ther 1980;16(1):101-10.

254. Hastings RP. Behavioral adjustment of siblings of children with autism engaged in applied behavior analysis early intervention programs: the moderating role of social support. J Autism Dev Disord 2003;33(2):141-50.

255. Hawkins AH. Influencing leisure choices of autisticlike children. J Autism Dev Disord 1982;12(4):359-66.

256. Hawkins DL. Some preliminary and descriptive effects of the natural language paradigm on the spontaneous performance of language and play within a free-play context: three case descriptions. Dissert Abst Int B: Sciences and Engineering 1997;58(3-B):1513.

257. Healey JJ, Ahearn WH, Graft RB, et al. Extended analysis and treatment of self-injurious behavior. Behav Intervent 2001;16(3):181-95.

258. Heckaman KA, Alber S, Hooper S, et al. A comparison of least-to-most prompts and progressive time delay on the disruptive behavior of students with autism. J Behav Educ 1998;8(2): 171-201.

259. Heimann M, Nelson KE, Tjus T, et al. Increasing reading and communication skills in children with autism through an interactive multimedia computer program. J Autism Dev Disord 1995;25(5):459-80.

260. Heitzman-Powell LS. Social skills training for children with asperger's syndrome, high-functioning autism and pervasive developmental disorder - not otherwise specified. Dissert Abst Int B: Sciences and Engineering 2003;64(2-B):984.

261. Helm DP. Behavior modification and intensive interpersonal involvement in the treatment of an autistic child [dissertation]. Chicago (IL): Loyola University of Chicago; 1981.

262. Hendler M, Weisberg P, O'Dell N. Developing the receptive and productive use of pronouns in an autistic child: use of modeling and programming for generalization. Child Fam Behav Ther 1987;9(3-4):17-34.

263. Hendrickson C, Simpson RL. A family style lunch program to aid social development of autistic youth. Teach Except Child 1984;17(1):27-30.

264. Herron ER, Doelling JE, Hawkins SC. Project ACCESS: a statewide project for serving students with autism. Focus Autistic Behav 1989;3(6):16.

265. Hess L. I would like to play but I don't know how: a case study of pretend play in autism. Child Lang Teach Ther 2006;22(1):97-116.

266. Hetzke JD. A sibling-mediated social skills training intervention for children with Asperger's syndrome: results from a pilot study. Dissert Abst Int B: Sciences and Engineering 2004;65(3-B):1548.

267. Hetzroni OE, Shalem U. From logos to orthographic symbols: a multilevel fading computer program for teaching nonverbal children with autism. Focus Autism Other Dev Disabil 2005;20(4):201-12.

268. Hetzroni OE, Tannous J. Effects of a computer-based intervention program on the communicative functions of children with autism. J Autism Dev Disord 2004;34(2):95-113.

269. Hewett FM. Teaching speech to an autistic child through operant conditioning. Am J Orthopsychiat 1965;35(5):927-36.

270. Higbee TS, Carr JE, Patel MR. The effects of interpolated reinforcement on resistance to extinction in children diagnosed with autism: a preliminary investigation. Res Dev Disabil 2002;23(1):61-78.

271. Higgins KC. A comparative study of communication intervention for nonverbal children with autism. Dissert Abst Int B: Sciences and Engineering 1999;60(3-B):1053.

272. Hirsch N, Myles BS. The use of a pica box in reducing pica behavior in a student with autism. Focus Autism Other Dev Disabil 1996;11(4):222-5.

273. Hittner JB. Case study: the combined use of imipramine and behavior modification to reduce aggression in an adult male diagnosed as having autistic disorder. Behav Intervent 1994;9(2):123-39.

274. Hoch H, McComas JJ, Thompson AL, et al. Concurrent reinforcement schedules: behavior change and maintenance without extinction. J Appl Behav Anal 2002;35(2):155-69.

275. Holden EW. The treatment of self-injurious behavior in profoundly retarded autistic children. 30 th Annual Meeting of the Southeastern Psychological Association; New Orleans, LA.; 1984.

276. Hoppe SE. The effects of computer-assisted instruction on communicative interactions and disruptive behavior for individuals with autism. Dissert Abst Int A: Humanities and Social Sciences 2003;64(3-A):796.

277. Horner RH, Budd CM. Acquisition of manual sign use: collateral reduction of maladaptive behavior, and factors limiting generalization. Educ Training Ment Retard 1985;20(1):39-47.

278. Horr AC. Effects of prompting and fading procedures to establish following the line of regard in a child with autism [dissertation]. Denton (TX): University of North Texas; 2004.

279. Houston F. Combined interventions: using social skills training and peer-mediated interventions in an integrated group setting to facilitate the development of social skills in students with autism [dissertation]. Dissert Abst Int 1999;60(3B):1330.

280. Huang C. Scaffolding sight vocabulary acquisition for children with autism using computer-assisted instruction. Dissert Abst Int A: Humanities and Social Sciences 2004;(65):1330-A.

281. Huff JL. The role of sibling support groups on siblings' self-concept and knowledge of autism spectrum disorders. Lawrence (KS): University of Kansas; 2006.

282. Hughes H, Davis R. Treatment of aggressive behavior: the effect of EMG response discrimination biofeedback training. J Autism Dev Disord 1980;10(2):193-202.

283. Hughes V, Wolery MR, Neel RS. Teacher verbalizations and task performance with autistic children. J Autism Dev Disord 1983;13(3):305-16.

284. Hume K, Bellini S, Pratt C. The usage and perceived outcomes of early intervention and early childhood programs for young children with autism spectrum disorder. Top Early Child Spec 2005;25(4):195-207.

285. Hung DW, Cosentino A, Henderson E. Teaching autistic children to follow instructions in a group by a firm physical prompting procedure. J Behav Ther Exp Psy 1979;10(4):329-38.

286. Hungelmann AM. An analysis of teacch-based home programming for young children with autism [dissertation]. Salt Lake City (UT): University of Utah; 2000.

287. Hupp SDA, Reitman D. Parent-assisted modification of pivotal social skills for a child diagnosed with PDD: a clinical replication . J Pos Behav Interv 2000;2(3):183-7.

288. Husted JR, Hall P, Agin B. The effectiveness of time-out in reducing maladaptive behavior of autistic and retarded children. J Psychol 1971;79(2nd half):189-96.

289. Huynen KB, Lutzker JR, Bigelow KM, et al. Planned activities training for mothers of children with developmental disabilities community generalization and follow-up. Behav Modif 1996;20(4):406-27.

290. Hwang B. The effects of social interactive training on early social-communicative skills of preschool children with autism [dissertation]. Nashville (TN): Vanderbilt University; 1998.

291. Hwang B, Hughes C. Effects of social interactive strategies on early social-communicative skills of a preschool child with developmental disabilities. Educ Training Ment Retard Dev Disabil 1995;30(4):336-49.

292. Hwang B, Hughes C. Increasing early social-communicative skills of preverbal preschool children with autism through social interactive training. J Assoc Pers Severe Hand 2000;25(1):18-28.

293. Ihrig K, Wolchik SA. Peer versus adult models and autistic children's learning: acquisition, generalization, and maintenance. J Autism Dev Disord 1988;18(1):67-79.

294. Ingenmey R, Van Houten R. Using time delay to promote spontaneous speech in an autistic child. J Appl Behav Anal 1991;24(3):591-6.

295. Ingersoll B, Dvortcsak A, Whalen C, et al. The effects of a developmental, social-pragmatic language intervention on rate of expressive language production in young children with autistic spectrum disorders. Focus Autism Other Dev Disabil 2005;20(4):213-22.

296. Ingersoll B, Schreibman L, Tran QH. Effect of sensory feedback on immediate object imitation in children with autism. J Autism Dev Disord 2003;33(6):673-83.

297 Ingersoll BR. Teaching children with autism to imitate using a naturalistic treatment approach: effects on imitation, language, play, and social behaviors. Dissert Abst Int B: Sciences and Engineering 2003;63(12-B):6120.

298. Ingvarsson ET. The effects of non-differential reinforcement and differential reinforcement on problem behaviors and accuracy of responding of autistic children [dissertation]. Denton (TX): University of North Texas; 2002.

299. Isaacs JM. The effects of pet-facilitated therapy on the social and interactive behaviors of autistic children [dissertation]. Long Beach (CA): California State University; 1998.

300. Ivey ML, Heflin LJ, Alberto P. The use of social stories to promote independent behaviors in novel events for children with PDD-NOS. Focus Autism Other Dev Disabil 2004;19(3):164-76.

301. Jahr E. Teaching children with autism to answer novel wh-questions by utilizing a multiple exemplar strategy. Res Dev Disabil 2001;22(5):407-23.

302. Jahr E, Eldevik S, Eikeseth S. Teaching children with autism to initiate and sustain cooperative play. Res Dev Disabil 2000;21(2):151-69.

303. Jeffreys C. Using video modeling to teach complex play sequences to children with autism [dissertation]. Denton (TX): University of North Texas; 2005.

304. Jennett HK. The effects of discrete trial instruction and mand training for teaching children with autism to make requests for items. Dissert Abst Int B: Sciences and Engineering 2005;66(1-B):556.

305. Jennische M. Alternative and augmentative communication routes used by a nonspeaking autistic boy. 5th Biennial Conference of the International Society for Augmentative and Alternative Communication; Anaheim, CA.; 1998.

306. Jensen CC, McConnachie G, Pierson T. Long-term multicomponent intervention to reduce severe problem behavior: a 63-month evaluation . J Pos Behav Interv 2001;3(4):225-36, 250.

307. Jensen V, Haycook T, Sinclair L, et al. Efficacy of applied behavior analysis in reducing maladaptive behavior in shool-aged children with autism: one-year outcome data for behavior reduction plans. J Dev Behav Pediatr 2003;24(5):393.

308. Jenson WR, Rovner L, Cameron S, et al. Reduction of self-injurious behavior in an autistic girl using a multifaceted treatment program. J Behav Ther Exp Psy 1985;16(1):77-80.

309. Johnson MR, Whitman TL, Barloon-Noble R. A home-based program for a preschool behaviorally disturbed child with parents as therapists. J Behav Ther Exp Psy 1978;9(1):65-70.

310. Johnston S, Nelson C, Evans J, et al. The use of visual supports in teaching young children with autism spectrum disorder to initiate interactions. AAC: Augment Altern Commun 2003;19(2):86-103.

311. Jones CM. Using the picture exchange communication system and time delay to enhance the spontaneous speech of children with autism. Dissert Abst Int B: Sciences and Engineering 2005;65(8-B):4270.

312. Jones EA. Joint attention intervention for children with autism. Dissert Abst Int B: Sciences and Engineering 2003;64(4-B):1904.

313. Jones EA, Carr EG, Feeley KM. Multiple effects of joint attention intervention for children with autism. Behav Modif 2006;30(6):782-834.

314. Jones FH. An extinction procedure for eliminating self-destructive behavior in a 9-year-old autistic girl. J Autism Child Schiz 1974;4(3):241-50.

315. Jones J G, Best JF. Movement of therapy in the treatment of autistic children: II Intellectual changes. Aust Occup Ther J 1975;22(1):15-29.

316. Josefi O, Ryan V. Non-directive play therapy for young children with autism: a case study. Clin Child Psychol Psychiatry 2004;9(4):533-51.

317. Kaiser AP, Hancock TB, Nietfeld JP. The effects of parent-implemented enhanced milieu teaching on the social communication of children who have autism. Early Educ Dev 2000;11(4):423-46.

318. Kamps D. A comparison of instructional arrangements for children with autism served in a public school setting. Educ Treat Child 1990;13(3):197-215.

319. Kamps D. Enhanced small group instruction using choral responding and student interaction for children with autism and developmental disabilities. Am J Ment Retard 1994;99(1):60-73.

320. Kamps DM, Barbetta PM, Leonard BR, et al. Classwide peer tutoring: an integration strategy to improve reading-skills and promote peer interactions among students with autism and general-education peers. J Appl Behav Anal 1994;27(1):49-61.

321. Kamps DM, Leonard BR, Vernon S, et al. Teaching social skills to students with autism to increase peer interactions in an integrated first-grade classroom. J Appl Behav Anal 1992;25(2):281-8.

322. Kamps DM, Potucek J, Lopez AG, et al. The use of peer networks across multiple settings to improve social interaction for students with autism. J Behav Educ 1997;7(3):335-57.

323. Kane A, Luiselli JK, Dearborn S, et al. Wearing a weighted vest as intervention for children with autism/pervasive developmental disorder: behavioral assessment of stereotypy and attention to task. Sci Rev Ment Health Pract 2004;3(2):19-24.

324. Kaplan RS, Steele AL. An analysis of music therapy program goals and outcomes for clients with diagnoses on the autism spectrum. J Music Ther 2005;42(1):2-19.

325. Karmali IAL. Reducing palilalia and echolalia by teaching the tact operant to young children with autism. Dissert Abst Int B: Sciences and Engineering 2000;61(6-B):3265.

326. Kashinath SP. Effects of enhancing the generalized use of teaching strategies by caregivers of children with autism. Dissert Abst Int B: Sciences and Engineering 2003;(63):5797-B.

327. Kay S, Harchik AE, Luiselli JK. Elimination of drooling by an adolescent student with autism attending public high school. J Pos Behav Interv 2006;8(1):24-8.

328. Kean JM. The development of social skills in autistic twins. New Zeal Med J 1975;81(534):204-7.

329. Keeling K, Myles BS, Gagnon E, et al. Using the power card strategy to teach sportsmanship skills to a child with autism. Focus Autism Other Dev Disabil 2003;18(2):105-11.

330. Keen D, Rodger S, Doussin K, et al. A pilot study of the effects of a social-pragmatic intervention on the communication and symbolic play of children with autism. Autism 2007;11(1):63-71.

331. Keen D, Sigafoos J, Woodyatt G. Replacing prelinguistic behaviors with functional communication. J Autism Dev Disord 2001;31(4):385-98.

332. Kenzer AL. A comparison of discrete trial instruction and fluency instruction on retention of academic tasks with young children with autism [dissertation]. Reno (NV): University of Nevada, Reno; 2004.

333. Kern L, Carberry N, Haidara C. Analysis and intervention with two topographies of challenging behavior exhibited by a young woman with autism. Res Dev Disabil 1997;18(4):275-87.

334. Kern L, Koegel RL, Dunlap G. The influence of vigorous versus mild exercise on autistic stereotyped behaviors. J Autism Dev Disord 1984;14(1):57-67.

335. Kern L, Marder TJ. A comparison of simultaneous and delayed reinforcement as treatments for food selectivity. J Appl Behav Anal 1996;29(2):243-6.

336. Kerr S, Cobb S. Asperger's syndrome interactive: development and use of virtual environments for social skills training. Cyberpsychol Behav 2005;8(4):330-1.

337. Keyworth PLW. The effects of social stories on the social interaction of students with autism [dissertation]. Iowa City (IA): University of Iowa; 2004.

338. Kim J. Effects of a parent's intervention to decrease stereotypic behavior and increase interactions using self-management treatment for students with autism in Korea [dissertation]. Salt Lake City (UT): Utah State University; 1996.

339. Kim U. The effects of milieu teaching procedures on the spoken language skills of children with autism [dissertation]. Austin (TX): The University of Texas; 2000.

340. Kinney EM, Vedora J, Stromer R. Computer-presented video models to teach generative spelling to a child with an autism spectrum disorder. J Pos Behav Interv 2003;5(1):22-9.

341. Kirchner TA. Do the communicative acts performed by children with autism in a classroom setting vary when peers with typical development are participating in the class? [dissertation]. Pittsburg (PA): Duquesne University; 2001.

342. Klecan-Aker JS, Gill C. Teaching language organization to a child with pervasive developmental disorder: a case study. Child Lang Teach Ther 2005;21(1):60-74.

343. Knapp VM. An analysis of the role of attention in a behavioral treatment of autism [dissertation]. Dissert Abst Int 2002;62(10B):4769.

344. Ko C. Teaching anger management to children with pervasive development disorders: the effect of computer-assisted instruction. Dissert Abst Int A: Humanities and Social Sciences 2003;63(7-A):2504.

345. Koegel LK, Camarata SM, Valdez-Menchaca M, et al. Setting generalization of question-asking by children with autism. Am J Ment Retard 1998;102(4):346-57.

346. Koegel LK, Carter CM, Koegel RL. Teaching children with autism self-initiations as a pivotal response. Top Lang Disord 2003;23(2):134-45.

347. Koegel LK, Koegel RL, Hurley C, et al. Improving social skills and disruptive behavior in children with autism through self-management. J Appl Behav Anal 1992;25(2):341-53.

348. Koegel LK, Koegel RL, Shoshan Y, et al. Pivotal response intervention II: preliminary long-term outcomes data. J Assoc Pers Severe Hand 1999;24(3):186-98.

349. Koegel LK, Stiebel D, Koegel RL. Reducing aggression in children with autism toward infant or toddler siblings. J Assoc Pers Severe Hand 1998;23(2):111-8.

350. Koegel RI, Frea WD. Treatment of social behavior in autism through the modification of pivotal social skills. J Appl Behav Anal 1993;26(3):369-77.

351. Koegel RL, Camarata S, Koegel LK, et al. Increasing speech intelligibility in children with autism. J Autism Dev Disord 1998;28(3):241-51.

352. Koegel RL, Dunlap G, Dyer K. Intertrial interval duration and learning in autistic children. J Appl Behav Anal 1980;13(1):91-9.

353. Koegel RL, Firestone PB, Kramme KW, et al. Increasing spontaneous play by suppressing self-stimulation in autistic children. J Appl Behav Anal 1974;7(4):521-8.

354. Koegel RL, Glahn TJ, Nieminen GS. Generalization of parent-training results. J Appl Behav Anal 1978;11(1):95-109.

355. Koegel RL, Koegel LK. Extended reductions in stereotypic behavior of students with autism through a self-management treatment package. J Appl Behav Anal 1990;23(1):119-27.

356. Koegel RL, Koegel LK, Surratt A. Language intervention and disruptive behavior in preschool children with autism. J Autism Dev Disord 1992;22(2):141-53.

357. Koegel RL, O'Dell M, Dunlap G. Producing speech use in nonverbal autistic children by reinforcing attempts. J Autism Dev Disord 1988;18(4):525-38.

358. Koegel RL, O'Dell MC, Koegel LK. A natural language teaching paradigm for nonverbal autistic children. J Autism Dev Disord 1987;17(2):187-200.

359. Koegel RL, Openden D, Koegel LK. A systematic desensitization paradigm to treat hypersensitivity to auditory stimuli in children with autism in family contexts. Res Pract Pers Sev Disabil 2004;29(2):122-34.

360. Koegel RL, Symon JB, Kern Koegel L. Parent education for families of children with autism living in geographically distant areas. J Pos Behav Interv 2002;4(2):88-103.

361. Koegel RL, Werner GA, Vismara LA, et al. The effectiveness of contextually supported play date interactions between children with autism and typically developing peers. Res Pract Pers Sev Disabil 2005;30(2):93-102.

362. Kohler FW, Anthony LJ, Steighner SA, et al. Teaching social interaction skills in the integrated preschool: an examination of naturalistic tactics. Top Early Child Spec 2001;21(2):93-103.

363. Kohler FW, Strain PS, Hoyson M, et al. Using a group-oriented contingency to increase social interactions between children with autism and their peers: a preliminary analysis of corollary supportive behaviors. Behav Modif 1995;19(1):10-32.

364. Kohler FW, Strain PS, Hoyson M, et al. Merging naturalistic teaching and peer-based strategies to address the IEP objectives of preschoolers with autism: an examination of structural and child behavior outcomes. Focus Autism Other Dev Disabil 1997;12(4):196-206.

365. Kohler FW, Strain PS, Maretsky S, et al. Promoting positive and supportive interactions between preschoolers: an analysis of group-oriented contingencies. J Early Interv 1990;14(4):327-41.

366. Koita H, Sonoyama S. Communication training using the Picture Exchange Communication System (PECS): case study of a child with autistic disorder [japanese]. Jpn J Behav Anal 2004;19(2):161-74.

367. Kok AJ, Kong TY, Bernard-Opitz V. A comparison of the effects of structured play and facilitated play approaches on preschoolers with autism: a case study. Autism 2002;6(2):181-96.

368. Konstantareas MM, Oxman J, Webster CD. Simultaneous communication with autistic and other severely dysfunctional nonverbal children. J Commun Disord 1977;10(3):267-82.

369. Koppenhaver DA, Erickson KA. Natural emergent literacy supports for preschoolers with autism and severe communication impairments. Top Lang Disord 2003;23(4):283-92.

370. Kostka MJ. A comparison of selected behaviors of a student with autism in special education and regular music classes. Music Ther Perspect 1993;11(2):57-60.

371. Krantz PJ, MacDuff MT, McClannahan LE. Programming participation in family activities for children with autism: parents' use of photographic activity schedules. J Appl Behav Anal 1993;26(1):137-8.

372. Krantz PJ, McClannahan LE. Social interaction skills for children with autism: a script-fading procedure for beginning readers. J Appl Behav Anal 1998;31(2):191-202.

373. Krantz PJ, McClannahan LE. Teaching children with autism to initiate to peers: effects of a script-fading procedure. J Appl Behav Anal 1993;26(1):121-32 .

374. Krantz PJ, Zalenski S, Hall JJ, et al. Teaching complex language to autistic children. Anal Interv Dev Disabil 1981;1(3-4):259-97.

375. Kravits TR, Kamps DM, Kemmerer K, et al. Brief report: increasing communication skills for an elementary-aged student with autism using the picture exchange communication system. J Autism Dev Disord 2002;32(3):225-30.

376. Kreimeyer KH. A comparison of the effects of speech training, modeled sign language training and prompted sign language training on the language behavior of autistic preschool children. Dissert Abst Int 1985;46(3-B):980-1.

377. Kroeger KA, Nelson WM III. A language programme to increase the verbal production of a child dually diagnosed with Down syndrome and autism. J Intell Disabil Res 2006;50(2):101-8.

378. Kuoch H, Mirenda P. Social story interventions for young children with autism spectrum disorders. Focus Autism Other Dev Disabil 2003;18(4):219-27.

379. Kuttler S, Myles BS, Carlson JK. The use of social stories to reduce precursors to tantrum behavior in a student with autism. Focus Autism Other Dev Disabil 1998;13(3):176-82.

380. Kyparissos N. Extending conversations among adolescent peers with autism. Dissert Abst Int B: Sciences and Engineering 1997;57(10-B):6551.

381. LaDeane C. Development of communicative behavior in autistic children: a parent program using signed speech. Devereux Forum 1977;12(1):1-15.

382. Lalli JS. Identification and modification of a response-class hierarchy. J Appl Behav Anal 1995;28(4):551-9.

383. Lantz JF. Using video self-modeling to increase the prosocial behavior of children with autism and their siblings. Dissert Abst Int B: Sciences and Engineering 2005;66(2-B):1175.

384. Larkin AS, Gurry S. Progress reported in three children with autism using daily life therapy. J Autism Dev Disord 1998;28(4):339-42.

385. Lasater MW, Brady MP. Effects of video self-modeling and feedback on task fluency: a home-based intervention. Educ Treat Child 1995;18(4):389-407.

386. Laski KE, Charlop MH, Schreibman L. Training parents to use the natural language paradigm to increase their autistic children's speech. J Appl Behav Anal 1988;21(4):391-400.

387. Laushey KM. Using a concept mastery routine to teach social skills to elementary children with high functioning autism in order to facilitate acceptance [dissertation]. Atlanta (GA): Georgia State University; 2002.

388. Laushey KM, Heflin LJ. Enhancing social skills of kindergarten children with autism through the training of multiple peers as tutors. J Autism Dev Disord 2000;30(3):183-93.

389. Law J, Dockrell JE, Castelnuovo E, et al. Early years centres for pre-school children with primary language difficulties: what do they cost, and are they cost-effective? Int J Lang Comm Dis 2006;41(1):67-81.

390. Le L. Increasing vocabulary through question-asking for children with autism. Dissert Abst Int B: Sciences and Engineering 2002;(63):2569B.

391. Leahy C, Rarig M, Chambliss C. Addressing the needs of children with autism: an evaluation of a gymnastics program for children with autism and related disorders. Available at: http://eric.ed.gov/ERICWebPortal/custom/portlets/recordDetails/detailmini.jsp?_nfpb=true&_&ERICExtSearch_SearchValue_0=ED445437&ERICExtSearch_SearchType_0=eric_accno&accno=ED445437. Accessed: May, 2007

392. LeBlanc LA, Carr JE, Crossett SE, et al. Intensive outpatient behavioral treatment of primary urinary incontinence of children with autism. Focus Autism Other Dev Disabil 2005;20(2):98-105.

393. LeGoff DB. Use of LEGO as a therapeutic medium for improving social competence. J Autism Dev Disord 2004; 34(5):557-71.

394. Lee R, McComas JJ, Jawor J. The effects of differential and lag reinforcement schedules on varied verbal responding by individuals with autism. J Appl Behav Anal 2002;35(4):391-402.

395. Lefebvre D, Strain PS. Effects of a group contingency on the frequency of social interactions among autistic and nonhandicapped preschool children: making LRE efficacious. J Early Interv 1989;13(4):329-41.

396. Lerman DC, Kelley ME, Vorndran CM, et al. Collateral effects of response blocking during the treatment of stereotypic behavior. J Appl Behav Anal 2003;36(1):119-23.

397. Lerman DC, Swiezy N, Perkins-Parks S, et al. Skill acquisition in parents of children with developmental disabilities: interaction between skill type and instructional format. Res Dev Disabil 2000;21(3):183-96.

398. Leventhal-Belfer L, Coe C. The Friends Program: a therapeutic group program for preschool children with Aspergers syndrome and their parents. Inf Mental Hlth J 2000;21(4-5):378.

399. Levi A. The effect of the miller method on attention, emotional reactiveness, and sensory seeking in school-aged children with autism/pdd as measured by the sensory profile [dissertation]. New York (NY): Touro College; 2001.

400. Levidioti M. The effects of structured teaching on stereotypic, on-task, and off-task behaviors of children with autism spectrum disorders in physical education [dissertation]. Montreal (QC): Mcgill University (Canada); 2004.

401. Lewis L, Trushell J, Woods P. Effects of ICT group work on interactions and social acceptance of a primary pupil with Asperger's syndrome. Brit J Educ Technol 2005;36(5):739-55.

402. Lifter K, Ellis J, Cannon B, et al. Developmental specificity in targeting and teaching play activities to children with pervasive developmental disorders. J Early Interv 2005;27(4):247-67.

403. Linderman TM, Stewart KB. Sensory integrative-based occupational therapy and functional outcomes in young children with pervasive developmental disorders: a single-subject study. Am J Occup Ther 1999;53(2):207-13.

404. Link HM. Auditory Integration Training (AIT): sound therapy? case studies of three boys with autism who received AIT. Br J Learn Disabil 1997;25(3):106-10.

405. Loncola JA. Teaching social communication skills to children with autism. Dissert Abst Int A: Humanities and Social Sciences 2004;(65):892-A.

406. Lorimer PA, Simpson RL, Myles BS, et al. The use of social stories as a preventative behavioral intervention in a home setting with a child with autism. J Pos Behav Interv 2002;4(1):53-60.

407. Lovaas OI, Koegel R, Simmons JQ, et al. Some generalization and follow-up measures on autistic children in behavior therapy. J Appl Behav Anal 1973;6(1):131-66.

408. Luce SC, Delquadri J, Hall RV. Contingent exercise: a mild but powerful procedure for suppressing inappropriate verbal and aggressive behavior. J Appl Behav Anal 1980;13(4):583-94.

409. Luiselli JK. Application of immobilization time-out in management programming with developmentally disabled children. Child Fam Behav Ther 1984;6(1):1-15.

410. Luiselli JK. Case demonstration of a fading procedure to promote school attendance of a child. J Posit Behav Interv 2000;2(1):47.

411. Luiselli JK. Treatment of an autistic childs fear of riding a school bus through exposure and reinforcement. J Behav Ther Exp Psy 1978;9(2):169-72.

412. Luiselli JK, Reisman J, Helfen CS, et al. Control of self-stimulatory behavior of an autistic child through brief physical restraint. Sch Appl Learning Theor 1976;9(2):3-13.

413. Luiselli JK, Ricciardi JN, Gilligan K. Liquid fading to establish milk consumption by a child with autism. Behav Intervent 2005;20(2):155-63.

414. Luiselli JK, Ricciardi JN, Zubow M, et al. Practice of an alternative behavior as intervention for object stereotypy: comparison of contingent and noncontingent implementation across evoking stimuli. 2004;5(3):304-12.

415. Luiselli JK, Suskin L, McPhee DF. Continuous and intermittent application of overcorrection in a self-injurious autistic child: alternating treatments design analysis. J Behav Ther Exp Psy 1981;12(4):355-8.

416. Luiselli JK, Wolongevicz J, Egan P, et al. The Family Support Program: description of a preventive, community-based behavioral intervention for children with pervasive developmental disorders. Child Fam Behav Ther 1999;21(1):1-18.

417. Lynch S. Intensive behavioural intervention with a 7-year-old girl with autism. Autism 1998;2(2):181-97.

418. Maag JW. Sensory extinction and overcorrection in suppressing self-stimulation: a preliminary comparison of efficacy and generalization. Educ Treat Child 1986;9(3):189-201.

419. Maag JW, Rutherford RB, Wolchik SA, et al. Comparison of two short overcorrection procedures on the stereotypic behavior of autistic children. J Autism Dev Disord 1986;16(1):83-7.

420. MacArthur J, Ballard KD, Artinian M. Teaching independent eating to a developmentally handicapped child showing chronic food refusal and disruption at mealtimes. Aust NZ J Dev Disab 1986;12(3):203-10.

421. MacDonald R, Clark M, Garrigan E, et al. Using video modeling to teach pretend play to children with autism. Behav Intervent 2005;20(4):225-38.

422. MacDuff GS, Krantz PJ, McClannahan LE. Teaching children with autism to use photographic activity schedules: maintenance and generalization of complex response chains. J Appl Behav Anal 1993;26(1):89-97.

423. Maddox CL. Effect of therapist-initiated versus self-initiated vestibular stimulation on vocalization in children with autism [dissertation]. Kalamazoo (MI): Western Michigan University; 1990.

424. Magiati I, Howlin P. A pilot evaluation study of the picture exchange communication system (PECS) for children with autistic spectrum disorders. Autism 2003;7(3):297-320.

425. Mahlberg M. Music therapy in the treatment of an autistic child. J Music Ther 1973;10(4):189-93.

426. Mahoney G, Perales F. Relationship-focused early intervention with children with pervasive developmental disorders and other disabilities: a comparative study. J Dev Behav Pediatr 2005;26(2):77-85.

427. Mahoney G, Perales F. Using relationship-focused intervention to enhance the social-emotional functioning of young children with autism spectrum disorders. Top Early Child Spec 2003;23(2):77-89.

428. Maione L, Mirenda P. Effects of video modeling and video feedback on peer-directed social language skills of a child with autism. J Pos Behav Interv 2006;8(2):106-18.

429. Mancina C, Tankersley M, Kamps D, et al. Brief report: reduction of inappropriate vocalizations for a child with autism using a self-management treatment program. J Autism Dev Disord 2000;30(6):599-606.

430. Marckel JM, Neef NA, Ferreri SJ. A preliminary analysis of teaching improvisation with the picture exchange communication system to children with autism. J Appl Behav Anal 2006;39(1):109-15.

431. Marholin D, Townsend NM. An experimental analysis of side effects and response maintenance of a modified overcorrection procedure: the case of the persistent twiddler. Behav Ther 1978;9(3):383-90.

432. Marshall NR, Hegrenes JR. Programmed communication therapy for autistic mentally retarded children. J Speech Hear Disord 1970;35(1):70-83.

433. Martin CA, Drasgow E, Halle JW, et al. Teaching a child with autism and severe language delays to reject: direct and indirect effects of functional communication training. Educ Psychol 2005;25(2-3):287-304.

434. Martin F, Farnum J. Animal-assisted therapy for children with pervasive developmental disorders. West J Nurs Res 2002;24(6):657-70.

435. Martin GL, England G, Kaprowy E, et al. Operant conditioning of kindergarten-class behavior in autistic children. Behav Res Ther 1968;6(3):281-94.

436. Massey NG, Wheeler JJ. Acquisition and generalization of activity schedules and their effects on task engagement in a young child with autism in an inclusive pre-school classroom. Educ Training Ment Retard Dev Disabil 2000;35(3):326-35 .

437. Matson JL, Box ML, Francis KL. Treatment of elective mute behavior in two developmentally delayed children using modeling and contingency management. J Behav Ther Exp Psy 1992;23(3):221-9.

438. Matson JL, Sevin JA, Box ML, et al. An evaluation of two methods for increasing self-initiated verbalizations in autistic children. J Appl Behav Anal 1993;26(3):389-98.

439. Matson JL, Sevin JA, Fridley D, et al. Increasing spontaneous language in three autistic children. J Appl Behav Anal 1990;23(2):227-33.

440. Matson JL, Taras ME, Sevin JA, et al. Teaching self-help skills to autistic and mentally retarded children. Res Dev Disabil 1990;11(4):361-78.

441. Mattson BL. The effectiveness of negotiated social communication activities with children with autism. Dissert Abst Int 1993;54(6-A):2116.

442. Mccarthy DS. Increasing sight word vocabulary among high-functioning autistic students through the use of computer-assisted instruction software [dissertation]. Dominguez Hills (CA): California State University; 1999.

443. McComas J, Hoch H, Paone D, et al. Escape behavior during academic tasks: a preliminary analysis of idiosyncratic establishing operations. J Appl Behav Anal 2000;33(4):479-93.

444. Mcdonald ME. Effects of a treatment package on the creative play behavior of children with autism. Dissert Abst Int B: Sciences and Engineering 2004;65(3-B):1534.

445. McDonald ME, Hemmes NS. Increases in social initiation toward an adolescent with autism: reciprocity effects. Res Dev Disabil 2003;24(6):453-65.

446. Mcevoy MA. A comparison of two procedures for teaching language to autistic children (incidental teaching). Knoxville (TN): University of Tennessee; 1984.

447. McEvoy MA, Brady MP. Contingent access to play materials as an academic motivator for autistic and behavior disordered children. Educ Treat Child 1988;11(1):5-18.

448. McEvoy MA, Nordquist VM, Twardosz S, et al. Promoting autistic children's peer interaction in an integrated early childhood setting using affection activities. J Appl Behav Anal 1988;21(2):193-200.

449. Mcgee GG, Almeida MC, Sulzerazaroff B, et al. Promoting reciprocal interactions via peer incidental teaching. J Appl Behav Anal 1992;25(1):117-26.

450. McGee GG, Krantz PJ, Mason D, et al. A modified incidental-teaching procedure for autistic youth: acquisition and generalization of receptive object labels. J Appl Behav Anal 1983;16(3):329-38.

451. McGee GG, Krantz PJ, McClannahan LE. Conversational skills for autistic adolescents: teaching assertiveness in naturalistic game settings. J Autism Dev Disord 1984;14(3):319-30.

452. McGee GG, Krantz PJ, McClannahan LE. The facilitative effects of incidental teaching on preposition use by autistic children. J Appl Behav Anal 1985;18(1):17-31.

453. McGrath AM, Bosch S, Sullivan CL, et al. Training reciprocal social interactions between preschoolers and a child with autism. J Pos Behav Interv 2003;5(1):47-54.

454. McHale SM, Olley JG, Marcus LM, et al. Nonhandicapped peers as tutors for autistic children. Except Children 1981;48(3):263-5.

455. McHale SM, Simeonsson RJ, Marcus LM, et al. The social and symbolic quality of autistic children's communication. J Autism Dev Disord 1980;10(3):299-310.

456. McKeegan GF, Estill K, Campbell BM. Use of nonexclusionary timeout for the elimination of a stereotyped behavior. J Behav Ther Exp Psy 1984;15(3):261-4.

457. McNerney EK. Videotape communication between school and clinic and the effects on teacher behavior and generalization of expressive language to classroom settings for children with autism. Dissert Abst Int 2003;(64):2929B.

458. Mechling LC, Gast DL, Cronin BA. The effects of presenting high-preference items, paired with choice, via computer-based video programming on task completion of students with autism. Focus Autism Other Dev Disabil 2006;21(1):7-13.

459. Meyer LH, Fox A, Schermer A, et al. The effects of teacher intrusion on social play interactions between children with autism and their nonhandicapped peers. J Autism Dev Disord 1987;17(3):315-32.

460. Miller N. Language therapy with an autistic nonverbal boy. Except Children 1969;35(7):555-7.

461. Miller N, Neuringer A. Reinforcing variability in adolescents with autism. J Appl Behav Anal 2000;33(2):151-65.

462. Milstein JP. An assessment of video modeling procedures for teaching autistic children self-help skills. Dissert Abst Int 1995;56(3A):0871.

463. Miranda-Linne F, Melin L. Acquisition, generalization, and spontaneous use of color adjectives: a comparison of incidental teaching and traditional discrete-trial procedures for children with autism. Res Dev Disabil 1992;13(3):191-210.

464. Mirenda PL. The effects of adult interaction style on verbal conversational behavior in adolescents with autism or retardation. Dissert Abst Int 1985;45(8-A):2486-7.

465. Mirenda PL, Donnellan AM. Effects of adult interaction style on conversational behavior in students with severe communication problems. Lang Speech Hear Serv Sch 1986;17(2):126-41.

466. Moes DR. Providing opportunities to make choices to facilitate the homework performance of children with autism [dissertation]. Santa Barbara (CA): University of California; 1996.

467. Moes DR, Frea WD. Contextualized behavioral support in early intervention for children with autism and their families. J Autism Dev Disord 2002;32(6):519-33.

468. Moes DR, Frea WD. Using family context to inform intervention planning for the treatment of a child with autism. J Pos Behav Interv 2000;2(1):40-6.

469. Moran DR, Whitman TL. Developing generalized teaching skills in mothers of autistic children. Child Fam Behav Ther 1991;13(1):13-37.

470. Morrison K. Teaching children with autism to make eye contact following cues other than "look at me". Dissert Abst Int B: Sciences and Engineering 2000;61(2-B):1066.

471. Morrison RS, Sainato DM, Benchaaban D, et al. Increasing play skills of children with autism using activity schedules and correspondence training. J Early Interv 2002;25(1):58-72.

472. Mukaddes NM, Kaynak FN, Kinali G, et al. Psychoeducational treatment of children with autism and reactive attachment disorder. Autism 2004;8(1):101-9.

473. Mullins JL, Christian L. The effects of progressive relaxation training on the disruptive behavior of a boy with autism. Res Dev Disabil 2001;22(6):449-62 .

474. Mundschenk NA, Sasso GM. Assessing sufficient social exemplars for students with autism. Behav Disord 1995;21(1):62-78.

475. Myles BS, Simpson RL. Facilitated communication with children diagnosed as autistic in public school settings. Psychol Schools 1994;31(3):208-20.

476. Myles BS, Simpson RL, Smith SM. Collateral behavioral and social effects of using facilitated communication with individuals with autism. Focus Autism Other Dev Disabil 1996;11(3):163-9, 190.

477. Myles BS, Simpson RL, Smith SM. Impact of facilitated communication combined with direct instruction on academic performance of individuals with autism. Focus Autism Other Dev Disabil 1996;11(1):37-44.

478. Najdowski AC, Wallace MD, Doney JK, et al. Parental assessment and treatment of food selectivity in natural settings. J Appl Behav Anal 2003;36(3):383-6.

479. Nakamura K, Iwahashi K, Fukunishi I, et al. Social skills training for a case of Savant syndrome and Asperger's syndrome. Aust NZ J Psychiatr 1998;32(4):697.

480. Nanclares V. Program evaluation: an analysis of the puentes intensive home-based treatment program for young children with autism. Dissert Abst Int B: Sciences and Engineering 2004;65(6-B):3173.

481. Narayan J, Chakravarti SN, David J, et al. Analysis of educational support systems for children with mental retardation and autism spectrum disorders. Int J Rehabil Res 2005;28(4):365-8.

482. Neidert PL, Iwata BA, Dozier CL. Treatment of multiply controlled problem behavior with procedural variations of differential reinforcement. Exceptionality 2005;13(1):45-53.

483. Neisworth JT, Wert BY. Videotaped self-modeling as a technique for training preschoolers with autism in social-communicative functioning [dissertation]. Lincoln University (PA): Pennsylvania State University; 2002.

484. Neitzel JC. Understanding parent and professional satisfaction with family-centered early intervention services for young children with autism [dissertation]. Chapel Hill (NC): University of North Carolina; 2004.

485. Nelson CB. Keys to play: a strategy to increase the social interactions of young children with autism and their typically developing peers [dissertation]. Salt Lake City (UT): University of Utah; 2004.

486. Neufeld A, Fantuzzo JW. Contingent application of a protective device to treat the severe self-biting behavior of a disturbed autistic child. J Behav Ther Exp Psy 1984;15(1):79-83.

487. Newman B, Buffington DM, Hemmes NS. Self-reinforcement used to increase the appropriate conversation of autistic teenagers. Educ Training Ment Retard Dev Disabil 1996;31(4):304-9.

488. Newman B, Buffington DM, O'Grady MA, et al. Self-management of schedule following in three teenagers with autism. Behav Disord 1995;20(3):190-6.

489. Newman B, Needelman M, Reinecke DR, et al. The effect of providing choices on skill acquisition and competing behavior of children with autism during discrete trial instruction. Behav Intervent 2002;17(1):31-41.

490. Newman B, Reinecke DR, Meinberg DL. Self-management of varied responding in three students with autism. Behav Intervent 2000;15(2):145-51.

491. Newman B, Tuntigian L, Ryan CS, et al. Self-management of a DRO procedure by three students with autism. Behav Intervent 1997;12(3):149-56.

492. Ney PG. Effect of contingent and non-contingent reinforcement on the behavior of an autistic child. J Autism Child Schiz 1973;3(2):115-27.

493. Neysmith-Roy JM. The Tomatis method with severely autistic boys: individual case studies of behavioural changes. S Afr JPsychol 2001;31(1):19-28.

494. Nicoll JM. Using a single-subject design to assess the validity of facilitated communication under naturalistic conditions [dissertation]. Philadelphia (PA): Temple University; 1997.

495. Nikopoulos CK, Keenan M. Effects of video modeling on social initiations by children with autism. J Appl Behav Anal 2004;37(1):93-6.

496. Nikopoulos CK, Keenan M. Promoting social initiation children with autism using video modeling . Behav Intervent 2003;18(2):87-108.

497. Nordquist VM, Twardosz S, McEvoy MA. Effects of environmental reorganization in classrooms for children with autism. J Early Interv 1991;15(2):135-52.

498. Nordquist VM, Wahler RG. Naturalistic treatment of an autistic child. J Appl Behav Anal 1973;6(1):79-87.

499. Norris C, Dattilo J. Evaluating effects of a social story intervention on a young girl with autism. Focus Autism Other Dev Disabil 1999;14(3):180-6.

500. Nuzzolo-Gomez R, Greer RD. Emergence of untaught mands or tacts of novel adjective-object pairs as a function of instructional history. Anal Verbal Behav 2004;20:63-76.

501. O'Connor IM, Klein PD. Exploration of strategies for facilitating the reading comprehension of high-functioning students with autism spectrum disorders. J Autism Dev Disord 2004;34(2):115-27.

502. O'Loughlin RA. Facilitating prelinguistic communication skills of attention by integrating a music stimulus within typical language intervention with autistic children. Dissert Abst Int A: Humanities and Social Sciences 2000;(61):947A.

503. O'Neill RE, Sweetland-Baker M. Brief report: an assessment of stimulus generalization and contingency effects in functional communication training with two students with autism. J Autism Dev Disord 2001;31(2):235-40.

504. O'Reilly M, Sigafoos J, Lancioni G, et al. An examination of the effects of a classroom activity schedule on levels of self-injury and engagement for a child with severe autism. J Autism Dev Disord 2005;35(3):305-11.

505. Odom SL, Strain PS. A comparison of peer-initiation and teacher-antecedent interventions for promoting reciprocal social interaction of autistic preschoolers. J Appl Behav Anal 1986;19(1):59-71.

506. Odom SL, Watts E. Reducing teacher prompts in peer-mediated interventions for young children with autism. J Spec Educ 1991;25(1):26-43.

507. Ogletree BT. Communication intervention for a preverbal child with autism: a case study. Focus Autistic Behav 1992;7(1):1-12.

508. Oke NJ, Schreibman L. Training social initiations to a high-functioning autistic child: assessment of collateral behavior change and generalization in a case study. J Autism Dev Disord 1990;20(4):479-97.

509. Olszyk RK. Changes in symptomatology and functioning of preschoolers with autism in the context of the DIR model. Dissert Abst Int 2005;66(2B):1180.

510. Orr TJ, Myles BS. The impact of rhythmic entertainment on a person with autism. Focus Autism Other Dev Disabil 1998;13(3):163.

511. Owen-Deschryver JS. Promoting social interactions between students with autism and their peers in inclusive school settings [dissertation]. Stony Brook (NY): State University of New York; 2003.

512. Paisey TJ, Fox S, Curran C, et al. Case study: reinforcement control of severe aggression exhibited by a child with autism in a family home. Behav Resid Treat 1991;6(4):289-302.

513. Paleo S. Preschool treatment of autism spectrum disorders: analysis of a combined approach. Dissert Abst Int B: Sciences and Engineering 2005;65(9-B):4845.

514. Panerai S, Ferrante L, Caputo V. The TEACCH strategy in mentally retarded children with autism: a multidimensional assessment: pilot study. J Autism Dev Disord 1997;27(3):345-7.

515. Panerai S, Ferrante L, Caputo V, et al. Use of structured teaching for treatment of children with autism and severe and profound mental retardation. Educ Training Ment Retard Dev Disabil 1998;33(4):367-74.

516. Parker JL. A photo-electric pen for producing action feedback to aid development in handicapped children of fine visual-motor skills: tracing and writing. Except Children 1975;22(1):13-22.

517. Parsons L. Using video to teach social skills to secondary students with autism. Teach Except Child 2006;39 (2):32-8.

518. Pasiali V. The use of prescriptive therapeutic songs in a home-based environment to promote social skills acquisition by children with autism: three case studies. Music Ther Perspect 2004;22(1):11-20.

519. Patel MR, Carr JE, Dozier CL. On the role of stimulus preference assessment in the evaluation of contingent access to stimuli associated with stereotypy during behavioral acquisition. Behav Intervent 1998;13(4):269-74.

520. Pelios LV. Increasing workskills in children with autism via delayed consequences and fading supervision [dissertation]. Philadelphia (PA): Temple University; 2000.

521. Pelios LV, MacDuff GS, Axelrod S. The effects of a treatment package in establishing independent academic work skills in children with autism. Educ Treat Child 2003;26(1):1-21.

522. Perry R, Cohen I, DeCarlo R. Case study: deterioration, autism, and recovery in two siblings. J Am Acad Child Adolesc Psychiatry 1995;34(2):232-7.

523. Persson B. Brief report: a longitudinal study of quality of life and independence among adult men with autism. J Autism Dev Disord 2000;30(1):61-6.

524. Peterson SL, Bondy AS, Vincent Y, et al. Effects of altering communicative input for students with autism and no speech: two case studies. AAC: Augment Altern Commun 1995;11(2):93-100.

525. Peterson SMP, Caniglia C, Royster AJ. Application of choice-making intervention for a student with multiply maintained problem behavior. Focus Autism Other Dev Disabil 2001;16(4):240-6.

526. Peyton RT, Lindauer SE, Richman DM. The effects of directive and nondirective prompts on noncompliant vocal behavior exhibited by a child with autism. J Appl Behav Anal 2005;38(2):251-5.

527. Piazza CC. Differential reinforcement of alternative behavior and demand fading in the treatment of escape-maintained destructive behavior. J Appl Behav Anal 1996;29(4):569-72.

528. Piazza CC, Fisher WW, Hanley GP, et al. Treatment of pica through multiple analyses of its reinforcing functions. J Appl Behav Anal 1998;31(2):165-89.

529. Pierce K, Schreibman L. Increasing complex social behaviors in children with autism: effects of peer-implemented pivotal response training. J Appl Behav Anal 1995;28(3):285-95.

530. Pierce K, Schreibman L. Multiple peer use of pivotal response training to increase social behaviors of classmates with autism: results from trained and untrained peers. J Appl Behav Anal 1997;30(1):157-60.

531. Pierce K, Schreibman L. Using peer trainers to promote social behavior in autism: are they effective at enhancing multiple social modalities? Focus Autism Other Dev Disabil 1997;12(4):207-18.

532. Pierce KL. The assessment and treatment of social behavior in autism: towards a naturalistic approach [dissertation]. San Diego (CA): University of California; 1996.

533. Pierce KL, Schreibman L. Teaching daily living skills to children with autism in unsupervised settings through pictorial self-management. J Appl Behav Anal 1994;27(3):471-81.

534. Pilebro C, Backman B. Teaching oral hygiene to children with autism. Int J Paediatr Dent 2005;15(1):1-9.

535. Preator KK. Overcorrection and alternative response training in the reduction of an autistic child's inappropriate touching. School Psychol Rev 1984;13(1):107-10.

536. Preis J. The effect of picture communication symbols on the verbal comprehension of commands by young children with autism. Focus Autism Other Dev Disabil 2006;21(4):194-210.

537. Preis J. The effect of picture communication symbols on the verbal comprehension of young children with autism [dissertation]. Baltimore (MD): Johns Hopkins University; 2002.

538. Probst P, Hillig S. Computer-assisted training of purchasing skills: a single case study of a 15-year-old boy with early infantile autism [German]. Kindheit und Entwicklung 2004;13(2):122-8.

539. Pushkarenko K. Enhancing the structure of a swimming program for three boys with autism through the use of activity schedules [dissertation]. Montreal (QC): Mcgill University (Canada); 2004.

540. Randall PE, Gibb C. Structured management and autism. Br J Special Educ 1987;14(2):68-70.

541. Rapp JT, Dozier CL, Carr JE. Functional assessment and treatment of pica: a single-case experiment. Behav Intervent 2001;16(2):111-25.

542. Rapp JT, Dozier CL, Carr JE, et al. Functional analysis of hair manipulation: a replication and extension. Behav Intervent 2000;15(2):121-33.

543. Rapp JT, Vollmer TR, Hovanetz AN. Evaluation and treatment of swimming pool avoidance exhibited by an adolescent girl with autism. Behav Ther 2005;36(1):101-5.

544. Ratusnik CM, Ratusnik DL. A comprehensive communication approach for a ten-year-old nonverbal autistic child. Am J Orthopsychiat 1974;44(3):396-403.

545. Ray KP, Skinner CH, Watson TS. Transferring stimulus control via momentum to increase compliance in a student with autism: a demonstration of collaborative consultation. School Psychol Rev 1999;28(4):622-8.

546. Ray TC, King LJ, Grandin T. The effectiveness of self-initiated vestibular stimulation in producing speech sounds in an autistic child. Occup Ther J Res 1988;8(3):186-90.

547. Reaven J, Hepburn S. Cognitive-behavioral treatment of obsessive-compulsive disorder in a child with Asperger syndrome: a case report. Autism 2003;7(2):145-64.

548. Redinius PL. The effect of a digitized video schedule on task initiation for people with autism. Dissert Abst Int A: Humanities and Social Sciences 2004;64(8-A):2780.

549. Rees RJ. Key variables determining the acquisition of speech by autistic five year olds. Except Children 1975;22(3):159-72.

550. Reese RM, Sherman JA, Sheldon JB. Reducing disruptive behavior of a group-home resident with autism and mental retardation. J Autism Dev Disord 1998;28(2):159-65.

551. Reeve SA. Effects of modeling, video modeling, prompting, and reinforcement strategies on increasing helping behavior in children with autism. Dissert Abst Int B: Sciences and Engineering 2001;62(3-B):1561.

552. Reichle J, Barrett C, Tetlie RR, et al. The effect of prior intervention to establish generalized requesting on the acquisition of object labels. AAC: Augment Altern Commun 1987;3(1):3-11.

553. Reichle J, McComas J, Dahl N, et al. Teaching an individual with severe intellectual delay to request assistance conditionally. Educ Psychol 2005;25(2-3):275-86.

554. Reid G, Collier D, Cauchon M. Skill acquisition by children with autism: influence of prompts. Adapt Phys Act Q 1991;8(4):357-66.

555. Reijonen J. Measuring changes in spontaneous play behavior in preschoolers with autism associated with a receptive language intervention. Dissert Abst Int A: Humanities and Social Sciences 1997;57(7-A):2861.

556. Reinecke DR. Increasing the number of play activities chosen by children with autism: effects of exposure and response-independent reinforcers. Dissert Abst Int B: Sciences and Engineering 2005;65(12-B):6638.

557. Reinecke DR, Newman B, Meinberg DL. Self-management of sharing in three pre-schoolers with autism. Educ Training Ment Retard Dev Disabil 1999;34(3):312-7.

558. Reinhartsen DB, Garfinkle AN, Wolery M. Engagement with toys in two-year-old children with autism: teacher selection versus child choice. Res Pract Pers Sev Disabil 2002;27(3):175-87.

559. Reitman MR. Effectiveness of music therapy interventions on joint attention in children diagnosed with autism: a pilot study [dissertation]. Miami (FL): Carlos Albizu University; 2005.

560. Remington B, Clarke S. Acquisition of expressive signing by autistic children: an evaluation of the relative effects of simultaneous communication and sign-alone training. J Appl Behav Anal 1983;16(3):315-27.

561. Ricciardi JN, Luiselli JK. Behavioral intervention to eliminate socially mediated urinary incontinence in a child with autism. Child Fam Behav Ther 2003;25(4):53-63.

562. Rigsby-Eldredge M, McLaughlin TF. The effects of modeling and praise on self-initiated behavior across settings with two adolescent students with autism. J Dev Phys Disabil 1992;4(3):205-18.

563. Rincover A, Koegel RL. Setting generality and stimulus control in autistic children. J Appl Behav Anal 1975;8(3):235-46.

564. Rocha ML, Schreibman L, Stahmer AC. Effectiveness of training parents to teach joint attention in children with autism. J Early Interv 2007;29(2):154-72.

565. Roeyers H. A peer-mediated proximity intervention to facilitate the social interactions of children with a pervasive developmental disorder. Br J Special Educ 1995;22(4):161-4.

566. Rogers MF, Myles BS. Using social stories and comic strip conversations to interpret social situations for an adolescent with Asperger syndrome. Interv Sch Clin 2001;36(5):310-3.

567. Rogers SJ, DiLalla DL. A comparative study of the effects of a developmentally based instructional model on young children with autism and young children with other disorders of behavior and development. Top Early Child Spec 1991;11(2):29-47.

568. Rogers SJ, Hayden D, Hepburn S, et al. Teaching young nonverbal children with autism useful speech: a pilot study of the Denver Model and PROMPT interventions. J Autism Dev Dis 2006;36(8):1007-24.

569. Rogers SJ, Lewis H. An effective day treatment model for young children with pervasive developmental disorders. J Am Acad Child Adolesc Psychiatry 1989;28(2):207-14.

570. Rolland C, Nanclares-Nogues V. Parent-therapist implemented, integrated treatment for young children with autism: a comparison with therapist-implemented. J Dev Behav Pediatr 2005;26(6):464.

571. Ross B. Effects of verbal cues on the eye gaze behaviors of children with autism during turn-taking activities. Dissert Abst Int B: Sciences and Engineering 2004;(64):B6049-50.

572. Ross DE. Generalized imitation and the mand: inducing first instances of vocal verbal behavior in young children with autism [dissertation]. New York (NY): Columbia University; 1998.

573. Ross DE. Replacing faculty conversational exchanges for children with autism by establishing a functionally equivalent alternative response. Educ Training Ment Retard Dev Disabil 2002;37(4):343-62.

574. Ross DE, Greer RD. Generalized imitation and the mand: inducing first instances of speech in young children with autism. Res Dev Disabil 2003;24(1):58-74.

575. Roth SR. A comparison of graduated guidance and a system of least prompts when teaching children with autism in a discrete trial format [dissertation]. Stockton (CA): University of the Pacific; 1999.

576. Rotholz DA, Berkowitz SF, Burberry J. Functionality of two modes of communication in the community by students with developmental disabilities: a comparison of signing and communication books. J Assoc Pers Severe Hand 1989;14(3):227-33.

577. Rotholz DA, Luce SC. Alternative reinforcement strategies for the reduction of self-stimulatory behavior in autistic youth. Educ Treat Child 1983;6(4):363-77.

578. Russo DC, Koegel RL. A method for integrating an autistic child into a normal public-school classroom. J Appl Behav Anal 1977;10(4):579-90.

579. Russo LA. The effects of a tactile prompting device on the requesting behavior of a child with autism [dissertation]. Denton (TX): University of North Texas; 1999.

580. Rydell PJ. Social-communicative control and its effect on echolalia in children with autism. Dissert Abst Int 1990;50(9-B):3949.

581. Sainato DM, Goldstein H, Strain PS. Effects of self-evaluation on preschool children's use of social interaction strategies with their classmates with autism. J Appl Behav Anal 1992;25(1):127-41 .

582. Salazar Smith A. Increasing social initiations in preschoolers with autism using a combination of social stories, pictorial cues and role play. Dissert Abst Int A: Humanities and Social Sciences 2005;65(7-A):2492.

583. Salmon MD. Script training with storybooks and puppets: a social skills intervention package across settings for young children with autism and their typically developing peers [dissertation]. Columbus (OH): Ohio State University; 2005.

584. Saloviita T. Dry bed training method in the elimination of bed-wetting in two adults with autism and severe mental retardation. Cogn Behav Ther 2002;31(3):135-40.

585. Salvin A, Routh DK, Foster RE Jr, et al. Acquisition of modified American Sign Language by a mute autistic child. J Autism Child Schiz 1977;7(4):359-71.

586. Sampson CE. The buddy skills program: helping children with autism improve their social skills [dissertation]. Halifax (NS): Mount Saint Vincent University; 2003.

587. Sanchez A. The use of picture activity schedules to promote independence and reduce challenging behavior [dissertation]. Fullerton (CA): California State University; 2004.

588. Sansosti FJ. Using video modeled social stories to increase the social communication skills of children with high functioning autism/Asperger's syndrome [dissertation]. Tampa (FL): University of South Florida; 2005.

589. Sansosti FJ, Powell-Smith KA. Using social stories to improve the social behavior of children with Asperger syndrome. J Pos Behav Interv 2006;8(1):43-57.

590. Santarcangelo S, Dyer K, Luce SC. Generalized reduction of disruptive behavior in unsupervised settings through specific toy training. J Assoc Pers Severe Hand 1987;12(1):38-44.

591. Saperston B. The use of music in establishing communication with an autistic mentally retarded child. J Music Ther 1973;10(4):184-8.

592. Sarokoff RA, Taylor BA, Poulson CL. Teaching children with autism to engage in conversational exchanges: script fading with embedded textual stimuli. J Appl Behav Anal 2001;34(1):81-4.

593. Sasso GM. The use of multiple procedures in the integration of students with severe handicapping conditions. 10th Annual conference of the association for the severely handicapped; San Francisco, CA. 1983.

594. Saunders RR, Saunders MD, Brewer A, et al. Reduction of self injury in two adolescents with profound retardation by the establishment of a supported routine. Behav Intervent 1996;11(2):59-86.

595. Sawyer LM, Luiselli JK., Ricciardi JN, et al. Teaching a child with autism to share among peers in an integrated preschool classroom: acquisition, maintenance, and social validation. Educ Treat Child 2005;28(1):1-10.

596. Scattone D. Increasing appropriate social interactions of children with autistic spectrum disorders using social stories. Dissert Abst Int B: Sciences and Engineering 2003;63(10-B):4885.

597. Scattone D, Wilczynski SM, Edwards RP, et al. Decreasing disruptive behaviors of children with autism using social stories. J Autism Dev Disord 2002;32(6):535-43.

598. Schairer KS, Nelson NW. Communicative possibilities of written conversations with adolescents who have autism. Child Lang Teach Ther 1996;12(2):164-80.

599. Schepis JA. The effects of a fluency building intervention on the language of preschool children with autism and developmental disabilities and their peers. Dissert Abst Int A: Humanities and Social Sciences 2003;63(7-A):2506.

600. Schepis MM, Reid DH, Behrmann MM, et al. Increasing communicative interactions of young children with autism using a voice output communication aid and naturalistic teaching. J Appl Behav Anal 1998;31(4):561-78.

601. Schepis MM, Reid DH, Fitzgerald JR, et al. A program for increasing manual signing by autistic and profoundly retarded youth within the daily environment. J Appl Behav Anal 1982;15(3):363-79.

602. Scherer NJ, Olswang LB. Using structured discourse as a language intervention technique with autistic children. J Speech Hear Disord 1989;54(3):383-94.

603. Schindler HR, Horner RH. Generalized reduction of problem behavior of young children with autism: building trans-situational interventions. Am J Ment Retard 2005;110(1):36-47.

604. Schleien SJ, Heyne LA, Berken SB. Integrating physical education to teach appropriate play skills to learners with autism: a pilot study. Adapt Phys Act Q 1988;5(3):182-92.

605. Schleien SJ, Mustonen T, Rynders JE. Participation of children with autism and nondisabled peers in a cooperatively structured community art program. J Autism Dev Disord 1995;25(4):397-413.

606. Schleien SJ, Rynders JE, Mustonen T, et al. Effects of social play activities on the play behavior of children with autism. J Leisure Res 1990;22(4):317-28.

607. Schlosser RW, Blischak DM. Effects of speech and print feedback on spelling by children with autism. J Speech Lang Hear Res 2004;47(4):848.

608. Schlosser RW, Blischak DM, Belfiore PJ, et al. Effects of synthetic speech output and orthographic feedback on spelling in a student with autism: a preliminary study. J Autism Dev Disord 1998;28(4):309-19.

609. Schmit J, Alper S, Raschke D, et al. Effects of using a photographic cueing package during routine school transitions with a child who has autism. Ment Retard 2000;38(2):131-7.

610. Schneiderman MK, Leccese LR. I/you acquisition and reciprocation. Lang Speech Hear Serv Sch 1982;13(2):116-20.

611. Schneiter R, Devine MA. Reduction of self-injurious behaviors of an individual with autism: use of a leisure communication book. Ther Recreation J 2001;35(3):207-19.

612. Schreibman L. Effects of within-stimulus and extra-stimulus prompting on discrimination learning in autistic children. J Appl Behav Anal 1975;8(1):91-112.

613. Schreibman L, Charlop MH. S + versus S- fading in prompting procedures with autistic children. J Exp Child Psychol 1981;31(3):508-20.

614. Schreibman L, Charlop MH, Koegel RL. Teaching autistic children to use extra-stimulus prompts. J Exp Child Psychol 1982;33(3):475-91.

615. Schreibman L, Whalen C, Stahmer AC. The use of video priming to reduce disruptive transition behavior in children with autism. J Pos Behav Interv 2000;2(1):3-11.

616. Schuler AL. Beyond echoplaylia: promoting language in children with autism. Autism 2003;7(4):455-69.

617. Schwartz IS, Sandall SR, McBride BJ, et al. Project DATA (Developmentally Appropriate Treatment for Autism): an inclusive school-based approach to educating young children with autism. Top Early Child Spec 2004;24(3):156-68.

618. Secan KE, Egel AL, Tilley CS. Acquisition, generalization, and maintenance of question-answering skills in autistic children. J Appl Behav Anal 1989;22(2):181-96.

619. Seung HK, Ashwell S, Elder JH, et al. Verbal communication outcomes in children with autism after in-home father training. J Intell Disabil Res 2006;50(2):139-50.

620. Shabani DB. Increasing verbal initiations in children with autism: effects of a tactile prompt [dissertation]. Stockton (CA): University of the Pacific; 2001.

621. Shabani DB, Katz RC, Wilder DA, et al. Increasing social initiations in children with autism: effects of a tactile prompt. J Appl Behav Anal 2002;35(1):79-83.

622. Shabani DB, Wilder DA, Flood WA. Reducing stereotypic behavior through discrimination training, differential reinforcement of other behavior, and self monitoring. Behav Intervent 2001;16(4):279-86 .

623. Shafer MS, Egel AL, Neef NA. Training mildly handicapped peers to facilitate changes in the social interaction skills of autistic children. J Appl Behav Anal 1984;17(4):461-76.

624. Shaw S. Behavioral treatment for children with autism: a comparison between discrete trial training and pivotal response training in teaching emotional perspective-taking skills [dissertation]. Dissert Abst Int 2001;61(11B):6121.

625. Shearer DD. Promoting independent interactions between preschoolers with autism and their nondisabled peers: an analysis of self-monitoring. Early Educ Dev 1996;7(3):205-20.

626. Sherer M, Pierce KL, Paredes S, et al. Enhancing conversation skills in children with autism via video technology: which is better, "self" or "other" as a model? Behav Modif 2001;25(1):140-58.

627. Shields-Wolfe J, Gallagher PA. Functional utilization of splinter skills for the employment of a young adult with autism. Focus Autistic Behav 1992;7(4):1-16.

628. Shigley RH, Shigley JK. Imitative speech training of an autistic child in the home. West Carolina Univ J Educ 1975;7(1):38-42.

629. Shipley-Benamou R, Lutzker JR, Taubman M. Teaching daily living skills to children with autism through instructional video modeling. J Pos Behav Interv 2002;4(3):165-75, 188.

630. Shivarathnamma N. Treatment report of an autistic child: monozygotic twin concordant to autism. Indian J Clin Psychol 1979;6(2):95-100.

631. Shively JM. The effects of a remote control tactile feedback system on conversation skills in children with autism [dissertation]. Denton (TX): University of North Texas; 2003.

632. Shook SL. Teaching children with autism to ask questions in integrated preschool settings: a comparison of constant and progressive time delay. Dissert Abst Int A: Humanities and Social Sciences 2000;60(8-A):2871.

633. Short AB. Short-term treatment outcome using parents as co-therapists for their own autistic children. J Child Psychol Psyc 1984;25(3):443-58.

634. Sidener TM, Carr JE, Firth AM. Superimposition and withholding of edible consequences as treatment for automatically reinforced stereotypy. J Appl Behav Anal 2005;38(1):121-4.

635. Sigafoos J, Littlewood R. Communication intervention on the playground: a case study on teaching requesting to a young child with autism . Int J Disability Dev Educ 1999;46(3):421-9.

636. Sigafoos J, Meikle B. Functional communication training for the treatment of multiply determined challenging behavior in two boys with autism. Behav Modif 1996;20(1):60-84.

637. Sigafoos J, O'Reilly M, Seely-York S, et al. Teaching students with developmental disabilities to locate their AAC device. Res Dev Disabil 2004;25(4):371-83 .

638. Sigafoos J, O'Reilly MF, Seely-York S, et al. Transferring AAC intervention to the home. Disabil Rehabil 2004;26(21-22):1330-4.

639. Sigafoos J, Saggers E. A discrete-trial approach to the functional analysis of aggressive behaviour in two boys with autism. Aust NZ J Dev Disab 1995;20(4):287-97.

640. Silla VA. The effects of prompting with visual and textual cues in narrative comprehension of high-functioning children with autism [dissertation]. Indiana (PA): Indiana University of Pennsylvania; 2004.

641. Silva LMT, Cignolini A. A medical Qigong methodology for early intervention in autism spectrum disorder: a case series. Am J Chin Med 2005;33(2):315-27 .

642. Silver EB. Operant conditioning of speech sounds in an autistic child. S Afr JPsychol 1970;3-12.

643. Simpson A, Langone J, Ayres KM. Embedded video and computer based instruction to improve social skills for students with autism. Educ Training Dev Disabil 2004;39(3):240-52.

644. Simpson RL, Myles BS. Effectiveness of facilitated communication with children and youth with autism. J Spec Educ 1995;28(4):424-39.

645. Singh NN. Reprogramming the social environment of an autistic child. New Zeal Med J 1978;87(606):135-8.

646. Smith AE, Camarata S. Using teacher-implemented instruction to increase language intelligibility of children with autism. J Pos Behav Interv 1999;1(3):141-51.

647. Smith DE, Olson M, Barger F, et al. The effects of improved auditory feedback on the verbalizations of an autistic child. J Autism Dev Disord 1981;11(4):449-54.

648. Smith MD. Managing the aggressive and self-injurious behavior of adults disabled by autism. J Assoc Pers Severe Hand 1985;10(4):228-32.

649. Smith MD, Coleman D. Managing the behavior of adults with autism in the job setting. J Autism Dev Disord 1986;16(2):145-54.

650. Smith MD, Haas PJ, Belcher RG. Facilitated communication: the effects of facilitator knowledge and level of assistance on output. J Autism Dev Disord 1994;24(3):357-67.

651. Smith MR, Lerman DC. A preliminary comparison of guided compliance and high-probability instructional sequences as treatment for noncompliance in children with developmental disabilities. Res Dev Disabil 1999;20(3):183-95.

652. Smith T. Improving memory to promote maintenance of treatment gains in children with autism. Psychol Rec 1994;44(4):459-73.

653. Smith T, Buch GA, Gamby TE. Parent-directed, intensive early intervention for children with pervasive developmental disorder. Res Dev Disabil 2000;21(4):297-309.

654. Soenksen D, Alper S. Teaching a young child to appropriately gain attention of peers using a social story intervention. Focus Autism Other Dev Disabil 2006;21(1):36-44.

655. Solnick JV, Rincover A, Peterson CR. Some determinants of the reinforcing and punishing effects of timeout. J Appl Behav Anal 1977;10(3):415-24.

656. Solomon R, Necheles J, Ferch C, et al. Pilot study of a parent training program for young children with autism: The PLAY Project Home Consultation program. Autism 2007;11(3):205-24.

657. Solomons S. Using aromatherapy massage to increase shared attention behaviours in children with autistic spectrum disorders and severe learning difficulties. Br J Special Educ 2005;32(3):127-37 .

658. Son S. Comparing two modes of AAC intervention for children with autism [dissertation]. Austin (TX): University of Texas; 2005.

659. Sonnenmeier RM, McSheehan M, Jorgensen CM. A case study of team supports for a student with autism's communication and engagement within the general education curriculum: preliminary report of the beyond access model. AAC: Augment Altern Commun 2005;21(2):101-15.

660. Southern TA. The effectiveness of self-management strategies, designed to target interpretation and understanding of the classroom environment and student responsibilities and requirements, among students (aged 5-8) with autism spectrum disorder in inclusive education classrooms [dissertation]. Pocatello (IH): Idaho State University; 2004.

661. Spence-Cochran KG. An investigation of the proficiency level of high school students with autism and mental retardation within community-based job settings: the relationship between the use of a hand-held computer compared to staff modeling for accurate novel job skill acquisition and student learning [dissertation]. Orlando (FL): University of Central Florida; 2004.

662. Spencer LG. Comparing the effectiveness of static pictures vs. video modeling on teaching requesting skills to elementary children with autism [dissertation]. Atlanta (GA): Georgia State University; 2002.

663. Spillane MM. The effect of instructional method on symbol acquisition by students with severe disabilities [dissertation]. Lincoln (NE): The University of Nebraska; 1999.

664. Stafford N. Can emotions be taught to a low functioning autistic child? Early Child Dev Care 2000;164:105-26.

665. Stahmer AC. Teaching symbolic play skills to children with autism using pivotal response training. J Autism Dev Disord 1995;25(2):123-41.

666. Stahmer AC, Ingersoll B, Koegel RL. Inclusive programming for toddlers autism spectrum disorders: outcomes from the Children's Toddler School. J Pos Behav Interv 2004;6(2):67-82.

667. Stahmer AC, Schreibman L. Teaching children with autism appropriate play in unsupervised environments using a self-management treatment package. J Appl Behav Anal 1992;25(2): 447-59.

668. Starr E, Zenker K. Understanding autism in the context of music therapy: bridging theory and practice. Can J Music Ther 1998;6(1):1-19.

669. Steinfeld BI. Teaching an autistic child receptive labels: a comparison of two types of extra-stimulus prompting procedures. 1979;16.

670. Stevens-Long J, Schwarz JL, Bliss D. The acquisition and generalization of compound sentence structure in an autistic child. Behav Ther 1976;7(3):397-404.

671. Stevenson CL, Krantz PJ, Mcclannahan LE. Social interaction skills for children with autism: a script-fading procedure for nonreaders. Behav Intervent 2000;15(1):1-20.

672. Stiebel D. Promoting augmentive communication during daily routines: a parent problem-solving intervention. J Pos Behav Interv 1999;1(3):159-69.

673. Stoelb M. Teaching expressive labeling to children with autism via videotape modeling. Dissert Abst Int A: Humanities and Social Sciences 2004;65(6-A):2162.

674. Strain PS. Activity engagement and social interaction development in young children with autism: an examination of "free" intervention effects. J Emot Behav Disord 1995;3(2):108-23.

675. Strain PS. Generalization of autistic children's social behavior change: effects of developmentally integrated and segregated settings. Anal Interv Dev Disabil 1983;3(1):23-34.

676. Strain PS, Hoyson M. The need for longitudinal, intensive social skill intervention: LEAP follow-up outcomes for children with autism. Top Early Child Spec 2000;20(2):116-22.

677. Strain PS, Kerr MM, Ragland EU. Effects of peer-mediated social initiations and prompting/reinforcement procedures on the social behavior of autistic children. J Autism Dev Disord 1979;9(1):41-54.

678. Strain PS, Kohler FW, Storey K, et al. Teaching preschoolers with autism to self-monitor their social interactions: an analysis of results in home and school settings. J Emot Behav Disord 1994;2(2):78-88.

679. Stuecher U. Tommy: a treatment study of an autistic child. Reston (VA): Council for Exceptional Children, 1972.

680. Sugai G, White WJ. Effects of using object self-stimulation as a reinforcer on the prevocational work rates of an autistic child. J Autism Dev Disord 1986;16(4):459-71.

681. Sullivan CL. The effects of sibling-implemented training on social behaviors of autistic children. Dissert Abst Int B: Sciences and Engineering 1999;60(6-B):2964.

682. Sulzbacher SI, Costello JM. A behavioral strategy for language training of a child with autistic behaviors. J Speech Hear Disord 1970;35(3):256-76.

683. Summers JA, Craik FIM. The effects of subject-performed tasks on the memory performance of verbal autistic children. J Autism Dev Disord 1994;24(6):773-90 .

684. Sundberg ML, Endicott K, Eigenheer P. Using intraverbal prompts to establish tacts for children with autism. Anal Verbal Behav 2000;17 2000:89-104.

685. Swaggart B, Gagnon E, Bock SJ, et al. Using social stories to teach social and behavioral skills to children with autism. Focus Autistic Behav 1995;10(1):1-16.

686. Swaine JM. Teaching language skills to children with autism through the use of social stories [dissertation]. Halifax (NS): Dalhousie University; 2004 .

687. Symon JB. Expanding interventions for children with autism: parents as trainers. J Pos Behav Interv 2005;7(3):159-73.

688. Symon JB. Parent education programs for families of children with autism living in geographically distant locations: generalized spread of effects [dissertation]. Santa Barbara (CA): University of California; 2002.

689. Symons FJ, MacLean WE Jr. Analyzing and treating severe behavior problems in people with developmental disabilities: observational methods using computer-assisted technology. Thompson T, Felce D, Symons FJ, editors. Behavioral observation: technology and applications in developmental disabilities. Baltimore: Brookes Publishing; 2000. p. 143-57.

690. Taber TA, Seltzer A, Heflin LJ, et al. Use of self-operated auditory prompts to decrease off-task behavior for a student with autism and moderate mental retardation. Focus Autism Other Dev Disabil 1999;14(3):159-66.

691. Takeshima K. Pyramidal staff training: teaching play and manding to children with autism and developmental disabilities [dissertation]. Kalamazoo (MI): Western Michigan University; 2005.

692. Takeuchi K, Yamamoto J. A case study of examining the effects of self-monitoring on improving academic performance by a student with autism. Jpn J Special Educ 2001;38(6):105-16.

693. Takezawa T. Peer-mediated social interaction intervention for preschool children with autism (Japan). Dissert Abst Int A: Humanities and Social Sciences 2005;65(8-A):2952.

694. Tanner BA, Zeiler M. Punishment of self-injurious behavior using aromatic ammonia as the aversive stimulus. J Appl Behav Anal 1975;8(1):53-7.

695. Taras ME, Matson JL, Leary C. Training social interpersonal skills in two autistic children. J Behav Ther Exp Psy 1988;19(4):275-80.

696. Tarbox J, Wallace MD, Tarbox RSF. Successful generalized parent training and failed schedule thinning of response blocking for automatically maintained object mouthing . Behav Intervent 2002;17(3):169-78.

697. Taylor BA, Hoch H, Weissman M. The analysis and treatment of vocal stereotypy in a child with autism. Behav Intervent 2005;20(4):239-53.

698. Taylor BA, Hughes CE, Richard E, et al. Teaching teenagers with autism to seek assistance when lost. J Appl Behav Anal 2004;37(1):79-82.

699. Taylor BA, Levin L. Teaching a student with autism to make verbal initiations: effects of a tactile prompt. J Appl Behav Anal 1998;31(4):651-4.

700. Taylor BA, Levin L, Jasper S. Increasing play-related statements in children with autism toward their siblings: effects of video modeling. J Dev Phys Disabil 1999;11(3):253-64.

701. Thiemann KS. Effects of peer training and visually-cued interventions on social-communication of children with pervasive developmental disorders in integrated elementary classrooms. Dissert Abst Int B: Sciences and Engineering 2001;(61):5270B-1.

702. Thiemann KS, Goldstein H. Effects of peer training and written text cueing on social communication of school-age children with pervasive developmental disorder. J Speech Lang Hear Res 2004;47(1):126-44.

703. Thomas N, Smith C. Developing play skills in children with autistic spectrum disorders. Educ Psychol Pract 2004;20(3):195-206.

704. Thompson AR, Beail N. The treatment of auto-erotic asphyxiation in a man with severe intellectual disabilities: the effectiveness of a behavioural and educational programme. J Appl Res Intellect 2002;15(1):36-47.

705. Thompson RH, Fisher WW, Piazza CC, et al. The evaluation and treatment of aggression maintained by attention and automatic reinforcement. J Appl Behav Anal 1998;31(1):103-16.

706. Thorp DM, Stahmer AC, Schreibman L. Effects of sociodramatic play training on children with autism. J Autism Dev Disord 1995;25(3):265-82.

707. Thorwarth CA. Generalization of parent training effects to structured versus unstructured settings: the use of discreet commands and reinforcements by parents of autistic children who participated in a parent training program [dissertation]. New Brunswick (NJ): Rutgers the State University of New Jersey; 1982 .

708. Tiegerman E, Primavera L. Object manipulation: an interactional strategy with autistic children. J Autism Dev Disord 1981;11(4):427-38.

709. Tiegerman E, Primavera LH. Imitating the autistic child: facilitating communicative gaze behavior. J Autism Dev Disord 1984;14(1):27-38.

710. Tincani M. Comparing the picture exchange communication system and sign language training for children with autism. Focus Autism Other Dev Disabil 2004;19(3):152-63.

711. Tincani MJ. Effects of selection-based versus topography-based communication training on the acquisition of mands by children with autism and multiple disabilities. Dissert Abst Int A: Humanities and Social Sciences 2003;63(7-A):2506.

712. Tincani MJ, Castrogiavanni A, Axelrod S. A comparison of the effectiveness of brief versus traditional functional analyses. Res Dev Disabil 1999;20(5):327-38.

713. Tjus T, Heimann M, Nelson K. Reading acquisition by implementing a multimedia intervention strategy for fifty children with autism or other learning and communication disabilities. J Cog Behav Psychother 2004;4(2):203-21.

714. Tjus T, Heimann M, Nelson KE. Gains in literacy through the use of a specially developed multimedia computer strategy. Autism 1998;2(2):139-56.

715. Tjus T, Heimann M, Nelson KE. Interaction patterns between children and their teachers when using a specific multimedia and communication strategy: observations from children with autism and mixed intellectual disabilities. Autism 2001;5(2):175-87.

716. Tomporowski PD. Training an autistic client: the effect of brief restraint on disruptive behavior. J Behav Ther Exp Psy 1983;14(2):169-73.

717. Trautman RC, Ryley AT. Early intervention and five years later with an autistic child and her family. English, 1989.

718. Treffert DA, McAndrew JB, Dreifuerst P. An inpatient treatment program and outcome for 57 autistic and schizophrenic children. J Autism Child Schiz 1973;3(2):138-53.

719. Treiber DM. Increasing the use of comments by a child with an autistic spectrum disorder and hyperlexia: a case study. Dissert Abst Int B: Sciences and Engineering Jan;(60):3245B.

720. Trimarchi CL. The implementation and evaluation of a social skills training program for children with Asperger syndrome. Dissert Abst Int B: Sciences and Engineering 2004;65(5-B):2655.

721. Tsao L. The effectiveness of sibling-mediated social intervention for children with autism [dissertation]. Bloomington (IN): Indiana University; 2004.

722. Tu JC. The role of joint control in the manded selection responses of non-vocal children with autism. Dissert Abst Int A: Humanities and Social Sciences 2005;65(12-A):4465.

723. Tustin RD. The effects of advance notice of activity transitions on stereotypic behavior. J Appl Behav Anal 1995;28(1):91-2.

724. Vazquez CA. Brief report: a multitask controlled evaluation of facilitated communication. J Autism Dev Disord 1994;24(3):369-79.

725. Vener SM. Analyzing the effects of self recording on teacher use of incidental teaching procedures to teach language to children with autism. Dissert Abst Int 2002;(62):4062A.

726. Vismara LA. Understanding the role of motivation in joint attention behaviors for children with autism [dissertation]. Santa Barbara (CA): University of California; 2005.

727. Volkert VM, Lerman DC, Vorndran C. The effects of reinforcement magnitude on functional analysis outcomes. J Appl Behav Anal 2005;38(2):147.

728. von Stosch T. Chances and limits of holding therapy with autistic children in day treatment: a case study. Fruhforderung interdisziplinar 1986;5(3):126-31.

729. Vorpahl CM. Use of fading procedures and positive reinforcement to increase consumption of non-preferred food in a child with autism [dissertation]. Denton (TX): University of North Texas; 2004.

730. Wager KM. The effects of music therapy upon an adult male with autism and mental retardation: a four-year case study. Music Therapy 2000;18(2):131-40.

731. Walker GR, Hinerman PS, Jenson WR, et al. Sign language as a prompt to teach a verbal "yes" and "no" discrimination to an autistic boy. Child Behav Ther 1981;3(4):77-86.

732. Ward AJ, Leith VM. The joint treatment of an "autistic" child by clinical psychology and speech therapy. Int J Child Psychother 1973;2(4):451-70.

733. Watanabe M, Sturmey P. The effect of choice-making opportunities during activity schedules on task engagement of adults with autism. J Autism Dev Disord 2003;33(5):535-8.

734. Watters RG, Wheeler LJ, Watters WE. The relative efficiency of two orders for training autistic children in the expressive and receptive use of manual signs. J Commun Disord 1981;14(4):273-85.

735. Webb BJ. Effects of social skill training for high-functioning adolescents with autism spectrum disorder. Dissert Abst Int A: Humanities and Social Sciences 2003;63(10-A):3519.

736. Webb BJ, Miller SP, Pierce TB, et al. Effects of social skill instruction for high-functioning adolescents with autism spectrum dsorders. Focus Autism Other Dev Disabil 2004;19(1):53-62.

737. Weber RC, Thorpe J. Teaching children with autism through task variation in physical education. Except Children 1992;59(1):77-86.

738. Weiskop S, Matthews J, Richdale A. Treatment of sleep problems in a 5-year-old boy with autism using behavioural principles. Autism 2001;5(2):209-21.

739. Weiskop S, Richdale A, Matthews J. Behavioural treatment to reduce sleep problems in children with autism or fragile X syndrome. Dev Med Child Neurol 2005;47(2):94-104.

740. Weiss MJ, Delmolino L. The relationship between early learning rates and treatment outcome for children with autism receiving intensive home-based applied behavior analysis. Behav Anal Today 2006;7 ( 1):96-110.

741. Weiss MJ, Green G, Mulick JA, et al. Differential rates of skill acquisition and outcomes of early intensive behavioral intervention for autism. Behav Intervent 1999;14(1):3-36.

742. Weiss MJ, Wagner SH, Bauman ML. A validated case study of facilitated communication. Ment Retard 1996;34(4):220-30 .

743. Wells KC, Forehand R, Hickey K. Effects of a verbal warning and overcorrection on stereotyped and appropriate behaviors. J Abnorm Child Psych 1977;5(4):387-403.

744. Wells KC, Forehand R, Hickey K, et al. Effects of a procedure derived from the overcorrection principle on manipulated and nonmanipulated behaviors. J Appl Behav Anal 1977;10(4): 679-87.

745. Wert BJ. Video self-modeling as a technique for increasing spontaneous requests of objects and actions in young children with autism spectrum disorders (ASD) [dissertation]. University Park (PA): Pennsylvania State University; 2002.

746. Wert BY, Neisworth JT. Effects of video self-modeling on spontaneous requesting in children with autism. J Pos Behav Interv 2003;5(1):30-4.

747. Whalen C, Schreibman L. Joint attention training for children with autism using behavior modification procedures. J Child Psychol Psyc 2003;44(3):456-68 .

748. Whalen CM. Joint attention training for children with autism and the collateral effects on language, play, imitation, and social behaviors. Dissert Abst Int B: Sciences and Engineering 2001;61(11-B):6122.

749. Whalon K. The effects of a reciprocal questioning intervention on the reading comprehension of children with autism. Dissert Abst Int A: Humanities and Social Sciences July;(66):143A.

750. Wherry JN, Edwards RP. A comparison of verbal, sign, and simultaneous systems for the acquisition of receptive language by an autistic boy. J Commun Disord 1983;16(3):201-16.

751. Whitehead JL. Treating AD-related anxiety as measured by the BASC in adolescents with asperger's disorder [dissertation]. Dissert Abst Int 2005;66(6A):2174.

752. Whittaker CA, Reynolds J. Hand signalling in dyadic proximal communication: social strengths of children with autism who do not speak. Child Lang Teach Ther 2000;16(1):43-57.

753. Wieder S, Greenspan SI. Climbing the symbolic ladder in the DIR model through floor time/interactive play. Autism 2003;7(4):425-35.

754. Wiegand PT. An activity theory-based social skills intervention for children with autism [dissertation]. Riverside (CA): University of California; 2003 .

755. Wildman RWII, Simon SJ. An indirect method for increasing the rate of social interaction in an autistic child. J Clin Psychol 1978;34(1):144-9.

756. Wilhelmsen J. The effects of vestibular stimulation and cuing on attention in an autistic child [dissertation]. New York (NY): Touro College; 1996.

757. Wilkinson LA. Supporting the inclusion of a student with asperger syndrome: a case study using conjoint behavioural consultation and self-management. Educ Psychol Pract 2005;21(4):307-26.

758. Williams JA, Koegel RL, Egel AL. Response-reinforcer relationships and improved learning in autistic children. J Appl Behav Anal 1981;14(1):53-60.

759. Williams KR. The son-rise program® intervention for autism. Autism 2006;10(1):86-102.

760. Williams TI. A social skills group for autistic children. J Autism Dev Disord 1989;19(1):143-55.

761. Wimpory D, Chadwick P, Nash S. Brief report: musical interaction therapy for children with autism: an evaluative case study with two-year follow-up. J Autism Dev Disord 1995;25(5):541-52.

762. Wishon PM, Huang A. The impact of behavior management procedures on an autistic, bi-cultural preschooler. Am Arch Rehabil Ther 1987;35(3):33-43.

763. Witte-Bakken JK. The effects of feedback on the validity of facilitated communication. Dissert Abst Int 1998;58(9B):5148.

764. Wolery M, Gast DL, Kirk K, et al. Fading extra-stimulus prompts with autistic children using time delay. Educ Treat Child 1988;11(1):29-44.

765. Wolfberg PJ, Schuler AL. Integrated play groups: a model for promoting the social and cognitive dimensions of play in children with autism. J Autism Dev Disord 1993;23(3):467-89.

766. Wong ML. Receptive learning in a child with autism: a case study [dissertation]. Boston (MA): MGH Institute of Health Professions; 2000.

767. Wong SE, Floyd J, Innocent AJ, et al. Applying a DRO schedule and compliance training to reduce aggressive and self-injurious behavior in an autistic man: a case report. J Behav Ther Exp Psy 1991;22(4):299-304 [erratum appears in J Behav Ther Exp Psychiatry 1992 Jun;23(2):147].

768. Wong SKK, Tam SF. Effectiveness of a multimedia programme and therapist-instructed training for children with autism. Int J Rehabil Res 2001; 24(4):269-78.

769. Wood MM, Swan WW. A developmental approach to educating the disturbed young child. Behav Disord 1978;3(3):197-209.

770. Woodbury PP. Students with autism: a light/sound technology intervention [dissertation]. Dissert Abst Int 1997;57(11A):4651.

771. Woodmansee KB. Sorting out the puzzle pieces of autistic disorders: examining the effectiveness of group treatment on social functioning of children with pervasive developmental disorders (PDD) [dissertation]. Dissert Abst Int 2005;65(12B):6681.

772. Woods J, Goldstein H. When the toddler takes over: changing challenging routines into conduits for communication. Focus Autism Other Dev Disabil 2003;18(3):176-81.

773. Woods TS. Programming common antecedents: a practical strategy for enhancing the generality of learning. Behav Psychother 1987;15(2): 158-80.

774. Wymbs BT, Robb JA, Chronis AM, et al. Long-term, multimodal treatment of a child with asperger's syndrome and comorbid disruptive behavior problems: a case illustration. Cogn Behav Pract 2005;12(3):338-50.

775. Yang NK, Huang TA, Schaller JL, et al. Enhancing appropriate social behaviors for children with autism in general education classrooms: an analysis of six cases. Educ Training Dev Disabil 2003;38(4):405-16.

776. Yang TR, Wolfberg PJ, Wu SC, et al. Supporting children on the autism spectrum in peer play at home and school: piloting the integrated play groups model in Taiwan. Autism 2003;7(4):437-53.

777. Young B, Simpson RL, Myles BS, et al. An examination of paraprofessional involvement in supporting inclusion of students with autism. Focus Autism Other Dev Disabil 1997;12(1):31-8.

778. Zachor DA, Eithan DB, Izhak E. Early intensive behavioral intervention for young children with autism (1.5-3 y) in a unique preschool setting-outcome after one year. Pediatr Res 2003;53(4):70A.

779. Zambolin K, Fabrizio MA, Isley S. Teaching a child with autism to answer informational questions using precision teaching. J Precis Teach Celer 2004;20(1):22-5.

780. Zanolli K, Daggett J. The effects of reinforcement rate on the spontaneous social initiations of socially withdrawn preschoolers. J Appl Behav Anal 1998;31(1):117-25.

781. Zanolli K, Daggett J, Adams T. Teaching preschool age autistic children to make spontaneous initiations to peers using priming. J Autism Dev Disord 1996;26(4):407-22.

782. Zappella M, Chiarucci P, Pinassi D, et al. Parental bonding in the treatment of autistic behavior. Ethol Sociobiol 1991;12(1):1-11.

783. Zarcone JR. Analysis of free-time contingencies as positive versus negative reinforcement. J Appl Behav Anal 1996;29(2):247-50.

784. Zelazo PR. Infant-toddler information processing treatment of children with pervasive developmental disorder and autism: part II. Infant Young Child 1997;10(2):1-13.

785. Zercher C, Hunt P, Schuler A, et al. Increasing joint attention, play and language through peer supported play. Autism 2001;5(4):374-98.

**Non-Obtained Studies (N = 30)**

The following studies were not considered for the review due to limitations in our library and retrieval resources.

1. Adrien JL, Hemare E, Blanc R, et al. Study of the cognitive and socio-emotional development of severely autistic children: An evaluative and therapeutic approach [French]. Rev Quebec Psychol 1999;20(1):109-25.

2. Bebbington P, Surrey Oaklands NHS Trust. A pilot study into the effectiveness of social skills training on the social, communication and behavioural characteristics of 8, 9 and 10 year olds with asperger's syndrome: Is it possible to teach "playground skills" to children with asperger's syndrome? Surrey, UK: Surrey Oaklands NHS Trust, 2000.

3. Bettison S. The long-term effects of auditory training on children with autism. J Autism Dev Disord 1997;27(3):347-8.

4. Blanton RL. Sensory discrimination, generalization and language training of autistic children: final report. EnglishNashvile, TN: Vanderbilt University, 2001.

5. Brenn BM. Simultaneous communication and social interaction in the treatment of autism [dissertation]. Evanston (IL): Northwestern University; 1977.

6. Clark ML. Improving the social behavior of siblings of autistic children using a group problem solving approach. Child Fam Behav Ther 1989;11(1):19-33.

7. Da Silva PC, Eira C, Pombo J, et al. Clinical program for treatment of difficulties with relating and communicating, based on the DIR model . Analise Psicol 2003;21(1):31-9.

8. Egel AL, Shafer MS. The use of explicitly trained peers to facilitate the social behavior of autistic children: final report. EnglishCollege Park, (MD): Maryland University, 1983.

9. Fantuzzo JW, Smith CS. Programmed generalization of dress efficiency across settings for a severely disturbed, autistic child. Psychol Rep 1983;53(3):871-9.

10. Fernandes PT, Souza E. Terapia comportamental na síndrome de Asperger: um estudo exploratório [Portuguese]. Temas Desenvolv 2000;(50):19-23.

11. Finkel AS, Williams RL. A comparison of textual and echoic prompts on the acquisition of intraverbal behavior in a six-year-old boy with autism . Anal Verbal Behav 2001;18:61-70.

12. Goncalves Leitao L. Therapeutic relations: an exploratory study of psychoeducational riding and autism . Analise Psicol 2004;22(2):335-54.

13. Gray CA. Social stories and comic strip conversations with students with Asperger syndrome and high functioning autism. Schopler E, Mesibov GB, Kunce LJ, editors. Asperger's syndrome or high-functioning autism? New York: Plenum Press; 1998. p. 167-98.

14. Groden G, Dominque D, Chesnick M. Early intervention with autistic children: a case presentation with pre-program and follow-up data. Psychol Rep 1983;53:715-22.

15. Heimann M. On the effect of multimedia computer programs: gains made by children with autism in reading, motivation, and communication skills. Biennial Meeting of the Society for Research in Child Development; Boston, MA.; 1995.

16. Horowitz LT. Early intervention in autism (0-3)/ South Carolina services and how to access them. JSCMA 2006;102(8):282-4.

17. Kehrer HE. Behaviour therapy in childhood autism. Z Kinder- und Jug-Psych 1974;2(3):233-47.

18. Loncola JA, Craig-Unkefer L. Teaching social communication skills to young urban children with autism. Educ Training Dev Disabil 2005;40(3):243-63.

19. Mancil GR. Functional communication training: a review of the literature related to children with autism. Educ Training Dev Disabil 2006;41(3):213-24.

20. Munro J. Efficacy of speech and language therapy for particular speech sounds in children. Int J Lang Comm Dis 1998;33(Suppl):451-6.

21. Ozonoff S. Assessment and remediation of executive dysfunction in autism and Asperger syndrome. Schopler E, Mesibov GB, Kunce LJ, editors. Asperger's syndrome or high-functioning autism? New York: Plenum Press; 1998. p. 263-92.

22. Palmen A, de Josselin-de Jong K. Applied behavior analysis in the assessment and teaching of self-help skills in individuals with autism: a systematic procedure to assess prompt level. Proceedings of the 7th International Congress Autism Europe: Dreams guide life; Lisbon. FCG; 2004. p. 364-5.

23. Peck CA. Increasing opportunities for social control by children with autism and severe handicaps: effects on student behavior and perceived classroom climate. J Assoc Pers Severe Hand 1985;10(4):183-93.

24. Pioggia G, Igliozzi R, Ferro M, et al. An android for enhancing social skills and emotion recognition in people with autism. IEEE Trans Neural Syst Rehabil Eng 2005;13(4):507-15.

25. Probst P. Effects of group parent training on family interactions and adaptation in families with autistic children. Int J Psychol 2000;35(3-4):201.

26. Richard V, Goupil G. Implementation of the integrated play groups with PDD students [French]. Rev Quebec Psychol 2005;26(3):79-103.

27. Schmidt DC, Franklin R, Edwards JS. Reinforcement of autistic children's responses to music. Psychol Rep 1976;39(2):571-7.

28. Trepagnier CY, Sebrechts MM, Finkelmeyer A, et al. Virtual social environment for preschoolers with autism: preliminary data. Sharkey P, Brooks T, Cobb S 6th International conference: disability, virtual reality and associated technologies; ICDVRAT; Esbjerg, Denmark. University of Reading; 2006. p. 43-52.

29. Whorton DM. Classroom instructional programs with autistic children: group structures and tutoring models: final report. Washington, DC: Department of Education, 1986.

30. Yucel GMA, Cavkaytar A. The effectiveness of a parent education programme offered through distance education about independent autistic children education centre (IACEC). TOJDE 2007;8(1):23-32
